# Synergistic wound healing mechanisms of *Heliotropium curassavicum* extracts via redox modulation, inflammation suppression, and tissue remodeling: linking phytochemical diversity to antioxidant and anti-inflammatory effects

**DOI:** 10.1007/s10787-025-02096-z

**Published:** 2026-02-16

**Authors:** Rania F. Ahmed, Dalia M. Rasheed, Noha A. Mowaad, Rania Elgohary, Eman H. Eltantawy, Eman A. Negm, Mohamed A. Farag, Abdelsamed I. Elshamy

**Affiliations:** 1 Department of Natural Compounds Chemistry, National Research Centre, 33 El Bohouth St., Dokki, Giza 12622 Egypt; 2 Pharmacognosy Department, Faculty of Pharmacy, October 6 University, Central Axis, Part 1/1, Sixth of October, Giza Egypt; 3 Narcotics, Ergogenics and Poisons Department, Medical Research and Clinical Studies Institute, National Research Centre, P.O.12622, Dokki, Giza Egypt; 4 Department of Medical Histology, Faculty of Medicine, Ain Shams University, Cairo, 1181 Egypt; 5 Pharmacognosy Department, Faculty of Pharmacy, Cairo University, Kasr el Aini St., Cairo, 11562 Egypt

**Keywords:** *Heliotropium curassavicum*, Metabolite profiling, Pyrrolizidine alkaloids, Wound healing, Antioxidant activity, Anti-inflammatory

## Abstract

This study assessed the wound-healing potential of *Heliotropium curassavicum *via a comparative metabolite profiling of its ethanol (HC-EtOH), ethyl acetate (HC-EtOAc), and *n*-butanol (HC-BuOH) extracts, alongside in vivo assessment. UHPLC-ESI-MS/MS analysis annotated 86 metabolites, including 13 pyrrolizidine alkaloids (4 of which, viz. uplandicine, uluganine, curassavine, and curassavine N-oxide are newly reported in the genus), 12 phenylpropanoids, and 8 fatty acid amides. Topical application of the extracts accelerated wound closure in a dose-dependent manner, with HC-BuOH exhibiting the strongest activity. HC-BuOH (200 mg/kg) achieved near-complete closure by day 14 and improved healing by 39% compared with Intrasite Gel. This was accompanied by significant modulation of oxidative and inflammatory markers, including a 66% reduction in MDA, a 220% increase in GSH, a 94% reduction in TNF-α, and restoration of PGE-2 toward normal levels (*p* < 0.05). Histopathology confirmed complete re-epithelialization, organized collagen deposition, and localized Ki-67 expression in HC-BuOH–treated wounds. Multivariate data and interaction analyses, supported by PLSR model demonstrated that the superior wound-healing activity of HC-BuOH is attributed for synergistic interactions among pyrrolizidine alkaloids, phenylpropanoids, and fatty acyl amides. These findings highlight *H. curassavicum*, particularly the HC-BuOH fraction, as a promising natural wound-healing agent. These effects are likely attributed to plant richness, in pyrrolizidine alkaloids, phenylpropanoids, and fatty acyl amides, highlighting its potential as a natural wound healing agent.

**Supplementary Information:**

The online version contains supplementary material available at 10.1007/s10787-025-02096-z.

## Introduction

Wounds are actual injuries to the body that occur when the skin cracks or splits due to an internal or external break. Following an injury, the restoration of functioning tissues and disturbed systemic maintenance requires prompt and appropriate wound healing (El-Ashram et al. 2021). The four intricately linked and concurrent phases of the healing process are hemorrhage, infection, replicating, and remodeling of tissue or resolution. Severe pain, inflammation, lack of coordination, and immobilization are frequently observed in chronic wounds. Major economical and quality-of-life issues affect patients suffering from chronic and acute wounds (Chamgordani et al. 2023). Wound healing agents are identified for therapeutic use that are accurate, safe, and affordable. Since ancient times, herbal remedies have been utilized to cure a wide range of illnesses. An estimated 70–80% of the people on the planet receive their medical care from plant-based sources (El-Ashram et al. 2021). Natural therapies made from plants and animals are utilized worldwide to treat both acute and chronic wounds (Elshamy et al. 2020).

*Heliotropium curassavicum* L. (salt/seaside heliotrope), is an annual halophytic herb of the borage family (Boraginaceae), native to the Americas. *Heliotropium* was also introduced in other continents viz*.* Africa, Asia, Europe, and Australia, where it thrives invasively along coastal marches, salty soils, and into inland deserts (Al-Shehbaz 1991; Abd-ElGawad et al. 2019). Hence, under these stress conditions, halophytic plants synthesize a plethora of secondary metabolites, viz*.* alkaloids, and other complex polyphenolics, to adapt to their harsh habitats (Rasheed et al. 2013). These metabolites are of considerable medicinal value for humans, i.e., to treat gonorrhea, inflammation, ulcers, wounds, bacterial infections, and diabetes (Ghori et al. 2016). Phytochemicals isolated and identified from the genus *Heliotropium* include alkaloids (pyrrolizidine alkaloids), phenolic compounds, flavonoids, terpenoids, benzofurans, and quinones (Ghori et al. 2016; Suthar and Solanki 2021) along with the essential oils (Abd-ElGawad et al. 2019).

Pyrrolizidine alkaloids (PAs) are biosynthesized in several higher plant families, viz*.* Boraginaceae, Fabaceae, Orchidaceae, and Asteraceae, where they play a crucial defensive mechanism against herbivores (Ober and Hartmann 1999). Almost all genera of Boraginaceae, including *Heliotropium*, are known to yield this type of alkaloid, and over 190 PAs have been identified within 220 species of this family (El-Shazly and Wink 2014). These plant secondary metabolites are commonly recognized as potent plant mutagens and hepatotoxins that have serious implications on human health (Chain 2011). The European Medicine Agency (EMA) set a daily threshold of 1.0 μg of PA as a maximum exposure limit for adults to avoid carcinogenic risks (EMA 2014). Hence, such limitations impose constraints and necessary precautionary measures for the safe and proper systemic use of PA enriched plants in therapeutic practice.

This study aimed to unravel the chemical and therapeutic potential of *H. curassavicum* through comprehensive metabolomic characterization and pharmacological assays. By employing UHPLC-ESI-MS/MS in both positive and negative ionization modes, an in-depth coverage of *H. curassavicum* extracts (ethanol, ethyl acetate, and butanol) metabolome is provided, with emphasis on identifying phytochemicals linked to envisaged bioactivity. Concurrently, we evaluated the wound healing efficacy of these extracts in a full-thickness excisional rat model to elucidate their antioxidant, anti-inflammatory, and tissue-regenerative action mechanisms. This integrative approach aimed to bridge the gap between phytochemical complexity and biological effects, positioning *H. curassavicum* as a promising candidate for plant-based wound management strategies.

## Materials and methods

### Plant materials, authentication, and extraction

The aerial parts of *H. curassavicum* were collected on May of 2023 throughout the flowering period from the northern coast of the Mediterranean, Gamasa (31°27′36.9″N 31°27′08″E), Dakahlia Governorate, Egypt. Voucher specimen within a certificate of authenticity, Mans. 002374001, was added to the Mansoura University, Faculty of Science herbarium after the species was validated and verified by Dr. Ahmed M. Abd-ElGawad, a professor of plant ecology at the university.

After being thoroughly cleaned of filth, the gathered *H. curassavicum* aerial parts were placed in an open, perfectly dry, and shaded area for two weeks until they dried entirely. A sterile plant grinder was used to grind the dried plant material into a fine powder. From the total amount of powdered plant material (1200 g), three equally weighted portions (400 g each) were individually extracted within the three extraction solvents, ethanol-H_2_O (7:3; HE-EtOH, 5 L), ethyl acetate (HE-EtOAc, 5 L), 70% and *n*-butanol (HE-BuOH, 5 L), for one week. Following filtering and reduced pressure by rotavapor at 45–50 °C, three distinct dark brown gum weighed 19.5, 24.2, and 17.4 g, respectively. Until further testing or analysis, the extracts were stored in three opaque glass vials at a temperature of –4 °C.

## UPHPLC-ESI-MS/MS analysis of *H. curassavicum* extracts

The air-dried powder of *H. curassavicum* (HC) was dried in the shade, pulverized, and 10 mg of the fine plant was extracted by sonication in 5 mL of solvents of different polarities viz*.* ethanol-H_2_O (7:3; HE-EtOH), ethyl acetate (HE-EtOAc), and *n*-butanol (HE-BuOH). Each fraction was separately filtered and concentrated under reduced pressure using a rotary evaporator (Buchi, Switzerland) until completely dry, then freeze dried for UPLC-MS analysis. For profiling of different HC extracts, 10 µg of each dried extract was separately redissolved in 2 mL 70% alcohol containing 1 ug/ mL umbelliferone (internal standard) by sonication, centrifuged and filtered (0.22 µm). Clear extracts aliquots (2 µL) were injected on a HSS T3 column (100 × 1.0 mm, 1.8 µm particle size) installed on an ACQUITY UPLC system (Waters, Milford, MA, USA) supplied with an ESI interface, operating in both positive and negative ion mode under same condition cited in (Rasheed et al. 2023). Metabolites’ assignment was based on their UV spectra, their high-resolution masses and predicted chemical formulae, R_t_ data, in addition to examination of MS^2^ spectra in both ionization modes, comparison with recorded reference literature and phytochemical dictionary of natural products (CRC, Wiley) (Taher et al. 2025; El-Kashak et al. 2025).

### Wound healing bioassay animals

Male Albino Wistar rats, each weighing between 180 and 200 g and aged approximately 8–10 weeks, were obtained from the well-established animal house colony of the National Research Centre, Cairo, Egypt. Upon arrival, the animals were housed in clean polypropylene cages under standard laboratory conditions, including a controlled temperature of 22 ± 2 °C, relative humidity of 50–60%, and a 12-h light/dark cycle. All rats were acclimatized for one week before the commencement of the experiment to minimize stress and to ensure physiological stabilization. During the entire experimental period, animals had free access to a standard commercial pellet diet formulated to meet the nutritional requirements of laboratory rodents, along with clean drinking water provided ad libitum. All experimental procedures involving animal handling and treatment were conducted in strict accordance with the ethical guidelines for the care and use of laboratory animals and were approved by the Research Ethics Committee of the Faculty of Medicine, Ain Shams University, Cairo, Egypt. The study protocol was reviewed and granted ethical clearance under Approval Number: FWA 000017585.

### Experimental design and wound induction

A uniform semisolid delivery system was developed using a hydrophobic ointment base comprising white paraffin, cetostearyl alcohol, wool fat, and hard paraffin. This base conformed to the criteria outlined for topical semisolid formulations according to established pharmacopeial references (Abeje et al. 2022). The plant-derived extracts—HC-EtOH, HC-EtOAc, and HC-BuOH—were incorporated into this matrix at two dose levels, 10% and 20% w/w (equivalent to 100 and 200 mg/kg, respectively), to produce the respective medicated ointments.

Male Wistar albino rats were randomly assigned to 9 groups (8 per group) to evaluate the wound healing potential of various plant extract fractions. The grouping and treatments were as follows: Group 1 (Negative Control): Healthy, non-wounded rats that did not receive any treatment, serving as the baseline control. Group 2 (Wounded Control): Rats received a full-thickness excision wound and were treated topically with the ointment base (Vaseline) alone (Abeje et al. 2022). Group 3 (Reference Group): Wounded rats treated with Intrasite gel, a standard wound-healing formulation, serving as a positive control. Groups 4 and 5 (HC-EtOH-treatment): Wounded rats were treated topically with a hydroethanolic extract ointment-base formulation (HC-EtOH-treatment) at doses of 100 and 200 mg/kg/day, respectively. Groups 6 and 7 (HC-EtOAc-treatment): Wounded rats treated with ethyl acetate extract ointment-base formulation (HC-EtOAc) at 100 and 200 mg/kg/day, respectively. Groups 8 and 9 (HC-BuOH-treatment): Wounded rats treated with butanol extract ointment-base formulation (HC-BuOH) at 100 and 200 mg/kg/day, respectively.

Before wound induction, all animals were anesthetized via intraperitoneal injection (i.p.) of ketamine (87.5 mg/kg) and xylazine (12.5 mg/kg). The dorsal surface of each rat was shaved using an electric clipper and disinfected with 70% ethanol. A circular, full-thickness excision wound approximately 2.00 cm in diameter was created on the mid-dorsal region using a standardized round template, as illustrated in Figure S1. Such protocol ensured uniform wound size across all experimental groups. Topical treatments were administered once daily for 14 consecutive days. The progression of wound healing was monitored by measuring wound contraction on alternate days using planimetric analysis. On day 14, all animals were humanely sacrificed under anesthesia, and skin tissue samples from the wound site were excised for subsequent biochemical and histopathological evaluations.

### Assessment of wound size

Wound healing was assessed by measuring the reduction in wound area at 3, 7, 10, and 14 days after the start of the experiment. The relative reduction in wound area was calculated using the following equation (Osman and Amin 2020).

$$\text{Relative reduction in wound area }\left(\mathrm{\%}\right)=\frac{A0-At}{At} X 100$$

where, A_o_ and A_t_ are the wound area at zero time and time (t), respectively.

### Biochemical analysis

Fourteen days post-wounding, rats were sacrificed, and wounded tissues from different groups were collected. A homogenizer was used to prepare 20% (w/v) homogenates in phosphate buffer (pH 7.4). The homogenates were then centrifuged for 10 min at 1000 rpm and 4 °C by means of a cooling centrifuge (Sigma, Germany Co., Ltd). Supernatants were collected for different biochemical studies.

### Assessment of oxidative stress parameters

#### Determination of MDA level in skin

Malondialdehyde (MDA) was measured using Uchiyama and Mihara’s technique (Uchiyama and Mihara 1978). The assay relies on colorimetric measurement of a pink product formed when one molecule of MDA reacts with two molecules of thiobarbituric acid at low pH for 45 min at 95 °C. MDA nanomoles per gram of tissue were used to measure lipid peroxidation.

#### Determination of skin content of GSH

Using the Ellman reagent (5, 5′-dithiobis [2-nitrobenzoic acid]), both protein and nonprotein SH groups react to create 1 mol of 2-nitro-5-mercaptobenzoic acid for every mole of sulfhydryl to assess the glutathione (GSH) concentration in gastric mucosal homogenates (SH). At 412 nm, the nitromercaptobenzoic acid may be detected spectrophotometrically and has a bright yellow hue. Protein SH groups needed to be precipitated before the Ellman reagent was added in order to measure the GSH level in tissue. In terms of micromoles per gram of tissue, GSH content in the stomach mucosal homogenate was expressed (Woolliams et al. 1983).

#### Determination of skin TNF-α and PGE2 content

Skin content of tumor necrosis factor alpha (TNF-α) (Sigma-Aldrich, Millipore, Roche. (Cat.no. RAB0479), and Prostaglandin E2 (PGE2) (Elabscience, Houston, TX, USA. Cat.no. E-EL-0034) were determined using enzyme-linked immunosorbent assay (ELISA). All biochemical assays were performed following the manufacturer’s instructions.

#### Skin histopathological and immunohistochemical studies

Skin samples from the different experimental groups were fixed in 10% neutral-buffered formalin, processed routinely, and embedded in paraffin. Tissue blocks were sectioned at a thickness of 5 µm. Sections were stained with hematoxylin and eosin (H&E) for general histological evaluation, and with Verhoeff’s stain to visualize collagen and elastic fibers in the dermis. For assessment of cellular proliferation, immunohistochemical staining for Ki-67 was performed. All stained sections were examined using a light microscope.

#### Morphometrical and statistical analysis

Quantitative analysis included measurement of the mean epidermal thickness at the wound site or, when absent, at the wound margins. Additionally, the mean percentage area of Ki-67 immunoreactivity per high-power field (HPF) was calculated. Statistical analysis was conducted to compare results across experimental groups. Data are expressed as mean ± standard deviation (SD). Data analysis was performed using one-way ANOVA followed by Dunnett’s multiple comparisons test. The difference was considered statistically significant at *p* < 0.05.

#### Potential synergistic interactions experiments

In silico datasets were created to simulate metabolite abundances and predict impacts on wound-healing outcomes and related biomarkers in order to assess potential synergistic interactions among the key metabolite classes in *H. curassavicum* extracts. The main dependent variable was the percentage of wound healing, and the connected secondary responses were oxidative stress and inflammatory markers (MDA, GSH, TNF-α, and PGE2). A combined effect of two or more metabolite classes that was greater than the anticipated additive effect, determined from individual contributions, was referred to as synergy.

In order to create hierarchical regression models, metabolite classes were included as main-effect predictors, and then interaction terms between metabolite classes. Evidence of synergy was defined as a statistically significant interaction (*p* < 0.05) that showed a more protective biomarker shift or a greater improvement in wound healing. Interaction plots were created to show nonadditive deviations from main-effect trends, and metabolite levels were separated into low and high groups using a median split to facilitate understanding. For identifying nonlinear and nonadditive synergistic patterns across metabolite-class combinations, multifactor dimensionality reduction (MDR) was employed. After tenfold cross-validation of MDR models, combinations that showed more prediction accuracy for wound-healing outcomes than any single metabolite class were categorized as synergistic. High-effect and low-effect metabolite combinations were highlighted in a heatmap that displayed the resulting patterns.

Synergy-related discoveries were integrated across the system via network analysis. Metabolite classes or biomarkers were represented by nodes in weighted, undirected graphs, whereas interaction-based associations or strong partial correlations were represented by edges. Higher weights were assigned to edges that corresponded to synergistic interactions, enabling the emergence of synergistic clusters through modularity patterns.

The multivariate associations between metabolite profiles and biological responses were then evaluated using partial least squares regression (PLSR). The first two latent components were displayed to show clustering patterns between metabolite classes and biological markers, providing additional visual evidence of synergistic alignment contributing to better wound-healing results. All variables were scaled and mean-centered. NumPy, pandas, scikit-learn, matplotlib, and NetworkX were used in Python for all computational analyses and visualizations (Duarte and Vale 2022; Ma and Motsinger-Reif 2019).

## Results

### UHPLC-ESI-MS/MS metabolite profiling of H. curassavicum extracts

**Fig. 1:**
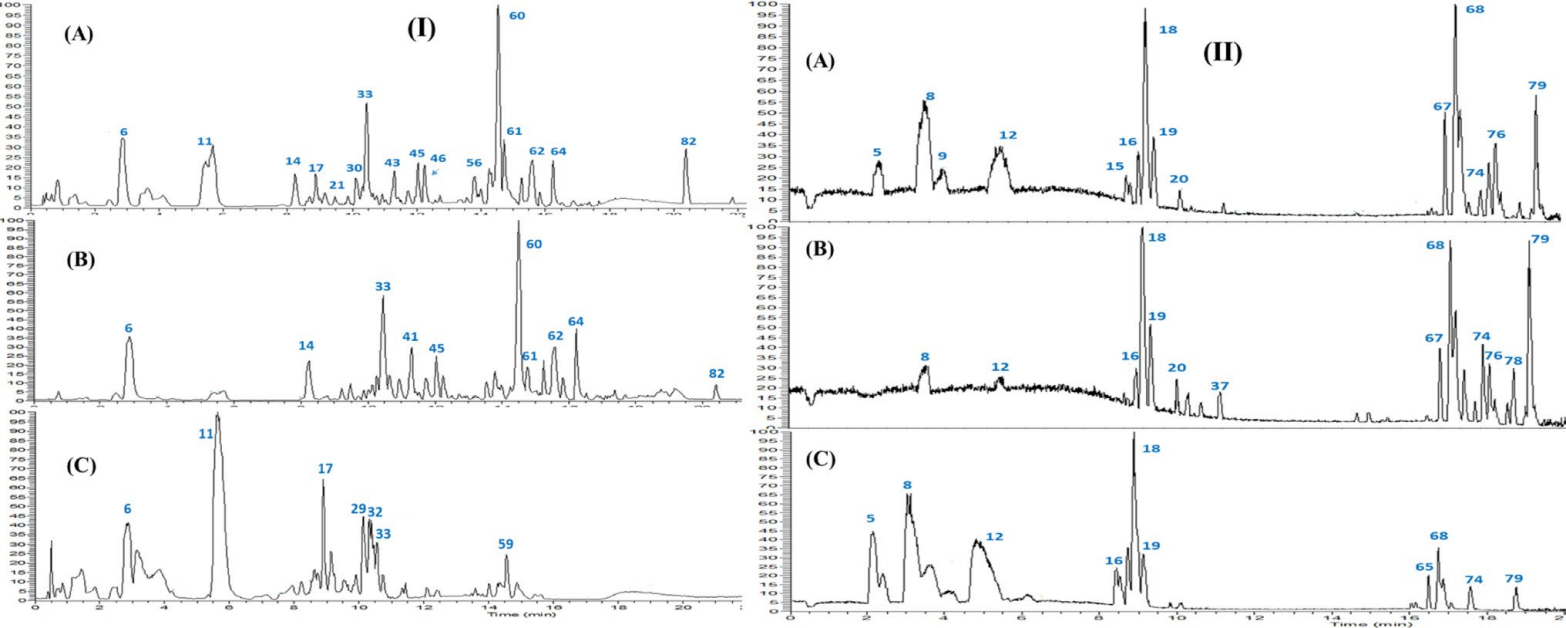
Representative UPLC-ESI-MS base peak chromatograms of **A**: 70% alcohol, **B**: ethyl acetate and **C**: *n*-butanol extracts of *Heliotropium curassavicum* extracts in (**I**) negative and (**II**) positive ionization modes. Peaks numbering follow those listed in Table 1 for metabolite identification. Macroscopic morphology of the healing progress of excision wound model in the control untreated group, different *Heliotropium curassavicum* extracts treated groups at 0, 3, 7,10 and 14 days post-wounding.

**Fig. 2:**
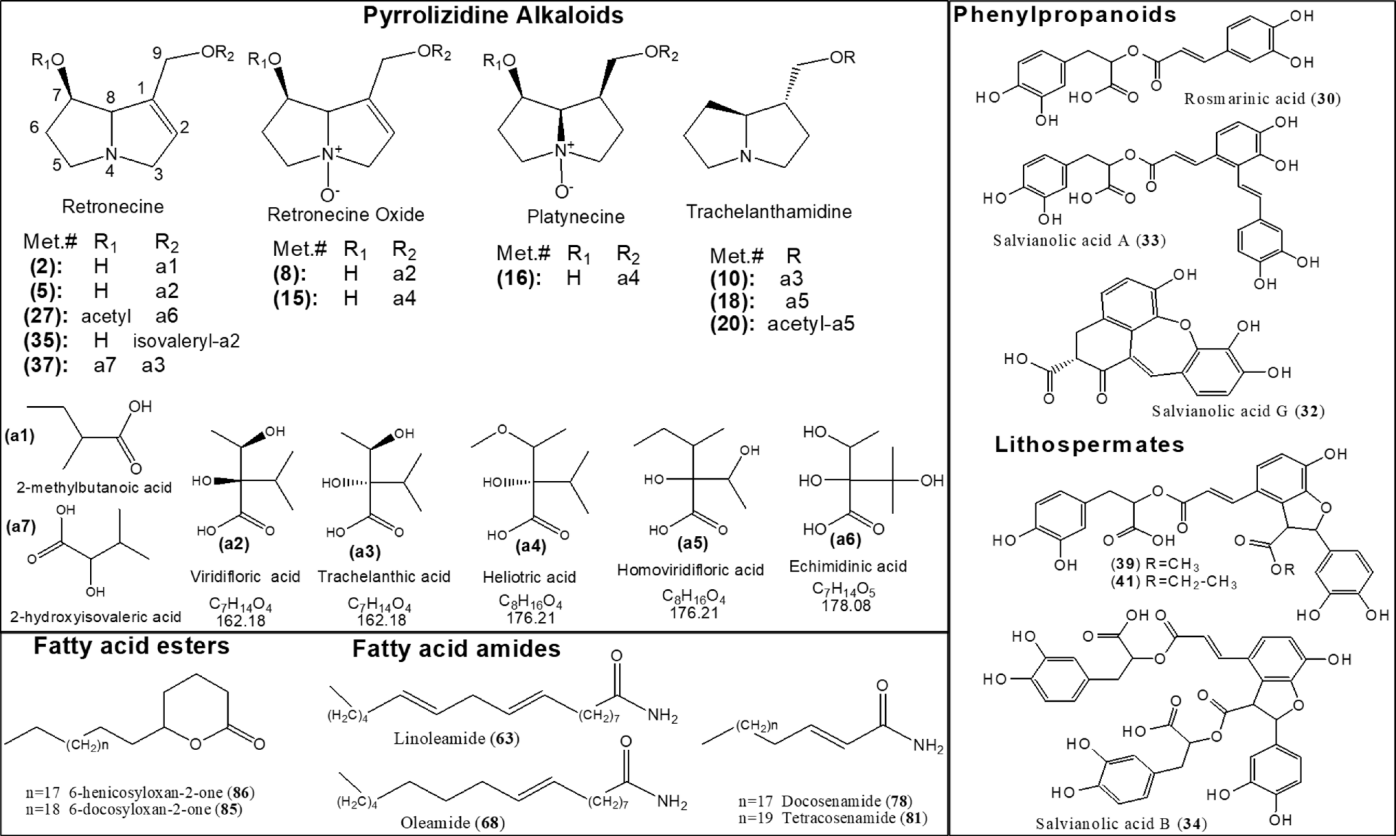
Major classes of metabolites identified using UPLC-ESI-MS/MS analysis in *Heliotropium curassavicum* L. extracts. Compounds numbering in brackets follow those listed in Table 1 for metabolites identification

UHPLC-ESI-MS/MS analysis was used to discern the metabolic profile and reveal bioactive composition in three extracts of *H. curassavicum, *viz. HC-EtOH, HC-EtOAc, and HC-BuOH extracts, in an untargeted manner. The analytical procedure employed herein allowed for the elution of analytes within 25 min in an order of decreasing polarity (Fig. 1). A total of 86 metabolites were annotated in both positive and negative ionization modes, including 13 pyrrolizidine alkaloid, 12 phenylpropanoids, 3 naphthoquinones, 19 fatty acids, 8 fatty acyl amides and 4 fatty acid esters. Assigned metabolites are listed in Table 1 and structures of main metabolites are illustrated (Fig. 2). Details of assigning the major secondary metabolites’ classes within *H. curassavicum* extracts will be explained in the upcoming subsections.

**Table 1:** Assigned metabolites in *Heliotropium curassavicum* extracts via UPLC-ESI-MS/MS in negative and positive ionization modes

Met. #	R_t_	Identification	Emperical formula	Class	Mol. Ion* m/z*		∆ mass	MS^2^ ions		Extract (s)
					[M−H]^−^	[M+H]^+^	(ppm)	(−ve)	(+ve)	
1	0.50	Coriose (Altro-heptulose)	C_7_H_14_O_7_	Sugar	209.0669		6.22	191, 159, 129		ETOH
2	1.13	7-(2-methylbutyryl) retronecine	C_13_H_21_NO_3_	Pyrrolizidine alkaloid	238.8918	240.1602	0.58	398, 220, 158		EtOH, EtOAc, BuOH
3	1.18	Dihydroxyphenyllactic acid (danshensu)	C_9_H_10_O_5_	Phenylpropanoids	197.0456		6.09	179		EtOAc
4	2.19	Syringaldehyde	C_9_H_10_O_4_	Phenolic acid	181.0507		6.43	163, 135		EtOAc
5	2.21	Lycopsamine (9-viridiflorylretronecine)	C_15_H_25_NO_5_	Pyrrolizidine alkaloid		300.1813	2.63		156, 138, 120	EtOH, BuOH
6	2.36	Cymarose (methyl-dideoxyhexose)	C_7_H_14_O_4_	Sugar	161.0821		1.35	117, 99		EtOH, BuOH
7	2.40	Ammonium gluconate	C_6_H_15_NO_7_	Amino sugar acid		214.0904	− 8.12		158, 141	EtOH, EtOAc, BuOH
8	3.42	Lycopsamine (Intermedine) -*N*-oxide	C_15_H_25_NO_6_	Pyrrolizidine alkaloid		316.1762	2.17		272, 172, 138	ETOH
9	4.01	*O*-suberoylcarnitine	C_8_H_15_NO_3_	Acyl quaternary ammonium salt		318.1918	2.25		174	EtOH, BuOH
10	4.39	Heliovicrine	C_15_H_27_NO_4_	Pyrrolizidine alkaloid		286.2019	2.14		242, 142, 124	BuOH
11	5.07	Caffeic acid	C_9_H_8_O_4_	Phenylpropanoids	179.0351		6.95	135		EtOAc
12	5.65	Floridimine	C_15_H_27_NO_5_	Pyrrolizidine alkaloid		302.1970	2.71		158	EtOH, EtOAc, BuOH
13	7.76	hydroxy-polyrenylbenzoate	C_17_H_22_O_3_	Phenolic acid	273.0075		− 5.03	193		EtOH, EtOAc, BuOH
14	8.13	Ethyl-dihydroxy-methylpentanoic acid	C_8_H_16_O_4_	Organic acid	175.0977		0.84	131, 113		EtOH, EtOAc, BuOH
15	8.43	Heliotrine -*N*-oxide	C_16_H_27_NO_6_	Pyrrolizidine alkaloid		330.1918	1.98		286, 172, 138	EtOH, EtOAc, BuOH
16	8.53	Europine A	C_16_H_29_NO_6_	Pyrrolizidine alkaloid		332.2072	1.34		288, 174	EtOH, EtOAc, BuOH
17	8.84	Rutin	C_27_H_30_O_16_	flavonol glycoside	609.1457		− 0.59	417, 337, 301		EtOH, EtOAc
18	8.86	Curassavine	C_16_H_29_NO_4_	Pyrrolizidine alkaloid		300.2175	1.98		256, 142, 124	EtOH, EtOAc, BuOH
19	9.10	Curassavine -*N*-oxide	C_16_H_29_NO_5_	Pyrrolizidine alkaloid		316.2126	4.39		158	EtOH, EtOAc, BuOH
20	9.28	Floridine	C_17_H_29_NO_6_	Pyrrolizidine alkaloid		344.2077	2.63		326, 284, 158	EtOH, EtOAc, BuOH
21	9.35	Isopentenyl oxy-psoralene (Imperatorin)	C_16_H_14_O_4_	Psoralene	269.0821		4.55	159, 109		EtOAc
22	9.40	Spermidine	C_7_H_19_N_3_	Polyamine	144.0458		9.85	126, 100		EtOAc
23	9.49	Indole carboxaldehyde	C_9_H_7_NO	Indole	144.0458		9.64	125, 102		EtOH
24	9.52	hydroxybenzoic acid	C_17_H_6_O_3_	Phenolic acid	137.0247		− 9.35	119, 93		EtOH, EtOAc
25	9.64	Hesperetin	C_16_H_14_O_6_	Flavanone	301.0718		3.90	273, 257		EtOAc
26	9.90	Dicoumaroylspermidine	C_25_H_31_N_3_O_4_	Polyamine	436.2240		− 0.91	316		EtOH, EtOAc
27	10.05	Uplandicine	C_17_H_27_NO_7_	Pyrrolizidine alkaloid		358.2233	2.47		340, 298, 158	EtOH, EtOAc, BuOH
28	10.25	Methyl suberic acid	C_9_H_16_O_4_	Fatty acid ester	187.0977		6.75	125		EtOH
29	10.34	Asebotin	C_15_H_30_O_15_	Dihydrochalcone glucoside	449.1486		− 3.22	257, 241		EtOH, BuOH
30	10.41	Rosmarinic acid	C_18_H_16_O_8_	Phenylpropanoids	359.0774		0.58	197, 161		EtOH, EtOAc
31	10.42	Lithospermic acid	C_27_H_22_O_12_	Phenylpropanoids	537.1040		0.39	519		EtOH, EtOAc
32	10.50	Salvianolic acid G	C_18_H_12_O_7_	Phenylpropanoids		341.0665	2.78		295, 253	EtOAc
33	10.55	Salvianolic acid A	C_26_H_22_O_10_	Phenylpropanoids	493.1140		0.10	295		EtOH, EtOAc, BuOH
34	10.70	Salvianolic acid B (Lithospermic acid b)	C_36_H_30_O_16_	Phenylpropanoids	717.1456		− 0.67	519, 339		EtOH, EtOAc
35	10.75	Isovaleryl-viridifloryl retronecine	C_20_H_33_NO_6_	Pyrrolizidine alkaloid		384.2391	2.61		282, 138	EtOAc
36	10.87	Echinone	C_19_H_20_O_6_	Naphthalenedione	343.0823		3.15	325, 179, 161		EtOAc
37	10.98	Uluganine	C_20_H_33_NO_7_	Pyrrolizidine alkaloid		400.2336	1.65		382, 316, 298	EtOH, EtOAc
38	11.01	Unknown lithospermic acid derivative	C_36_H_30_O_15_		701.1507		− 0.62	519, 503, 321		EtOH, EtOAc
39	11.05	methyl lithospermate	C_28_H_24_O_12_	Phenylpropanoids	551.1192		− 0.43	353, 321		EtOH, EtOAc, BuOH
40	11.20	Ethyl caffeate	C_11_H_12_O_4_	Phenylpropanoids	207.0665		0.91	179, 161, 135		EtOH
41	11.39	dimethyl lithospermate	C_29_H_26_O_12_	Phenylpropanoids	565.1349		− 0.46	519, 367, 321		EtOH, EtOAc
42	11.60	Eritrichin 3-(3,4-dihydroxyphenyl)-2-[4-(3,4-dihydroxyphenyl)-6,7-dihydroxynaphthalene-2-carbonyloxy]propanoic acid	C_26_H_20_O_10_	Lignan	491.0984		2.21	311, 293		EtOAc
43	11.74	Trihydroxy-octadecadienoic acid (Malyngic acid)	C_18_H_32_O_5_	Fatty acid	327.2177		− 0.05	291, 229, 171		EtOH, EtOAc
44	11.98	Trihydroxyoctadecanoic acid	C_18_H_36_O_5_	Fatty acid	331.2490		0.16	313		EtOH, EtOAc
45	12.16	Trihydroxy octadecenoic acid	C_18_H_34_O_5_	Fatty acid	329.2335		3.86	311, 229, 171		EtOH, EtOAc, BuOH
46	12.25	Alkannin	C_16_H_16_O_5_	naphthoquinone	287.2229		0.51	269, 241		EtOH, EtOAc
47	12.35	Hydroxy octadecatrienoic acid	C_18_H_30_O_3_	Fatty acid	293.2124		0.62	263, 251		EtOH, EtOAc
48	12.63	Hydroperoxy linolenic acid	C_18_H_30_O_4_	Fatty acid	309.2072		3.89	291, 251, 171		EtOH, EtOAc
49	12.64	6-[7-hydroxy-4-methyl-3-(propan-2-ylidene) heptyl] naphthalene-1,4-dione (Cordiaquinone A)	C_21_H_26_O_3_	Naphthoquinone	325.2023		4.05	307, 289, 201		EtOAc, BuOH
50	12.79	Dioxo octadecadienoic acid	C_18_H_28_O_4_	Fatty acid	307.19159		0.35	289, 235, 185		EtOH, EtOAc
51	12.91	Unknown Naphthoquinone derivative	C_21_H_24_O_6_		371.2441		3.48	329, 311		EtOAc
52	13.16	octyl ferulate	C_18_H_26_O_4_	Phenylpropanoids	305.1759		3.72	287, 249		EtOH, EtOAc
53	13.33	Euscaphic acid	C_30_H_48_O_5_	Pentacyclic ursane triterpenoid	487.3432		2.93	409, 329		EtOAc
54	13.62	Dictamnoside G	C_27_H_46_O_14_	Sesquiterpene diglycoside	593.2726		5.89	413, 315, 277		EtOH, BuOH
55	13.81	Unknown triterpene/ diterpene glycoside	C_30_H_42_O_11_		577.2685		5.40	299, 225		EtOH, EtOAc, BuOH
56	13.84	Trigonotin C	C_33_H_38_O_18_	Aryldihydronaphthalene-type Lignan	721.3646		− 0.90	397		EtOH, EtOAc
57	14.21	Ethyl Lithospermate	C_30_H_28_O_12_	Phenylpropanoids	579.2844		7.65	299, 279, 225		EtOAc
58	14.35	Stearidonic acid	C_18_H_28_O_2_	Fatty acid		277.2169	2.36		259, 149, 135	ETOH
59	14.39	Viteoside A	C_28_H_44_O_11_	Labdane-type diterpene glycoside	555.2842		5.54	299, 225		EtOH, EtOAc, BuOH
60	14.69	Hydroxyoctadecadienoic acid	C_18_H_32_O_3_	fatty acid	295.2279		0.35	277, 195, 171		EtOH, EtOAc
61	15.05	Methyl linoleate	C_19_H_34_O_2_	Fatty acid		295.2272	1.35		277	EtOAc
62	15.50	OctadecatetrEtOHnoic acid (Stearidonic acid)	C_18_H_28_O_2_	Fatty acid		277.2170	2.89		259, 149	EtOAc
63	16.03	Octadecadienamide (Linoleamide)	C_18_H_33_NO	Fatty acid amide		280.2643	2.71		263, 245	EtOH, EtOAc, BuOH
64	16.20	Hydroxyhexadecanoic acid (hydroxy-palmitic acid)	C_16_H_32_O_3_	Fatty acid	271.2278		− 0.21	225		EtOH
65	16.50	Onosmin b (methyl 2-[(4-methylbenzyl) amino] benzoate)	C_16_H_17_NO_2_	Aminobenzoic acid		256.2641	2.38		256, 116, 102	EtOH, EtOAc, BuOH
66	16.53	Hydroxy octadecenoic acid (Ricinoleic acid)	C_18_H_34_O_3_	Fatty acid	297.2437	299.1863	0.64/3.37	251	271, 236	EtOH, EtOAc
67	16.59	Hexadecanamide (Palmitamide)	C_16_H_33_NO	Fatty acid amide		256.3412	2.73		130, 116, 88	EtOH
68	16.67	Octadecenamide (Oleamide)	C_18_H_35_NO	Fatty acid amide		282.2798	2.16		265, 247	EtOH, EtOAc, BuOH
69	16.75	Hydroxy nonadecanoic acid	C_19_H_38_O_3_	Fatty acid	313.2751		4.37	283		EtOH
70	16.79	Methyl-hydroxyhexadecanoate	C_17_H_34_O_3_	Fatty acid	285.2436		0.46	239		EtOH, EtOAc
71	16.96	Alkannin angelate	C_21_H_22_O_6_	Naphthoquinone	369.3013		3.61	351, 307		EtOAc
72	17.34	Hydroxy octadecanoic acid (stearic acid)	C_18_H_34_O_3_	Fatty acid	299.2592		− 0.03	253		EtOH, EtOAc
73	17.38	Hydroxy eicosanoic acid	C_20_H_40_O_3_	Fatty acid	327.2904		3.05	309, 183		ETOH
74	17.54	Octadecanamide (Stearamide)	C_18_H_37_NO	Fatty acid amide		284.2957	3.13		102, 88	EtOH, EtOAc, BuOH
75	17.59	Shimaurinoside B	C_16_H_30_O_10_	Megastigmane glycoside	381.1737		− 4.76	363, 337, 319		EtOH, EtOAc
76	17.70	N-Palmitoylpyrrolidine	C_20_H_39_NO	Pyrrolidine		310.3115	3.38		293, 275	EtOH, EtOAc, BuOH
77	18.20	Bis(hydroxymethyl) butyl docosanoate	C_28_H_56_O_4_	Fatty acid ester	455.4103		1.74	437, 409		EtOH
78	18.63	Icosanamide	C_20_H_41_NO	Fatty acid amide		312.3271	3.19		102	EtOH, EtOAc
79	18.69	Docosenamide (Erucamide)	C_22_H_43_NO	Fatty acid amide		338.3427	2.68		321, 303	EtOAc, BuOH
80	19.88	Docosanamide (Behenamide)	C_22_H_45_NO	Fatty acid amide		340.3581	1.96			EtOH, EtOAc
81	19.92	Tetracosenamide (Nervonamide)	C_24_H_47_NO	Fatty acid amide		366.3739	2.34		349, 331	EtOAc
82	20.30	Hydroxy hexacosanoic acid	C_26_H_52_O_3_	Fatty acid	411.3842		− 0.43	365, 337, 323		EtOH, EtOAc
83	20.59	Hydroxy tetracosanoic (Lignoceric) acid	C_24_H_48_O_3_	Fatty acid	383.3527		1.79	337		EtOAc
84	21.80	Hydroxy octacosanoic acid	C_28_H_56_O_3_	Fatty acid	439.4152		1.41	421, 393, 365		EtOH, EtOAc
85	22.20	6-docosyloxan-2-one	C_27_H_52_O_2_	Fatty acid ester		409.4045	1.30		391, 377	EtOAc
86	22.45	6-henicosyloxan-2-one	C_26_H_50_O_2_	Fatty acid ester		395.3887	0.94		377	EtOAc

EtOH; Ethanol extract, EtOAc; ethyl acetate extract, BuOH; *n*-butanol extract

### Pyrrolizidine alkaloids

*Heliotropium* genus is known to produce pyrrolizidine alkaloids (PAs), including 13 PAs annotated using UPLC-ESI-MS in positive ionization mode, of which 4 (**2**, **16**, **27**, **37**) are reported in the genus *Heliotropium* for the first time. The structural composition of PAs comprises a necine motif (pyrrolizidine-based amino alcohol) in a tertiary amine or *N*-oxide state, and with or without a degree of unsaturation at the 1,2 positions. Necine also affords 2 possible hydroxylation states; either directly attached to the heterocyclic moiety at position C-7, or indirectly attached as a hydroxymethyl group at position C-1 (Fig. 2). Thereby, esterification with various aliphatic organic acids at C-7 or C-9 of necine is common, generating largely diverse structures of open-chain mono-, di- or macrocyclic PA esters (El-Shazly and Wink 2014). PAs can be broadly categorized into 4 major groups, viz. retronecine-, heliotridine-, platynecine -, and otonecine- type alkaloids (Tamariz et al. 2018).

Identified PAs in *H. curassavicum* extracts were esters of retronecine- or, its diastereomeric isomer, heliotridine-type bases (**2, 5, 8, 15, 27, 35, 37**), trachelanthamidine-type (**10, 12, 18, 19, 20**) and platynecine-type (**16**) with necic acid components (heliotrinic, viridifloric or its diastereomer trachelanthic acids) (Fig. 2) aided by fragmentation pattern. For example, MS^2^ spectrum of lycopsamine (**5**) (also known as indicine or 9-viridiflorylretronecine, *m*/*z* 300.1813 [M + H]^+^, t_R_ = 2.21 min, [C_15_H_26_NO_5_]^+^) showed a typical fragmentation pattern of retronecine core with a base peak at *m/z* 138 [C_8_H_12_NO]^+^ implying for an unsaturated necine with a C-7 hydroxyl group (supinidine) after the neutral loss of 162 amu for viridifloric acid (Suppl. Fig. S2). Additional characteristic fragment ions indicative of retronecine were observed at *m/z* 156 [C_8_H_14_NO_2_]^+^ (heliotridine) and 120 [C_8_H_10_N]^+^, separated by 18 amu from the base peak, implying successive loss of two water molecules (Tsiokanos et al. 2023). Lycopsamine is regarded as a potential biomarker for the consumption of borage-containing food products, and was isolated previously from genus *Heliotropium* (Roeder et al. 1991). Its *N*-oxide derivative, lycopsamine (Intermedine)-*N*-oxide, was assigned to molecular ion (**8**) (t_R_ = 3.42 min, [C_15_H_26_NO_6_]^+^) at *m*/*z* 316.1762 [M + H]^+^ in HC-EtOAc extract. Its MS^2^ spectrum revealed a different base peak ion at *m/z* 172 [C_8_H_14_NO_3_]^+^, implying for a protonated retronecine-*N*-oxide ion, alongside fragment ions at *m/z* 155, 138 and 120 appearing as much less intense signals (Suppl. Fig. S3). Another retronecine-*N*-oxide was assigned for heliotrine-*N*-oxide (**15**) at *m*/*z* 330.1918 [M + H]^+^ (t_R_ = 8.43 min, [C_16_H_28_NO_6_]^+^) showing same base peak at *m/z* 172 [C_8_H_14_NO_3_]^+^ after the loss of 158 amu representing a heliotrinyl fragment (Suppl. Fig. S4). Europine A (**16**), is the only platynecine-type PA detected as an *N*-oxide derivative in all of *H. curassavicum* extracts herein for the first time (*m*/*z* 332.2072 [M + H]^+^, t_R_ = 8.53 min, [C_16_H_30_NO_6_]^+^), and can be regarded as a reduced form of heliotrine-*N*-oxide (**15**, C_16_H_27_NO_6_) (Suppl. Fig. S5), which has yet to be confirmed using other spectroscopic tools i.e., NMR. Two retronecine di-esters uplandicine (**27**) and uluganine (**37**) (*m*/*z* 358.2233 [M + H]^+^, [C_17_H_28_NO_7_]^+^ and *m*/*z* 400.2336 [M + H]^+^, [C_20_H_34_NO_7_]^+^, respectively) were detected in the MS spectra of *H. curassavicum* extracts for the first time in this study, and previously isolated in other borages (El-Shazly et al. 1999). Uplandicine is a diester of retronecine with echimidinic and acetic acids, whereas uluganine is esterified with trachelanthic and 2-hydroxyisovaleric acids and their MS^2^ spectral analysis displayed fragment ion signals at *m*/*z* 156, 138 and 120, corresponding to heliotridine, supinidine and its dehydrated form as explained previously (Suppl. Figs. S6 & S7).

The chemotaxonomic marker PA of *H. curassavicum* is curassavine (**18**), an ester of trachelanthamidine with homoviridifloric acid (3-carboxy-4-methylhexane-2,3-diol), appeared as a major peak in the MS chromatogram of the three extracts at *m*/*z* 300.2175 [M + H]^+^ (t_R_ = 8.86 min, [C_16_H_30_NO_4_]^+^) followed by its *N-*oxide derivative (**19**) at *m*/*z* 316.2126 [M + H]^+^ (t_R_ = 9.10 min, [C_16_H_30_NO_5_]^+^) (Fig. 1). The MS^2^ spectra of curassavine exhibited a base peak at *m*/*z* 142 representing the protonated form of the alkaloid trachelanthamidine [C_8_H_16_NO]^+^, and a less abundant signal at *m*/*z* 124 for its dehydrated form [C_8_H_16_N]^+^, whereas curassavine-*N*-oxide showed a base peak at *m*/*z* 158 viz*.* trachelanthamidine-* N*-oxide (Suppl. Figs. S8 & S9). Fragmentation of the acid motif may generate other characteristic fragment ion signals at *m/z* 254, 255, or 256 through α-splitting and McLafferty rearrangement (Roeder et al. 1991). Usually, the *N*-oxide form of PAs is most common in all the plants’ organs, whereas the lipophilic tertiary state dominates in oily structures viz. seeds (Larcher and Nardin 2019) to be considered in further MS profiling approach using same platform.

### Phenylpropanoid derivatives

Twelve Phenylpropanoid derivatives were annotated herein for the first time in the UPLC-MS spectra of HC extracts, particularly HC-EtOH extract, using negative ionization mode, of which 3 depsides (caffeic acid dimers) and 6 lithospermates (phenylpropanoid oligomers with a 2-phenylbenzofuran moiety) **(31, 34, 38, 39, 41, 57),** in addition to dihydroxyphenyl-lactic acid, caffeic acid, and ethyl caffeate (**3, 11, 40**). Dimers and oligomers of caffeic acid linked together, forming several kinds of polyphenolic skeletons, termed “depsides or salvianolic acids”, are characteristic phytochemicals of the borage and mint families (Omoto et al. 1997). The biosynthesis of these metabolites involves monomeric units of caffeic acid (2–4) molecules with or without danshensu (3,4-dihydroxyphenyl-lactic acid, salvianic acid A) attached via ester links (Jiang et al. 2005; Farag et al. 2016). Three caffeoyl depsides were detected in HC-EtOH and HC-EtOAc viz*.,* rosmarinic acid (**30**), salvianolic acid G (**32**), and salvianolic acid A (**33**). Typically, MS^2^ spectra of these metabolites exhibit fragment ions corresponding to caffeoyl, caffeic acid or danshensu moieties (*m/z* 161, 179, 197 amu, respectively) in negative ionization mode (El-Gazar et al. 2022; Wang et al. 2021). For example, rosmarinic acid (**30**), a caffeic acid ester with danshensu, was ascribed to molecular ion *m/z* 359.0774 [M-H]^−^ (t_R_ = 10.41 min, [C_18_H_15_O_8_]^−^), where the MS^2^ examination revealed a base peak at *m*/*z* 161.02 ([C_9_H_5_O_3_]^−^) corresponding to a caffeoyl moiety after the loss of a danshensu molecule (Suppl. Fig. S10). Similarly, salvianolic acid A (**33**) was assigned to molecular ion *m/z* 493.1140 [M-H]^−^ (t_R_ = 10.55 min, [C_26_H_21_O_10_]^−^) in the three HC extracts, exhibiting a base peak at *m*/*z* 295.06 ([C_17_H_11_O_5_]^−^) and a minor fragment ion at *m*/*z* 313.07 ([C_17_H_13_O_6_]^−^) corresponding to the loss of danshensu and caffeic acid moieties (198 & 180 amu), respectively (Suppl. Fig. S11) (Ożarowski et al. 2017). Salvianolic acid G (**32**), a caffeic acid dimer with an unusual tetracyclic structure, was assigned to the molecular ion at *m*/*z* 341.0665 [M + H]^+^, (t_R_ = 10.50 min, [C_18_H_13_O_7_]^+^) in HC-EtOH extract, reported in *Heliotropium* for the first time in this study. The MS^2^ spectrum of peak **32** revealed fragment ions at *m/z* 323, 313, 297 due to the respective losses of water, carbonyl and carboxylic moieties (Suppl. Fig. S12). Plants of Boraginaceae, namely genus *Lithospermum* harbor another form of caffeate oligomers with a 2-arylbenzofuran moiety termed “lithospermates” (Thuong et al. 2009). Chemically, lithospermic acid is a conjugate of caffeic and rosmarinic acids, which possesses a dihydrobenzofuran nucleus. The MS^2^ spectra of lithospermates usually exhibit fragment ion generated by the neutral losses of a danshensu and/or caffeic acid molecules ([M-H-198]^−^ and [M-H-180]^−^, respectively), in addition to the losses of carboxylic and hydroxyl groups (Hu et al. 2005). In more details, salvianolic acid B (**34**), was assigned to the molecular ion at *m*/*z* 717.1456 [M-H]^−^, (t_R_ = 10.70 min, [C_36_H_29_O_16_]^−^) in HC-EtOAc and HC-EtOH extracts, displaying a base peak at *m*/*z* 519 and a minor fragment ion at *m*/*z* 339, implying successive losses of danshensu and caffeic acid moieties (Suppl. Fig. S13). Methyl (**39**) and dimethyl lithospermate (**41**) showing late elution considering their less polar nature were annotated to molecular ions *m*/*z* 551.1192 and *m*/*z* 565.1349, exhibiting similar base peaks at *m*/*z* 519, in addition to prominent ions at *m*/*z* 321 following the respective loss of danshensu motif (Suppl. Figs. S14 & S15). Salvianolic acid B, lithospermic acid and its esters were reported to increase cell viability, increase the expression of collagen type III and enhance the migration of dermal fibroblasts and keratinocytes (Kim et al. 2012; Szwedowicz et al. 2021).

### Fatty acids, esters and fatty acid amides

Along the second half of the chromatogram of *H. curassavicum* extracts revealed several forms of fatty acids, including 19 fatty acids and 2 fatty acyl esters in negative ionization mode, in addition to 8 fatty acyl amides and 2 fatty acyl esters, detected using positive ionization mode. Such detection is based on improved detection of electronegative elements in fatty acids versus nitrogen-containing lipids in positive ion mode and reflects the value of acquiring in both ion modes. These findings align with the incidence that halophytes are enriched in lipids to accumulate fatty acids as one underlying salt tolerance mechanism (Patel et al. 2019; Elbanna et al. 2023). In this study, several tri- and mono-hydroxy fatty acid conjugates were assigned from their high-resolution masses and tandem MS spectra. Apparently, a mass difference of two mass units between molecular ions indicates a double bond saturation, viz. hydroxy octadecadienoic (**60**), hydroxy octadecenoic (**66**), and hydroxy octadecanoic acids (**72**) having the molecular ion peaks *m/z* 295.2279, 297.2437, and 299.2592 in HC-EtOAc and HC-EtOH extracts, respectively. The lactone of 6-hydroxy hexacosanoic acid (**86**), a long-chain fatty acid ester previously isolated from *H. curassavicum* (Subramanian et al. 1977), and assigned herein to the molecular ion at *m*/*z* 395.3887 [M + H]^+^, (t_R_ = 22.45 min, [C_26_H_51_O_2_]^+^) in HC-EtOH extract (Suppl. Fig. S16). The cleavage pattern of these oxylipids usually shows a base peak generated by the loss of water and a series of low-intensity peaks, separated by 14 mass units, discerning the saturated hydrocarbon chain (Jetter and Riederer 1999). Similarly, 6-docosyloxan-2-one was assigned to molecular ion *m*/*z* 409.4045 [M + H]^+^ in ET extract, (t_R_ = 22.20 min, [C_27_H_53_O_2_]^+^), which is reported herein for the first time in HC (Suppl. Fig. S17). These oxy fatty acid derivatives are of growing interest for their evident anti-inflammatory activities (Kuda et al. 2016).

Fatty acyl amides are another category of metabolites of potential biological merits (Cravatt et al. 1995), and suggestive that nitrogenous compounds are detected not only in alkaloids but likewise oxylipids. The protonated form of oleamide [C_18_H_36_NO]^+^ was assigned to the molecular ion at *m*/*z* 282. 2798 (t_R_ = 16.67 min.) in all examined extracts of *H. curassavicum*. The fragmentation pattern of that class yielded two characteristic product ions: a base peak following the loss of 17 mass units (NH_3_) and a prominent daughter ion following the loss of 18 mass units (H_2_O). Such fragmentation pattern was observed in 4 molecular ions; *m*/*z* 280.2643, 282. 2798, 338.3427 and 366.3739 in *H. curassavicum* extracts, assigned to linoleamide (**63**), oleamide (**68**), docosenamide (**79**) and tetracosenamide (**81**), respectively (Suppl. Fig. S18–S21). Fatty acyl amides, specifically oleamide, are described as signaling molecules with cannabinoid-like pain modulating capacity in humans (De Petrocellis et al. 2000). It’s worth mentioning that this is the first report of these nitrogenous lipids in genus *Heliotropium* although they were isolated from other plant species within the Borage family.

### Biological results

#### Effect of H. curassavicum extracts on wound healing

**Fig. 3:**
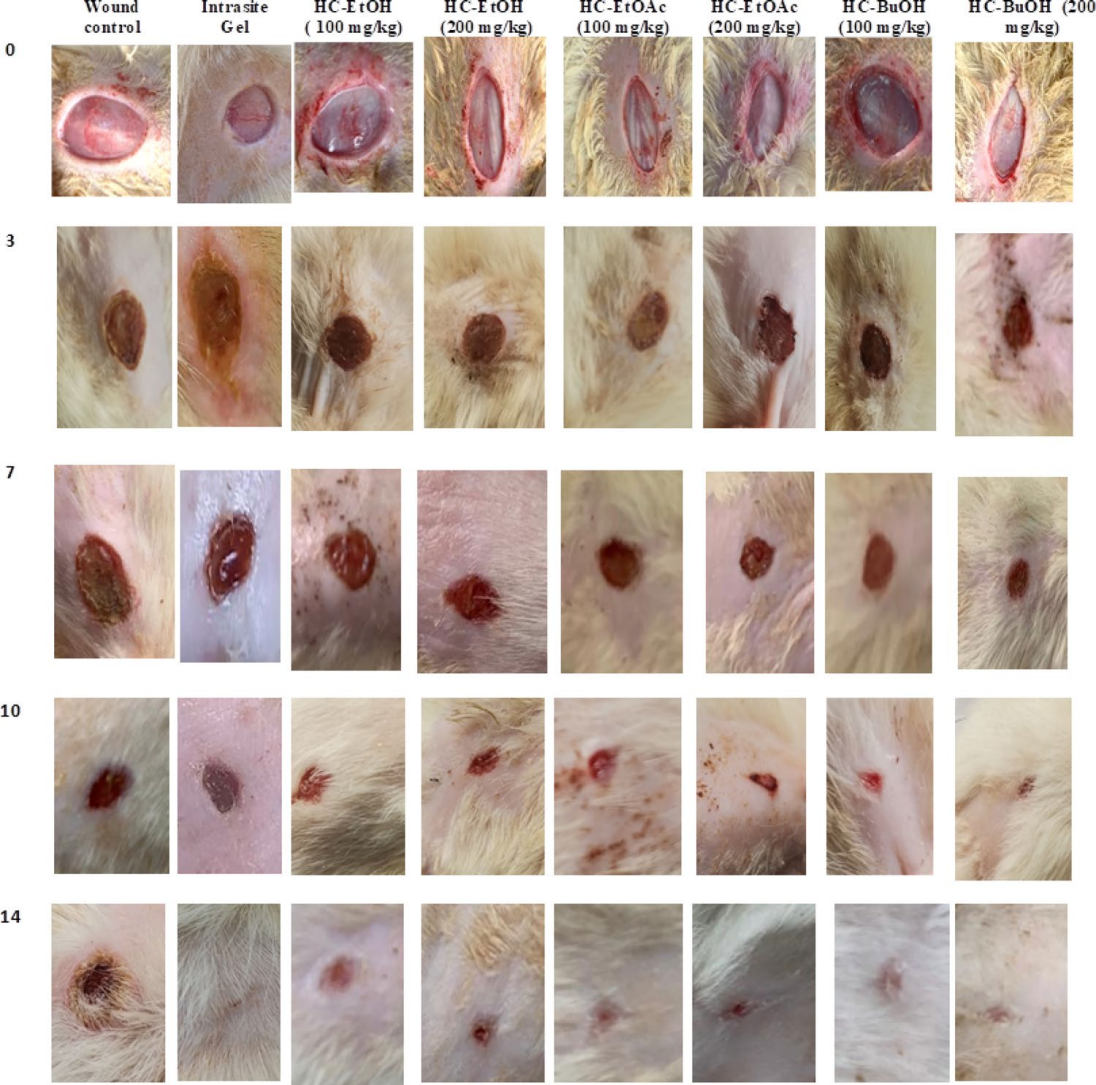
Macroscopic morphology of the healing progress of excision wound model in the control untreated group, different *Heliotropium curassavicum* extracts treated groups at 0-, 3-, 7,10- and 14-days post-wounding

**Fig. 4:**
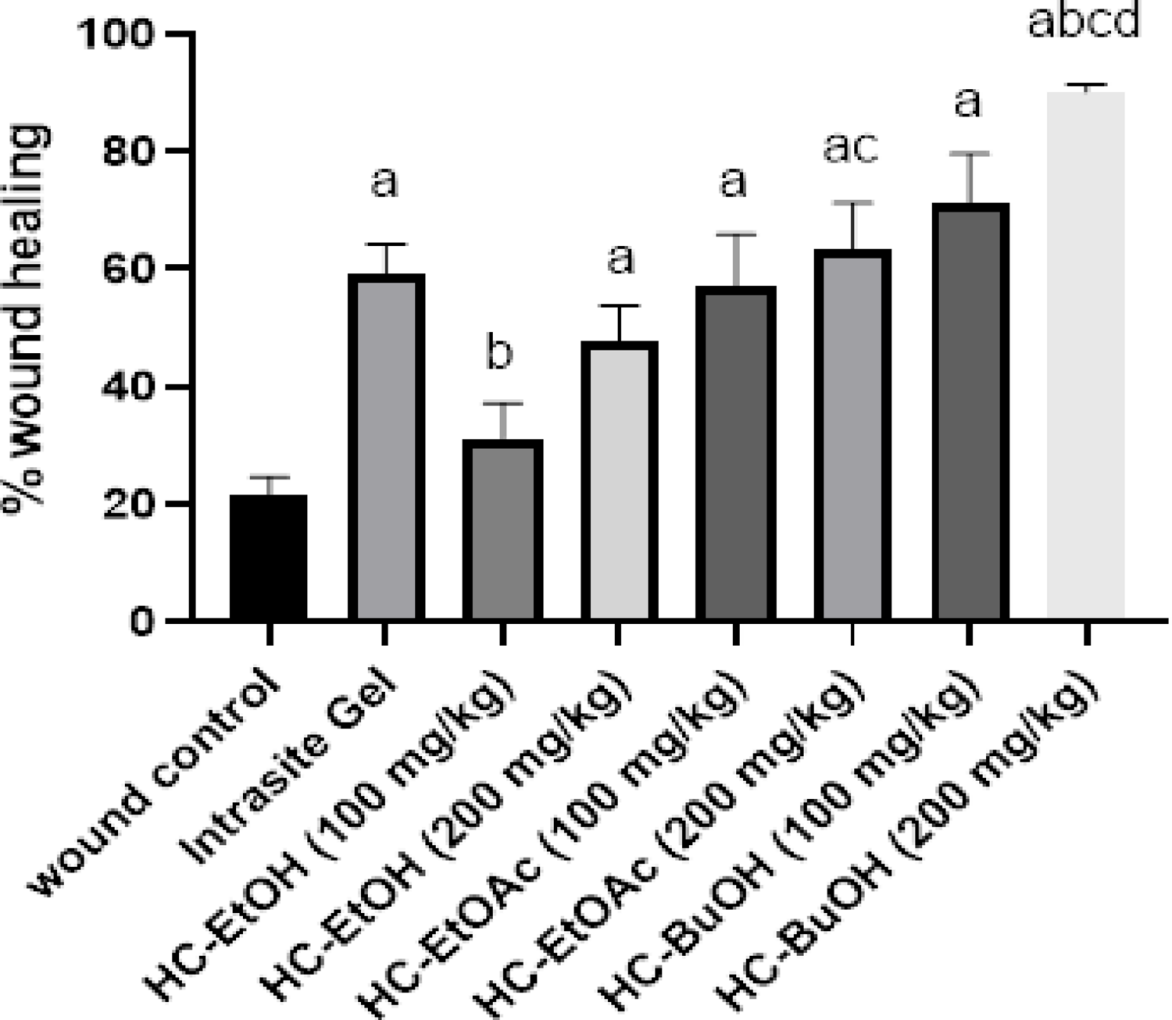
Effect of *Heliotropium curassavicum* extracts on the wound healing %. Data were expressed as mean ± SD. Statistical analysis was carried out by one-way ANOVA followed by Dunnett’s multiple comparisons^a^ Significantly different from wound control. ^b^ Significantly different from HC-EtOH 200 mg/kg. ^c^ Significantly different from HC-EtOAc 200 mg/kg at *p* < 0.05

The images that showed the progression of the wound healing process in the different groups at different time intervals are summarized in Fig. 3. At day 14 post-wounding, nearly complete healing of the wound treated group with the HC-BuOH at high dose was observed, while the wound control group showed a non-healed contracted wound. Moreover, it was observed that, the improvement of the wound healing process after the different *H. curassavicum* extracts is in that order: HC-BuOH > HC-EtOAc > HC-EtOH > Intrasite Gel. This result was confirmed by the wound healing % (Fig. 4). Rats treated with *H. curassavicum* extracts showed a significantly increased healing % when compared to the wound control group. Moreover, the HC-BuOH 200 mg/kg increased wound healing % by 39% when compared to the intrasite gel group.

#### Effect of H. curassavicum extracts on oxidative stress biomarkers

**Fig. 5:**
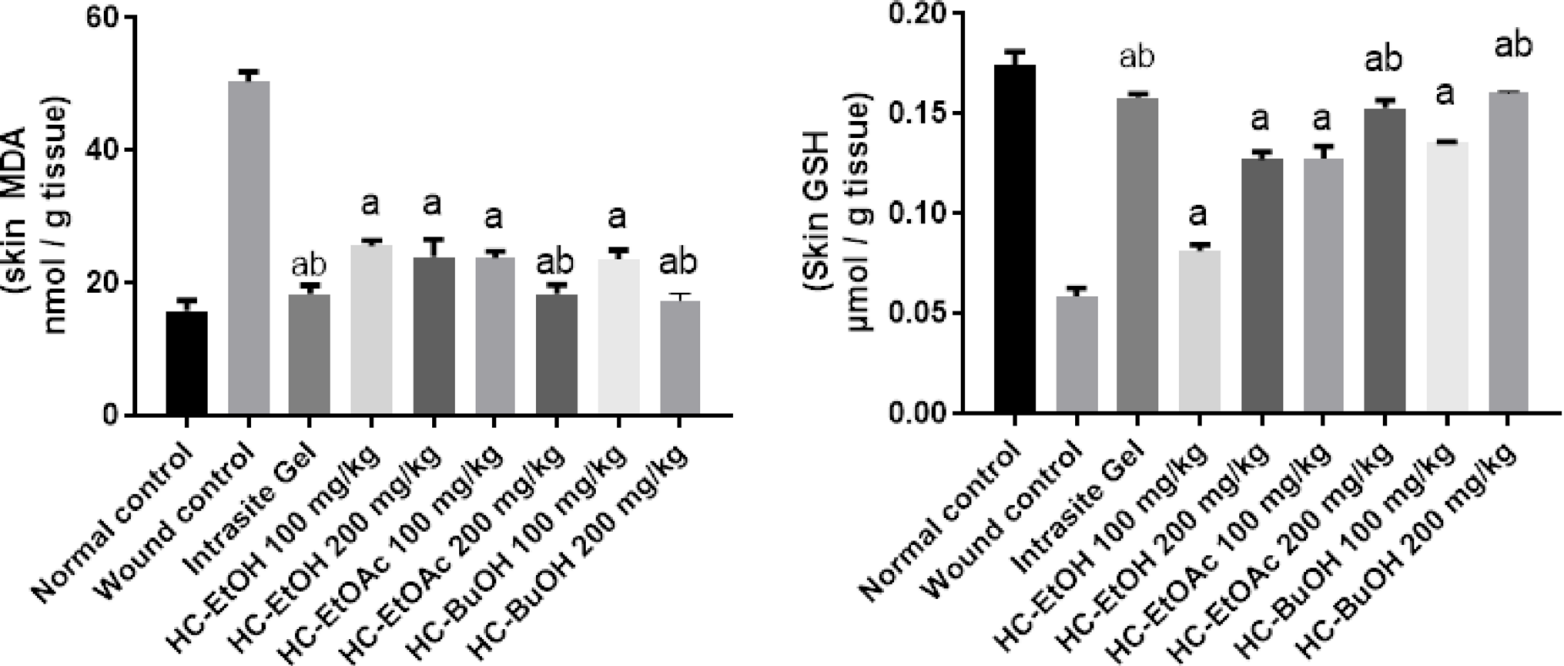
Effect of *Heliotropium curassavicum* extracts on oxidative stress markers. Data were expressed as mean ± SD. Statistical analysis was carried out by one-way ANOVA followed by Dunnett’s multiple comparisons. ^a^ Significantly different from normal control. ^b^ Significantly different from wound control. ^c^ Significantly different from HC-EtOH 200 mg/kg. ^d^ Significantly different from HC-EtOAc 200 mg/kg at *p* < 0.05

Induction of the wound model in rats led to a significant elevation in cutaneous MDA levels compared to the untreated normal controls, indicating enhanced lipid peroxidation. However, topical administration of HC-EtOAc, HC-EtOH, and HC-BuOH extracts at doses of 100 and 200 mg/kg significantly reduced MDA levels by 50%, 54%, 54%, 64%, 54%, and 66%, respectively, relative to the wound control group (*p* < 0.05). Conversely, GSH levels were markedly decreased in the skin of wound-induced rats when compared with normal controls. Treatment with the same extracts at both dose levels significantly restored GSH content, with observed increases of 60%, 140%, 154%, 212%, 160%, and 220%, respectively (*p* < 0.05), demonstrating a dose-dependent antioxidant effect (Fig. 5).

#### Effect of H. curassavicum extracts on inflammatory markers

Exposure to the wound model resulted in a two-fold elevation of TNF-α levels in rat skin tissue relative to the normal control group, indicating a marked inflammatory response. Topical treatment with Intrasite Gel, HC-EtOH, HC-EtOAc, and HC-BuOH extracts at doses of 100 and 200 mg/kg significantly attenuated TNF-α expression by 98%, 37%, 43%, 60%, 74%, 82%, and 94%, respectively, when compared to the untreated wound group (*p* < 0.05). Among these, HC-BuOH demonstrated a notable anti-inflammatory effect, reducing TNF-α levels by 66% relative to the intrasite gel group. At the 200 mg/kg dose, HC-BuOH restored TNF-α concentrations to baseline (normal control) levels.

**Fig. 6:**
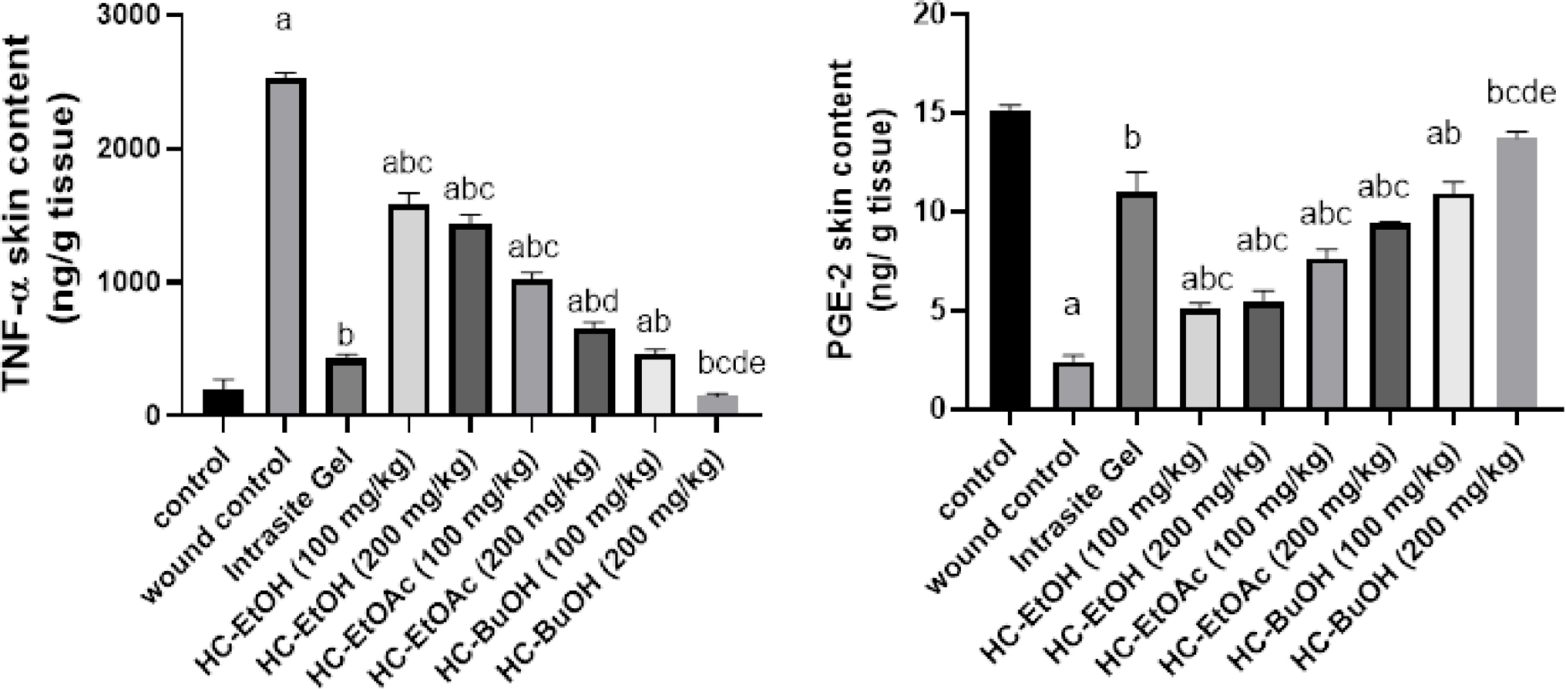
Effect of *Heliotropium curassavicum* extracts on inflammatory markers. Data were expressed as mean ± SD. Statistical analysis was carried out by one-way ANOVA followed by Dunnett’s multiple comparisons. ^a^ Significantly different from n control. ^b^ Significantly different from wound control ^c^ Significantly different from mebo control. ^d^ Significantly different from HC-EtOH 200 mg/kg. ^e^Significantly different from HC-EtOAc 200 mg/kg at *p* < 0.05

In parallel, wound induction led to a substantial suppression of PGE-2 levels, with 85% decrease observed compared to the normal control group. Treatment with the same extracts and intrasite gel significantly elevated PGE-2 levels by 374%, 109%, 125%, 217%, 293%, 356%, and 473%, respectively, relative to the wound control group (*p* < 0.05). HC-BuOH treatment led to a 25% increase in PGE-2 when compared with the Intrasite Gel group. Notably, administration of HC-BuOH at 200 mg/kg normalized (Fig. 6).

#### Histopathological and immunohistopathological results

**Fig. 7:**
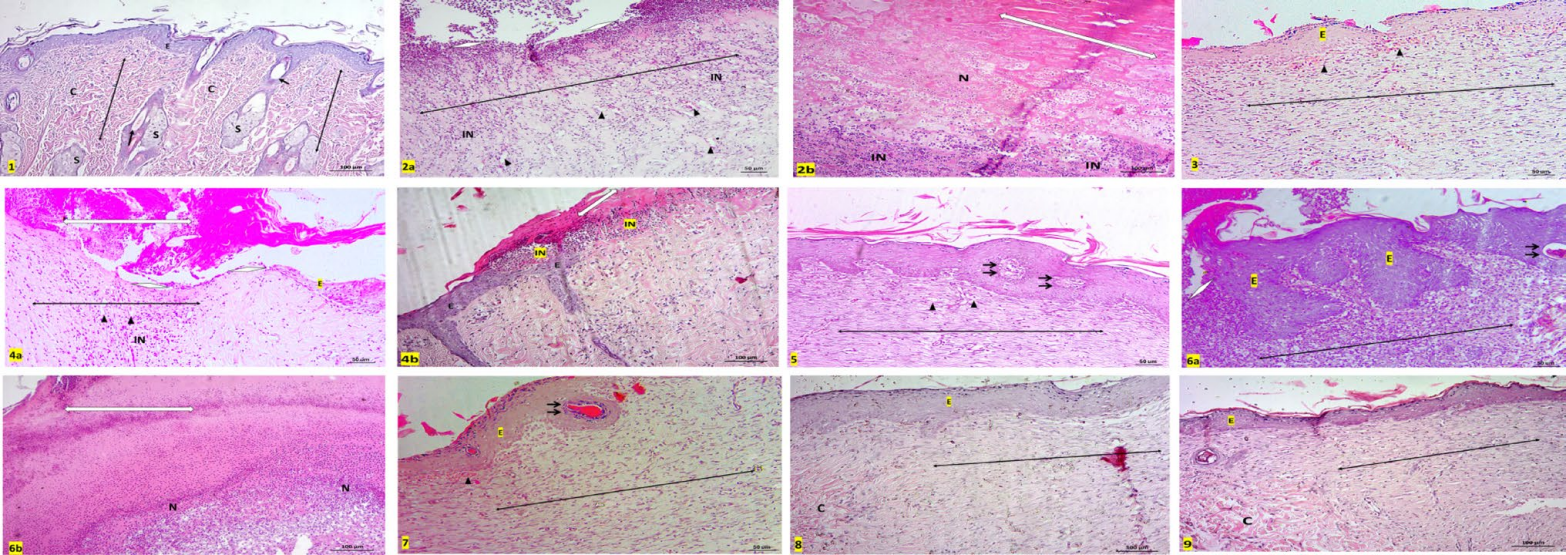
photomicrographs stained with Hx & E (100x), **Group 1 (**negative control) showing normal skin formed of epidermis (E) and dermis (↕) with hair follicles (↑) and sebaceous glands (S). Notice, the dermis of control group contains three-dimensional collagen (C). **Group 2** (wound control) showing areas with lost epidermis and failure of epithelialization (◊) after 14 days (**2a,2b)**. Some areas are necrosed (N), filled with inflammatory cells (IN) and covered with large crust or scab (⇔) (**2c)**, Thickened epidermis (E) at injury margin is seen in **2b**. The dermis is mostly replaced by granulation tissue (↕) with mononuclear inflammatory cells (IN) and neovascularization (∆) with absence of skin appendages (**2a,2b)**. Notice the difference between normal three-dimensional collagen (C) and granulation tissue at injury site (↕) (**2b)**. **Group 3** reference drug (Intrasite Gel) showing epidermis (E) covering injury site, new vascularization (∆), granulation tissue with parallel collagen fibers and still absence of skin appendages in the dermis (↕). **Group 4** (treated by 100mg/kg of HC-EtOH) showing failure of epithelialization (◊) with necrotic tissue and scab (⇔) covering the center (**4a,4b**). Thin Epidermal edge (E) is seen at the wound margin (**4a**)**.** Creeping of inflammatory cells (IN) from the center of the wound toward the normal epidermis (E) at the edge is also noticed in **4b.** Dermis is still showing granulation tissue (↕) with mononuclear inflammatory cells (IN), neovascularization (▲) at injury site (**4a,4b). Group 5** (treated by 200mg/kg of HC-EtOH) showing thick epidermis (E) with hyperplasia and keratin pearls (↑↑) at injury site with no skin appendages and apparent parallel collagen fibers in the dermis (↕). **Group 6** (treated by100mg/kg of EtOAc) showing thicker epidermis (E) at the edge only (**6a)** while the center of the wound showed lost epidermis that replaced by large crust (⇔) and necrosis (N) **(6b**). Dermis still showing granulation tissue (↕) and inflammatory cells **(6a). Group 7** (treated by 200mg/kg of EtOAc) showing apparent thicker epidermis with hyperplasia and keratin pearls (↑↑) at injury site and dermis with no skin appendages ( ↔). **Groups 8, 9** (treated by HC-BuOH100&200mg/kg respectively) showing epidermis (E) covering injury site and apparent least inflammatory infiltration as compared to other groups in dermis ( ↔), notice difference between normal three-dimensional collagen (C) and collagen at injury site ( ↔). The effect of treatment by different types of *Heliotropium curassavicum* extracts on mean epidermal thickness

**Fig. 8:**
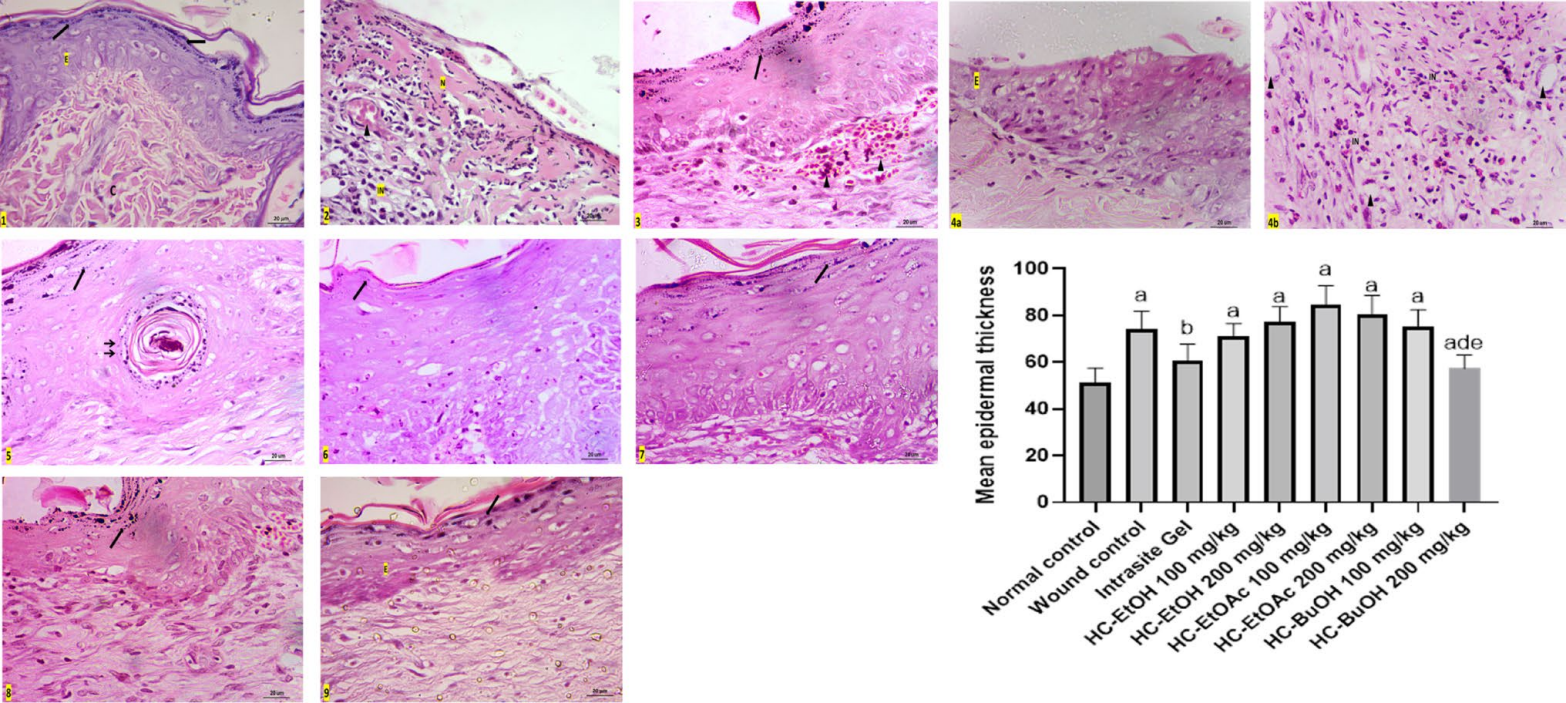
higher magnification (400x) showing normal epidermis (E) with basophilic keratohyalin granules ($\uparrow$) in granular cell layer and acidophilic collagen bundles in reticular dermis (C) in **group 1**(control group). **Group 2** (wound group) showing lost epidermis with necrotic areas (N), congested blood capillaries (▲) and inflammatory cells (IN) in dermis. **Group (3) (**Reference drug) showing epidermis with clear stratum granulosum and basophilic keratohyalin granules ($\uparrow$) and congested blood capillaries in dermis (▲). **Group 4** showing also dilated blood capillaries (▲) and many inflammatory cells (IN) in dermis **(4a,4b)** but no granular cell layer is seen in the epidermal edge (E) (**4a**). Epithelialization with hyperplasia and keratin pearls (↑↑) is seen with clear keratohyalin granules in **group 5. Group 6** showing increased epidermal thickness at the edge but with no keratohyaline granules. **Groups 7,8, 9** showing epithelialization at injury site with clear keratohyalin granules ($\uparrow$) but **group 7** showing thicker epidermis as compared to **8,9.** The dermis appeared with no inflammatory cells infiltrate **(8,9**)

Epithelialization is a fundamental phase of wound healing involving the proliferation and migration of keratinocytes to restore epidermal integrity. In this study, re-epithelialization remained incomplete in the wounded rats (Group 2), as well as in Groups 4 and 6 (treated with 100 mg/kg of HC-EtOH and HC-EtOAc, respectively), as evidenced by the presence of necrotic tissue and thick crusts even after 14 days. Despite the failure of re-epithelialization in these groups, epidermal thickening was observed at the wound margins (Figs. 7 and 8). Histological analysis revealed the presence of mononuclear inflammatory cells within granulation tissue across several groups, particularly in the wounded rats (Group 2), and groups 4 and 6 (treated with 100 mg/kg of HC-EtOH and HC-EtOAc, respectively). Neovascularization was evident in many treated groups, suggesting active tissue repair. However, in Groups 2, 4, and 6, the persistent presence of granulation tissue and inflammatory cells after 14 days implied delayed healing (Figs. 7 and 8). In contrast, groups 5 and 7 (treated with 200 mg/kg of HC-EtOH and HC-EtOAc, respectively) demonstrated noticeably thicker epidermal layers with keratin pearls covering the injury site. Groups 8 and 9 (treated with 100 and 200 mg/kg of HC-BuOH, respectively) showed immunopositive Ki-67 cells confined to the basal cell layer of the epidermis and dermis, with Group 9 (treated with HC-BuOH 200 mg/kg) displaying a less intense positive reaction in the dermis, suggesting a more localized response to treatment. Meanwhile, the wounded rats (Group 2) exhibited widespread Ki-67–positive cells in the dermis, indicating ongoing inflammation and active cell proliferation. Groups 5 and 7 also showed increased immunopositive proliferative cells in both the epidermis and dermis, accompanied by notable epidermal thickening (Fig. 7 and 8).

**Fig. 9:**
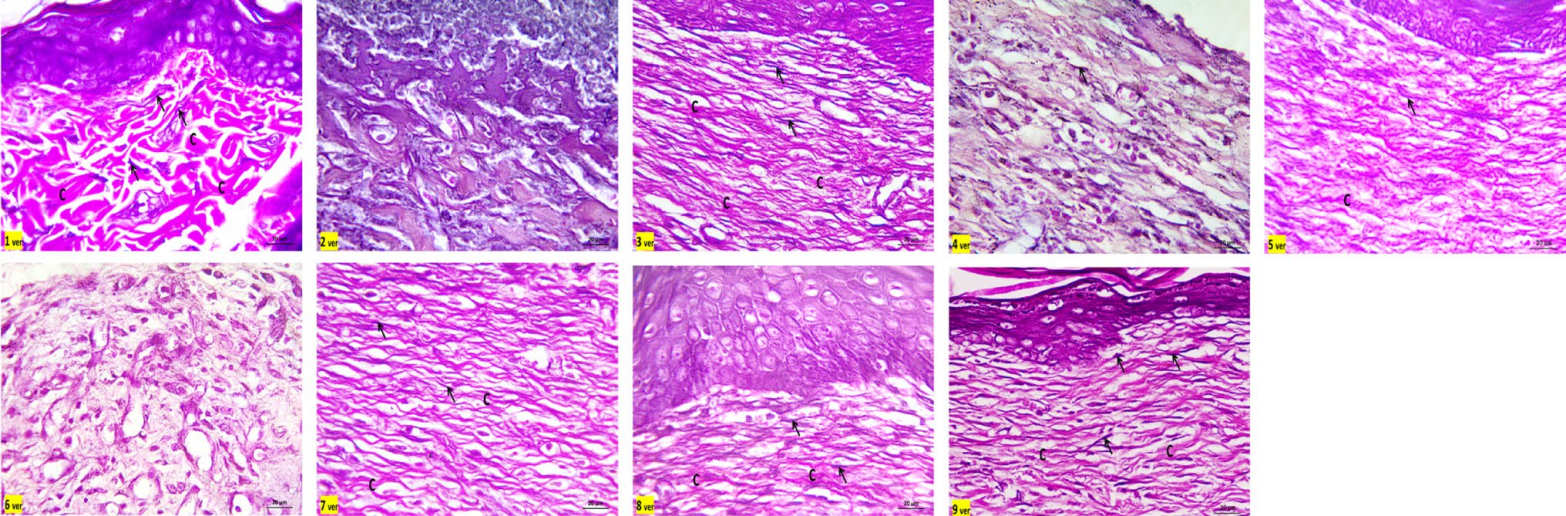
Stained sections with Verhoeff stain (400x), it stains thick collagen fibers red (C) and thin elastic fibers dark blue (↑). Notice clear discrepancy between both fibers in **control group** and **group 3** (Intrasite Gel). Both fibers were seen clearly in **groups 8,9** (treated by 200mg/kg HC-BuOH extract). Other **groups 2, 4, 6** and **7** showed no discrepancy between the stained disorganized fibers in dermis. **Group 5** showed some discrepancy between the fibers in dermis

The Verhoeff staining further highlighted variations in dermal remodeling. The control group exhibited distinct thick collagen fibers and thin elastic fibers, reflecting a healthy dermal matrix. In contrast, Groups 2, 4, and 6 displayed disorganized and poorly differentiated fibers. Groups 5 and 7 (treated with 200 mg/kg of HC-EtOH and HC-EtOAc, respectively) exhibited partially aligned red collagen bundles, while Group 8 (treated with HC-BuOH 100 mg/kg) showed improved fiber orientation. Notably, Group 9 (treated by HC-BuOH 200 mg/kg) demonstrated the most organized collagen and elastic fiber pattern among all experimental groups (Fig. 9).

**Fig. 10:**
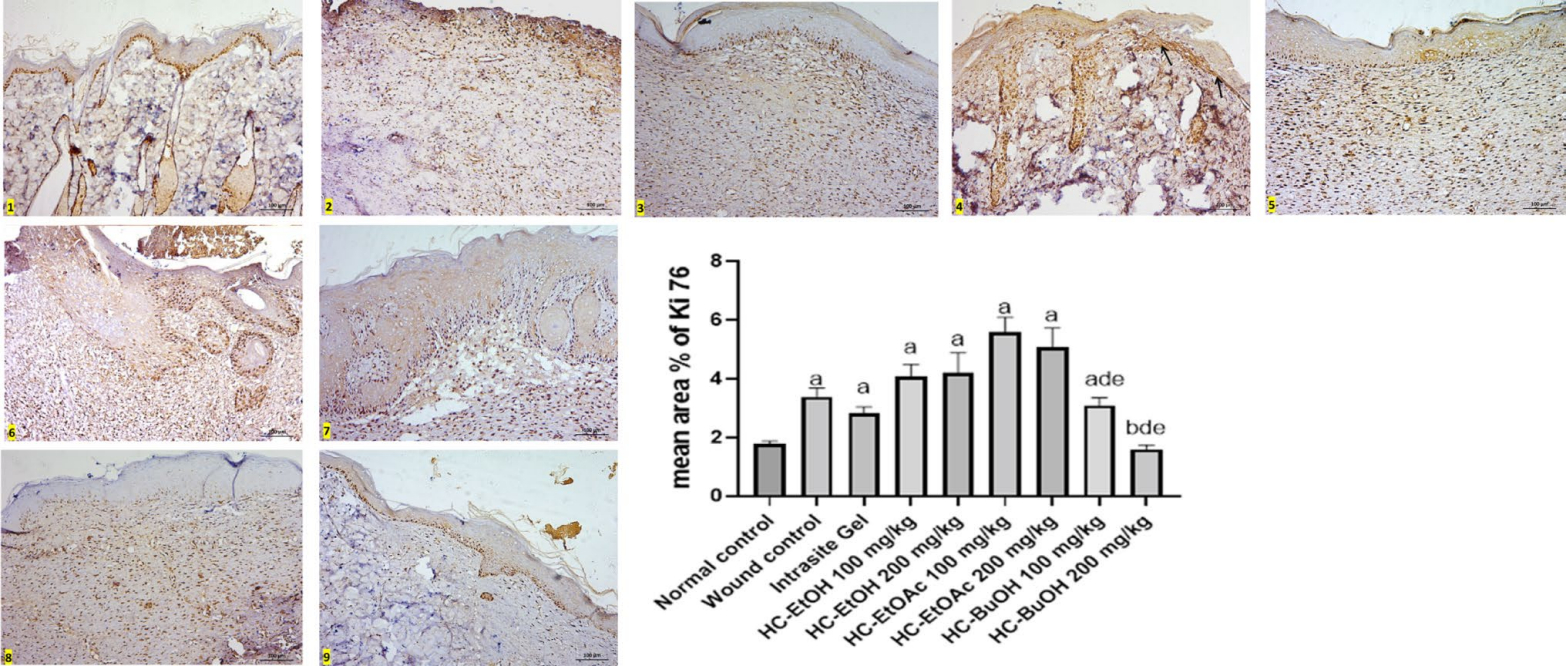
Photomicrographs stained with Ki 67 (100x), **Group 1** (control group) showing normal immune positive basal cell layer of epidermis and hair follicles with no apparent immunopositive cells in dermis. **Group 2** (wound control) showing many immunopositive cells in dermis with lost epidermis was at injury site. **Group 3** reference drug (intrasite Gel) showing immunopositive cells confined to basal layers of the epidermis and some cells in dermis. **Groups 4,5** (treated by 100mg/kg&200mg/kg of HC-EtOH) showing increased immunopositive proliferative cells in epidermis and dermis at injury site in group **(5)** and at epidermal edge only with notable positive reaction in some cells at injury site creeping to epidermal edge (↑) in group **(4)**. **Groups 6, 7** (treated by 100&200mg/kg of HC-EtOAc) also showing increased number of immunopositive cells at epidermal edge next to injury and all through the basal layer of epidermis at injury site in groups 6 and 7 respectively and in dermis with increased thickness of epidermis. Dermis also showing many immunopositive cells. **Groups 8,9** (treated by 100&200mg/kg of HC-BuOH) showing immunopositive cells confined to basal cell layer of epidermis. Many immunopositive cells was seen in dermis of group 8, but it was less in group 9

Immunohistochemical analysis using Ki-67 staining revealed varying patterns of cellular proliferation across the experimental groups. In the untreated control group (Group 1), Ki-67 immunopositive cells were restricted to the basal layer of the epidermis and hair follicles, with no detectable staining in the dermis, indicating normal proliferative activity. In contrast, the wounded rats group (Group 2) demonstrated abundant Ki-67–positive cells in the dermis, accompanied by a complete loss of the epidermis at the injury site, reflecting heightened inflammation and disrupted epithelial integrity. The reference drug-treated group (Group 3) showed a more organized pattern, with immunopositive cells primarily confined to the basal layers of the epidermis and scattered cells in the dermis. Groups treated with HC-EtOH (Groups 4 and 5) exhibited dose-dependent responses, where Group 5 (200 mg/kg) displayed a marked increase in Ki-67–positive cells throughout the epidermis and dermis at the injury site, while Group 4 (100 mg/kg) showed immunopositive cells predominantly at the epidermal edge and to a lesser extent at the injury site. Similarly, Groups 6 and 7 (treated with 100 and 200 mg/kg of HC-EtOAc, respectively) demonstrated increased Ki-67 expression, with immunopositive cells observed along the entire basal layer of the epidermis at the injury site and in the dermis, along with noticeable epidermal thickening. In contrast, Groups 8 and 9 (treated with 100 and 200 mg/kg of HC-BuOH, respectively) showed a more localized pattern of Ki-67 expression, confined to the basal layer of the epidermis. While Group 8 exhibited numerous positive cells in the dermis, their number was notably reduced in Group 9, suggesting a more regulated and confined proliferative response at the higher dose (Fig. 10).

#### Synergistic interactions result

**Fig. 11:**
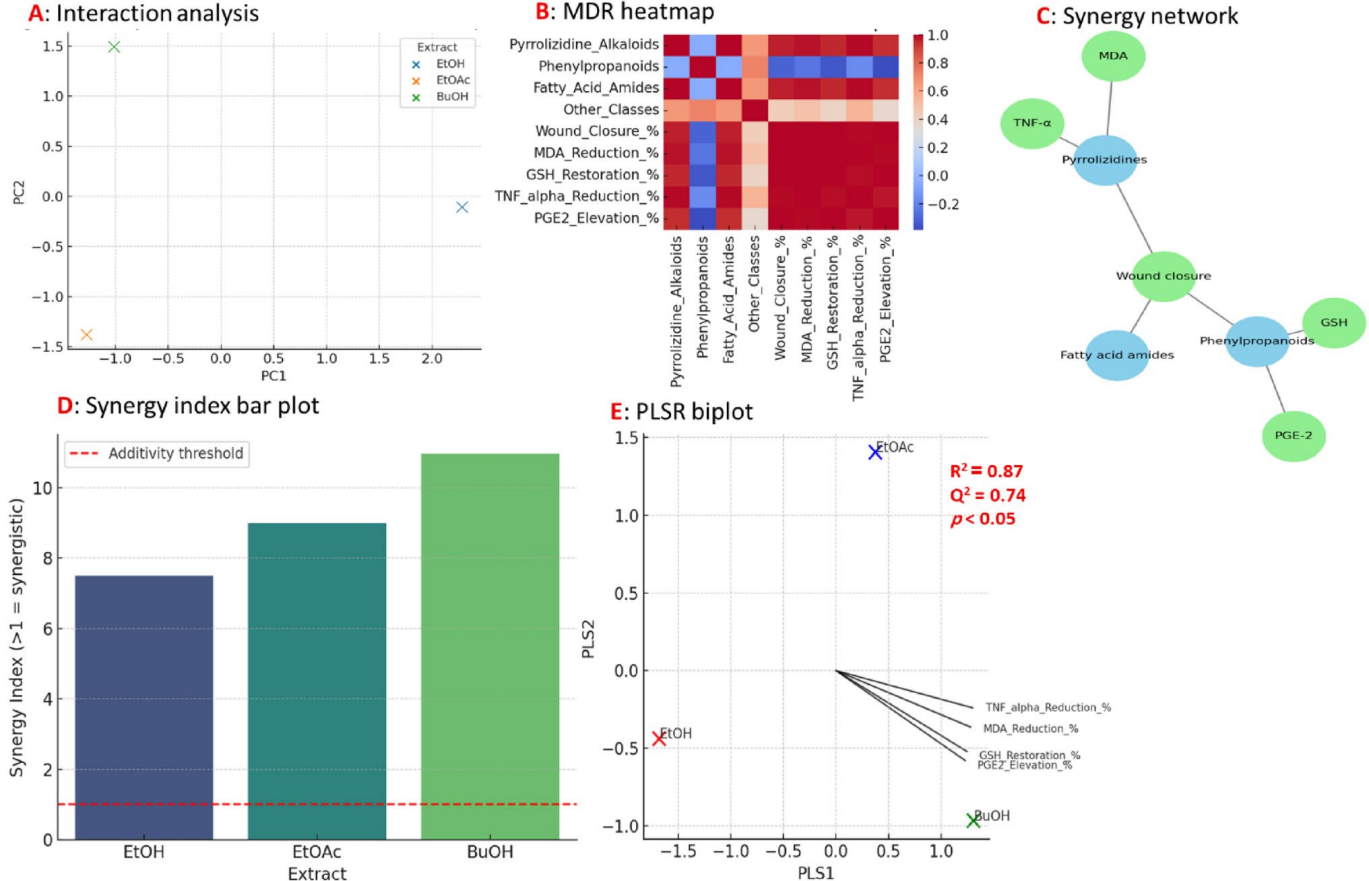
Statistical demonstration of metabolite synergy in *Heliotropium curassavicum* wound healing. **A** Interaction analysis showing that wound closure improves most strongly when pyrrolizidine alkaloids and phenylpropanoids occur together at higher levels, indicating synergistic effects. **B** Multifactor dimensionality reduction (MDR) heatmap illustrating that combined metabolite classes predict healing outcomes more accurately than individual classes, with pyrrolizidines and phenylpropanoids contributing most strongly. **C** Synergy network depicting significant associations between metabolite classes and biological markers. Pyrrolizidines and phenylpropanoids emerge as central drivers, strongly linked to oxidative stress reduction (MDA), anti-inflammatory effects (TNF-α suppression, PGE-2 elevation), and antioxidant restoration (GSH). **D** Interaction plot (synergy index) demonstrating that combinations of pyrrolizidine alkaloids + phenylpropanoids and pyrrolizidine alkaloids + fatty acid amides are significantly more effective than predicted by additivity. **E** Partial least squares regression (PLSR) biplot integrating metabolite profiles with biological outcomes. Pyrrolizidines and phenylpropanoids align positively with protective factors (GSH, PGE-2) and inversely with detrimental markers (MDA, TNF-α), supporting a coordinated synergistic mechanism (R^2^ = 0.87; Q^2^ = 0.74; *p* < 0.05)

The multi-panel synergy figure provides an integrated view of the complex interactions among metabolite classes in *H. curassavicum* extracts and their contribution to wound healing efficacy. The interaction plot (Fig. 11A) demonstrates that wound closure is not related to the abundance of a single metabolite class, but rather depends on the concurrent presence of pyrrolizidine alkaloids and phenylpropanoids, with healing outcomes being markedly superior when both are elevated, suggesting a genuine synergistic effect. This relationship was further supported by the multifactor dimensionality reduction analysis (Fig. 11B), where the predictive accuracy of wound healing is highest when these metabolite classes are considered together, exceeding the explanatory power of each class, and confirming that the biological effect is driven by interactions rather than isolated contributions. The synergy network (Fig. 11C and D) expands this perspective by mapping the interconnections between metabolites and healing biomarkers, showing that pyrrolizidines and phenylpropanoids are central nodes that strongly influence oxidative stress and inflammatory mediators, while fatty acid amides act as supportive contributors, reinforcing the cooperative framework of metabolite action. Finally, PLS regression biplot (Fig. 11E) integrates the metabolite dataset with the measured biological responses, demonstrating a clear pattern in which metabolite scores cluster alongside beneficial biomarkers such as GSH and PGE-2, while exhibiting an inverse relationship with detrimental markers including MDA and TNF-α. This distribution, supported by strong model performance (R^2^ = 0.87; Q^2^ = 0.74; and *p* < 0.05), highlights the metabolic basis for the observed histological improvements. Collectively, the panels indicate that the enhanced wound-healing activity of the HC-BuOH extract does not stem from any single dominant metabolite class. Instead, it is derived from synergy among pyrrolizidine alkaloids, phenylpropanoids, and fatty acid amides, which together modulate oxidative stress and inflammatory pathways to drive accelerated and more effective tissue regeneration.

## Discussion

Skin has the capacity to repair damaged or lost tissues by regenerating tissues and forming a collagenous scar. This process is called “wound healing” and consists of coagulation, inflammation, proliferation, and remodeling phases (Osman and Amin 2020). A macroscopic image shows that the wound healing progression in the *H. curassavicum* different extracts and the wounded group between day 0 and day 14. On day 10, there was a significant difference between the wound areas of the treated groups and the wounded control group. On day 14, the skin regeneration in the HC-BuOH was almost completed, while the healing of the wounded control group was still in progress (Fig. 2), suggesting that the tested extract enhanced wound healing at the macroscopic scale. Skin damage, either burns or excisional, is closely linked to metabolic changes brought on by oxidative processes, which generate free radicals through shared biological pathways. Variability in free radical generation after using antioxidants seems to be important for treating skin injuries (Kuffler 2016). This study showed that mechanical skin damage led to elevated blood proinflammatory indicators and oxidative stress. In addition, compared to the wound control group, the current study demonstrated that different types of extract of *H. curassavicum* can mitigate the consequences of mechanical injury by significantly lowering skin MDA levels and raising GSH and antioxidant enzyme activities. These findings are consistent with recent research showing elevated lipid peroxidation within two weeks of burn injuries and reduced antioxidant enzyme activity linked to oxidative stress, microcirculation disruption, and antioxidant system depletion (Jere and Houreld 2022). Furthermore, it has been shown that activated leukocytes release free radicals, which exacerbate inflammation and lipid peroxidation (Mosca et al. 2019).

ROS plays a key role in many stages of the healing process and is intimately associated with it (Pang et al. 2024). In conclusion, maintaining a balance between the benefits and drawbacks of ROS and minimizing the harm brought on by oxidative stress are crucial for wound healing (Huang et al. 2022). In fact, skin injury increased ROS formation in the inflammatory and regeneration phases, as well as oxidative damage markers (lipid peroxidation) until the 7th day. Overproduction of ROS causes significant oxidative damage, including lipid peroxidation, and cell death by changing the potential of the mitochondrial membrane (Raziyeva et al. 2021).

Among the wound healing processes, the proliferation stage includes re-epithelialization, granulation tissue formation, and angiogenesis. If one or more stages of the wound healing process are interrupted, the healing rate will be delayed (Takeuchi 2014). PGE is widely distributed in vivo, among which prostaglandin E2 (PGE2) exhibits an important regulatory role in physiological processes, such as inflammatory and anti-inflammatory responses. Prostaglandins have been shown to have many effects on wound healing. PGE2 potentiates the inflammatory response by increasing the effects of other mediators. PGE2 also stimulates vasodilation and promotes the development of edema. This net result is to increase capillary permeability to facilitate the extravasation of serum proteins (cytokines, chemokines, growth factors) into the wound, which helps orchestrate the healing process (Pang et al. 2024). The results of the present study showed that *H. curassavicum* extracts could prominently reduce the expression of PGE2, reducing local inflammatory reactions and inhibiting fibrosis at the inflammatory reaction stage.

While the inflammatory response during the first phase of cutaneous wound healing is crucial to protect the organism from infection and further harm, it is also a major factor in the pathogenesis of scar formation (Yang et al. 2024). During the healing process, the colonization of bacteria increases tissue inflammation and delays healing time, which results in increased scar production. The inflammatory stage is the initial line of defense against bacteria. In this period, colonization of the bacteria in the wound tissue induces inflammation. Pro-inflammatory cytokines, for instance, TNF-α and IL-1β, are extensively produced in response to the infection (Guo et al. 2024). Herein, the topical applications of *H. curassavicum* extracts as ointment-based formulation were observed within significantly decreased TNF-α content on the 14th day after the wound induction in all treated groups compared to wounded control group and return to its normal level in the HC-BuOH applied rats. Thus, this observed rapid wound healing with HC-BuOH extract could be attributed to these properties.

The observed incomplete epithelialization in the wounded rats (Group 2), and rats treated with 100 mg/kg of HC-EtOH and HC-EtOAc (groups 4 and 6, respectively) could be attributed to the presence of necrotic tissue and thick crusts, which likely acted as physical barriers, impeding keratinocyte migration and thereby delaying wound closure. This impaired healing may be the result of persistent inflammation, potentially driven by an imbalance between pro- and anti-inflammatory cytokines and excessive ROS production. These factors can induce apoptosis in keratinocytes and fibroblasts, as previously described (Gushiken et al. 2021). The presence of epidermal thickening at the wound margins, despite the failure of full re-epithelialization, may be associated with the aberrant activation of the β-catenin/c-myc signaling pathway, known to promote keratinocyte hyperproliferation in chronic wounds (Häkkinen et al. 2004). Dysregulated expression of β6-integrin, which has been linked to defective epidermal repair in chronic human wounds, further, supports this notion (Stojadinovic et al. 2005).

The presence of mononuclear inflammatory cells and neovascularization in granulation tissue confirms progression into the proliferative phase of healing. Macrophages, through the secretion of growth factors such as VEGF and TGF-β, play pivotal roles in angiogenesis and fibroblast activation (Eming et al. 2007). Enhanced vascularization observed in certain treated groups further indicates active tissue regeneration. However, the persistent inflammation seen in the wounded rats (Group 2), and rats treated with 100 mg/kg of HC-EtOH and HC-EtOAc (groups 4 and 6, respectively) suggests a disrupted healing process. This aligns with findings from Qian et al. (33) and Holzer-Geissler, who emphasized that unresolved inflammation and elevated levels of inflammatory mediators such as IL-6 and MMP1 hinder proper wound resolution and remodeling (Qian et al. 2016; Holzer-Geissler et al. 2022).

The Verhoeff stain results supported these observations. While the control group displayed normal dermal architecture, the wounded rats (Group 2), and rats treated with 100 mg/kg of HC-EtOH and HC-EtOAc (groups 4 and 6, respectively) showed disrupted collagen and elastic fiber organization, reflecting impaired tissue remodeling. The more structured fiber arrangement observed in Groups 5 and 7 (treated with 200 mg/kg of HC-EtOH and HC-EtOAc, respectively) indicates some degree of remodeling, although still characteristic of scar tissue. The superior fiber alignment in Group 9 (treated by HC-BuOH 200 mg/kg), however, suggests effective extracellular matrix regeneration, possibly resulting from the modulating effects of HC-BuOH on fibroblast activity and collagen deposition. Altogether, the data suggest that while HC-EtOH and HC-EtOAc at higher doses may enhance epithelial proliferation, their effects on tissue organization and inflammation contribute to abnormal remodeling. In contrast, HC-BuOH appears to support more regulated wound healing with favorable histological and molecular markers of tissue regeneration.

Groups 5 and 7 (treated with 200 mg/kg of HC-EtOH and HC-EtOAc, respectively) showed enhanced epidermal thickening and keratin pearl formation, paralleling findings by Farci and Gauri (Farci and Mahabal 2020), inferred that hyperplasia and keratin pearl development are markers of impaired keratinocyte function in chronic wounds. While keratinocyte proliferation is essential for wound healing, hyperproliferation and abnormal turnover—as suggested by increased Ki-67 immunopositivity—can result in pathological scar formation, including hypertrophic and keloid scars (Limandjaja et al. 2017). The increased epidermal thickness and cell proliferation observed in Groups 5 and 7 (treated with 200 mg/kg of HC-EtOH and HC-EtOAc, respectively) suggest that while HC-EtOH and HC-EtOAc at higher doses may stimulate keratinocyte activity, they may also promote aberrant wound remodeling. In contrast, the controlled Ki-67 expression in Groups 8 and 9 (treated by HC-BuOH 100 and 200 mg/kg, respectively) indicates a more balanced proliferative response, potentially leading to improved healing outcomes with reduced risk of excessive scarring (Piipponen et al. 2020).

Current findings indicate intriguing avenues for additional mechanistic studies that are consistent with established processes of plant-derived bioactive components in wound healing. In accordance with earlier findings that plant phenolics strengthen antioxidant defenses in fibroblasts, including activation of Nrf2 and suppression of matrix-degrading enzymes under oxidative challenge, the noted restoration of GSH and reduction of MDA demonstrate modulation of oxidative stress (Merecz-Sadowska et al. 2021). The anti-inflammatory effects of phenolic acids like caffeic acid, which in animal wound models increased collagen-like polymer synthesis, inhibited lipid peroxidation, and decreased pro-inflammatory mediator release, are mirrored by the decrease in the inflammatory marker TNF-α and the normalization of PGE-2 (Song et al. 2008). Improved collagen/elastic fiber organization and increased epidermal proliferation (Ki-67 expression) indicate impacts on cell migration, proliferation, and extracellular matrix remodeling—important stages in cutaneous repair. The mechanisms were attributed to the ability of these phytochemicals to coordinate antioxidant defense, inflammation resolution, ECM deposition, and cell proliferation throughout the inflammatory, proliferative, and remodeling stages (Riaz et al. 2024). The observed proliferative, anti-inflammatory, and antioxidant effects of *H. curassavicum* extracts point to possible modulation of important wound-healing pathways, such as Nrf2-mediated antioxidant defense, NF-κB and COX-2-mediated inflammatory signaling, and extracellular matrix remodeling because of the wound-healing activity of phenolics, pyrrolizidine alkaloids, and fatty acid amides (Riaz et al. 2024).

The observed superior wound healing activity of the *H. curassavicum* extracts, especially the HC-BuOH, might be closely linked to the synergistic effect of several key bioactive metabolite classes, as identified by LC-MS/MS profiling. These include pyrrolizidine alkaloids (PAs), phenylpropanoid derivatives, and fatty acid conjugates and amides, each contributing to distinct but complementary biological mechanisms essential for tissue repair (El-Kashak et al. 2025; Chrzanowska et al. 2024). PAs—especially trachelanthamidine-based and *N*-oxide derivatives i.e., curassavine and lycopsamine-*N*-oxide—were prevalent across all extracts but were particularly abundant in HC-BuOH. These alkaloids possess known anti-inflammatory properties through their modulation of inflammatory mediators such as TNF-α and PGE-2 (Zan et al. 2023; Moreira et al. 2018; Chauhan et al. 2022). Their ability to downregulate pro-inflammatory cytokines is consistent with the substantial reduction in TNF-α (up to 94%) observed by the best active extract, HC-BuOH, than other evaluated extracts. Additionally, specific *N*-oxide forms of PAs are more water-soluble, which could facilitate their bioavailability and activity in the wound microenvironment (Mattocks and Bird 1983).

Phenolics like rosmarinic acid, salvianolic acids A and B, and lithospermic acid esters—detected predominantly in the EtOAc and BuOH extracts—are potent antioxidants (Guan et al. 2022; Piątczak et al. 2024). Their presence likely underpins the pronounced oxidative stress reduction (66% MDA reduction; 220% GSH increase) in HC-BuOH-treated wounds. Salvianolic acid B, in particular, has been reported to stimulate collagen synthesis and fibroblast proliferation, aligning with the histological findings of enhanced collagen remodeling and re-epithelialization in the HC-BuOH group (Meng et al. 2022).

The HC-BuOH extract was also rich in hydroxylated fatty acids and fatty acyl amides like oleamide and docosenamide. These lipids are not only structural components but also serve as signaling molecules with roles in inflammation resolution and tissue regeneration (Wasserman et al. 2020; Jara et al. 2020). Oleamide, for example, mimics endocannabinoid activity, contributing to pain modulation and inflammatory control (Pillarisetti et al. 2009). The presence of long-chain hydroxy fatty acids further suggests barrier-restoring and moisturizing effects essential for optimal healing (Cardoso et al. 2011).

The butanol extract’s polarity likely allowed optimal extraction of a broader range of bioactive constituents, particularly amphipathic molecules like *N*-oxide alkaloids and fatty acyl amides. This could explain the superior bioactivity of HC-BuOH, as it offers a more pharmacologically complete profile compared to the more polar (EtOH) or non-polar (EtOAc) counterparts. The enhanced wound healing effects of the HC-BuOH extract can be attributed to the integrated actions of its diverse bioactive constituents. Pyrrolizidine alkaloids provided anti-inflammatory activity, phenylpropanoids offered strong antioxidant and pro-collagen effects (Le et al. 2015; Korkina et al. 2011; Neelam and Sharma 2020), and fatty acid derivatives contributed to both structural repair and signaling (Jara et al. 2020).

However, most evidence is derived from crude extracts of many plants, and it is still unknown how each PA specifically contributes. There is a lack of complete safety profiles for repeated application, percutaneous penetration, and long-term topical usage. Despite these drawbacks, the available results show that topically applied PA-rich extracts can promote wound healing with no systemic danger as long as formulations are regulated for exposure and concentration (Cramer and Beuerle 2012).

Despite their well-known systemic hepatotoxicity, PAs have demonstrated encouraging topical wound-healing efficacy in mice. In rat excision and incision models, extracts from *H. indicum* and *H. bacciferum* improved granulation tissue formation, accelerated wound closure, improved epithelialization, increased collagen deposition (hydroxyproline content), increased antioxidant enzyme activity (SOD, catalase, GSH), and decreased oxidative stress (Reddy et al. 2002; Dash and Murthy 2011, Fathalipour-Rayeni et al. 2022). Bioactive fractions from *H. indicum* supported tissue regeneration by promoting fibroblast and keratinocyte migration, as further demonstrated using in vitro scratch (Sarkar et al. 2021, Yao et al. 2011). Crucially, there was no discernible systemic harm from topical treatment in these investigations, indicating less percutaneous absorption than oral exposure.

Several new findings about *H. curassavicum* are presented in this study mostly exemplified by many pyrrolizidine alkaloids not previously reported in the genus *Heliotropium*. Further evidence that the wound-healing effect results from a synergistic interaction between phenylpropanoids, fatty acid amides, and pyrrolizidine alkaloids rather than from any one metabolite class comes from multivariate modeling. These findings provide a molecular insight into how the extract’s wound-healing properties are driven by a variety of metabolite groups. This phytochemical synergy likely accounts for the superior biochemical, histological, and clinical outcomes observed in case of HC-BuOH-treated group, establishing it as a promising candidate for wound healing therapy.

This study key limitation is the use of healthy rats as the experimental animal model, which represents normal physiological wound healing rather than compromised or chronic healing situations. The intricacy of therapeutically relevant circumstances, such as diabetic, immunocompromised, or otherwise degraded wound settings, is not adequately captured by this model, even if it is suitable for determining baseline wound-healing activity. Future research using impaired wound-healing models will be required to confirm the extract’s therapeutic value and more precisely assess each metabolite group’s relative effectiveness in pathological settings. Additionally, to determine the exact molecular mechanisms underlying the observed wound-healing effects, future mechanistic investigations, including in vitro studies of fibroblast and keratinocyte proliferation and migration, measurements of antioxidant enzyme activity, examination of matrix metalloproteinases and collagen expression, and fractionation-based evaluation of metabolite synergy will be essential.

## Conclusion

This study highlights the potent wound healing efficacy of *H. curassavicum*, particularly its *n*-butanol extract (HC-BuOH), which demonstrated superior therapeutic outcomes compared with both conventional treatment and other solvent extracts. UHPLC-ESI-MS/MS profiling uncovered a diverse metabolite repertoire—including newly reported pyrrolizidine alkaloids such as uplandicine and uluganine—underscoring the extract’s rich phytochemical complexity. Biologically, HC-BuOH accelerated wound closure and promoted complete re-epithelialization within 14 days, supported by enhanced fibroblast proliferation and structured collagen deposition. Its therapeutic action was mechanistically linked to a pronounced reduction in oxidative stress and inflammation, as evidenced by substantial modulation of MDA, GSH, TNF-α, and PGE-2 levels. Histopathological observations further confirmed organized tissue regeneration and regulated keratinocyte proliferation. The findings point to a synergistic mechanism mediated by the interplay of pyrrolizidine alkaloids, phenylpropanoids, and fatty acid amides, positioning HC-BuOH as a robust candidate for phytopharmaceutical development in wound care. This work lays a strong foundation for future mechanistic studies and clinical translation, advancing the therapeutic potential of *H. curassavicum* in dermatological applications.

## Supplementary Information

Below is the link to the electronic supplementary material.

Supplementary Material 1

## Data Availability

Data will be made available on request.
