# Supplementary material for: Synergistic wound healing mechanisms of Heliotropium curassavicum extracts via redox modulation, inflammation suppression, and tissue remodeling: linking phytochemical diversity to antioxidant and anti-inflammatory effects
Source: Inflammopharmacology. 2026 Feb 16;34(3):1659–82. doi: 10.1007/s10787-025-02096-z (PMC12996408; doi:10.1007/s10787-025-02096-z)
Supplement: Supplementary file 1 — Supplementary Material 1 [file 10787_2025_2096_MOESM1_ESM.pptx]

## Slide 1
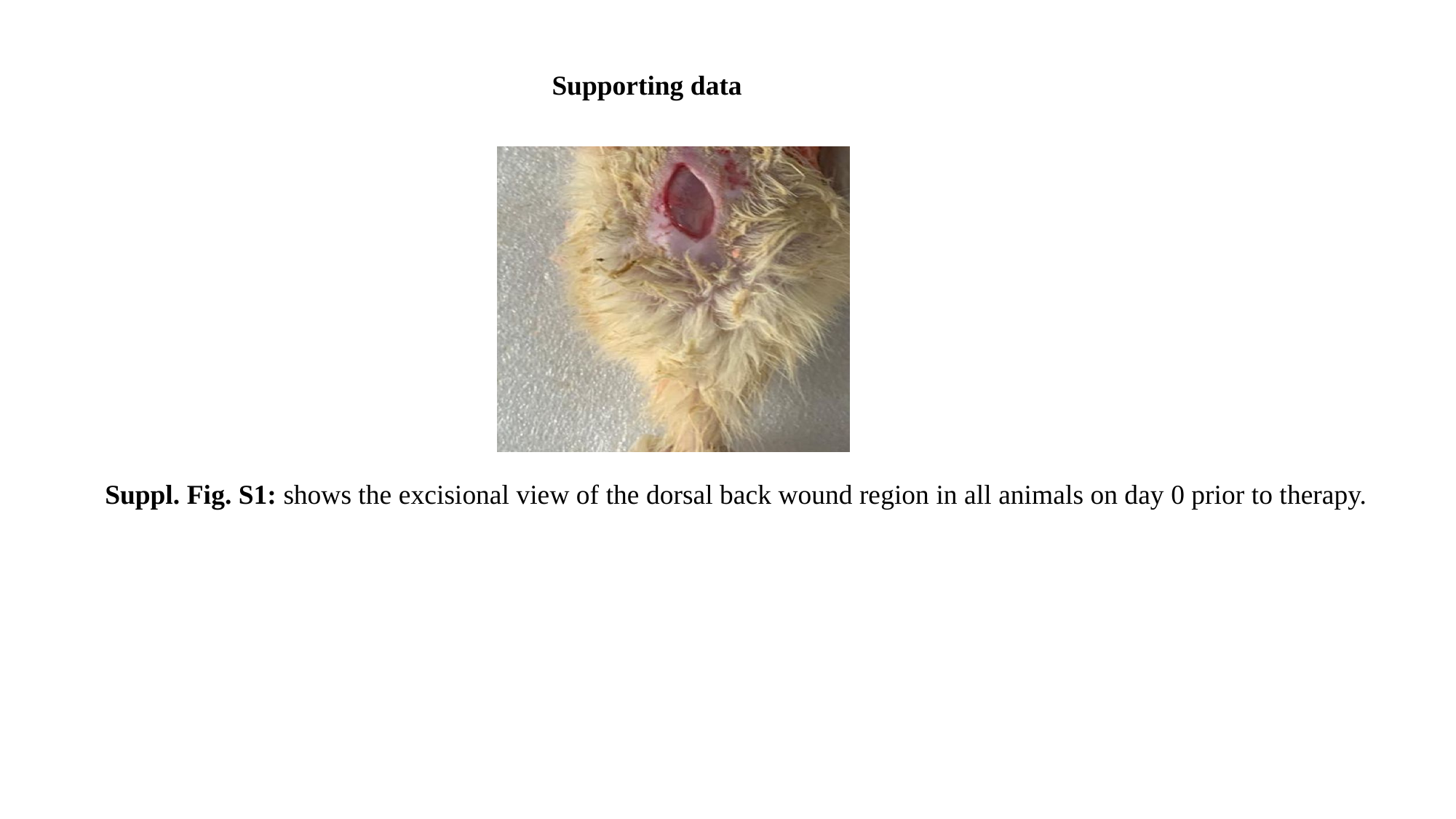

Supporting data
Suppl. Fig. S1: shows the excisional view of the dorsal back wound region in all animals on day 0 prior to therapy.

## Slide 2
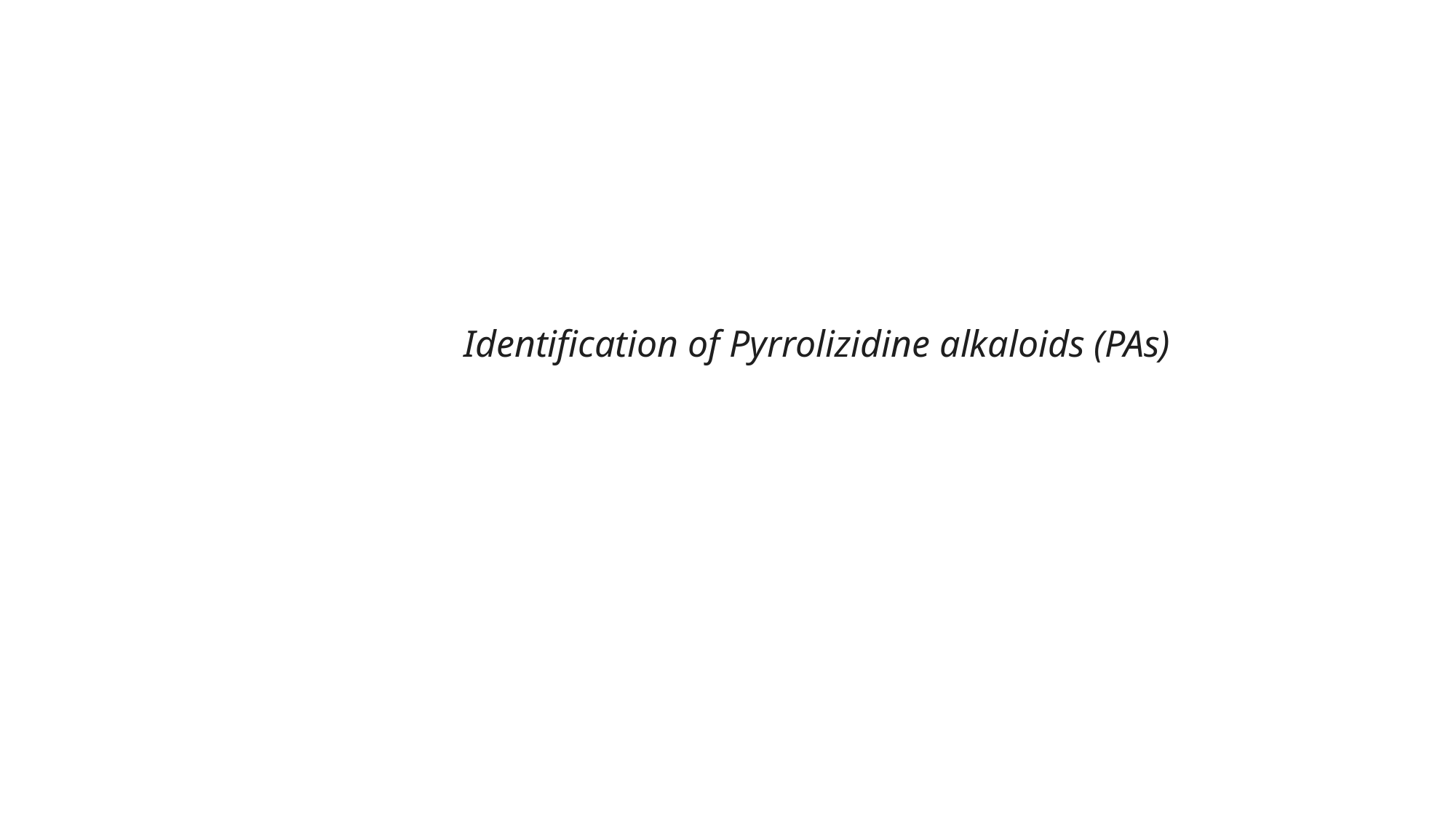

Identification of Pyrrolizidine alkaloids (PAs)

## Slide 3
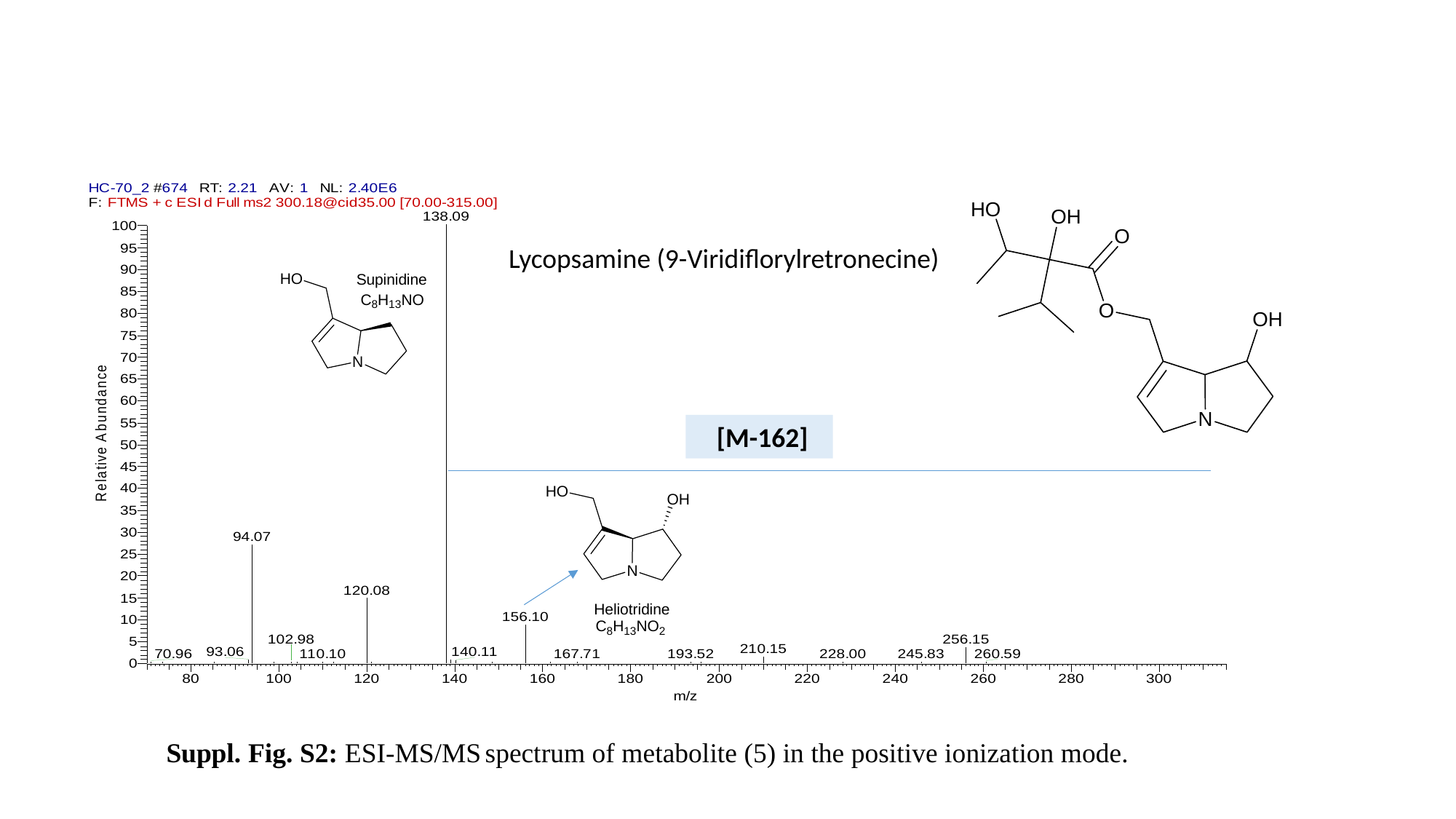

Lycopsamine (9-Viridiflorylretronecine)
 [M-162]
Suppl. Fig. S2: ESI-MS/MS spectrum of metabolite (5) in the positive ionization mode.

## Slide 4
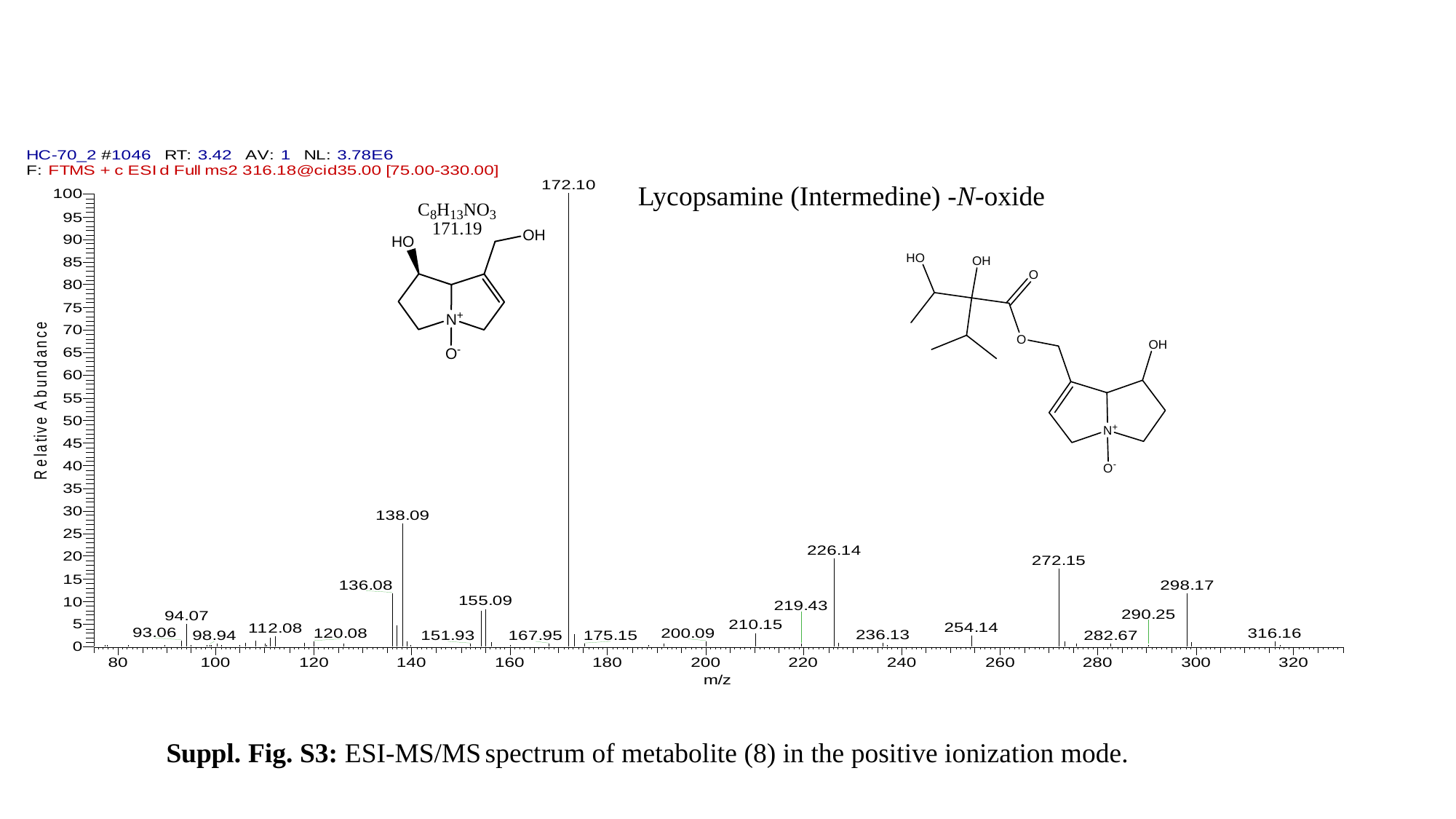

Lycopsamine (Intermedine) -N-oxide
Suppl. Fig. S3: ESI-MS/MS spectrum of metabolite (8) in the positive ionization mode.

## Slide 5
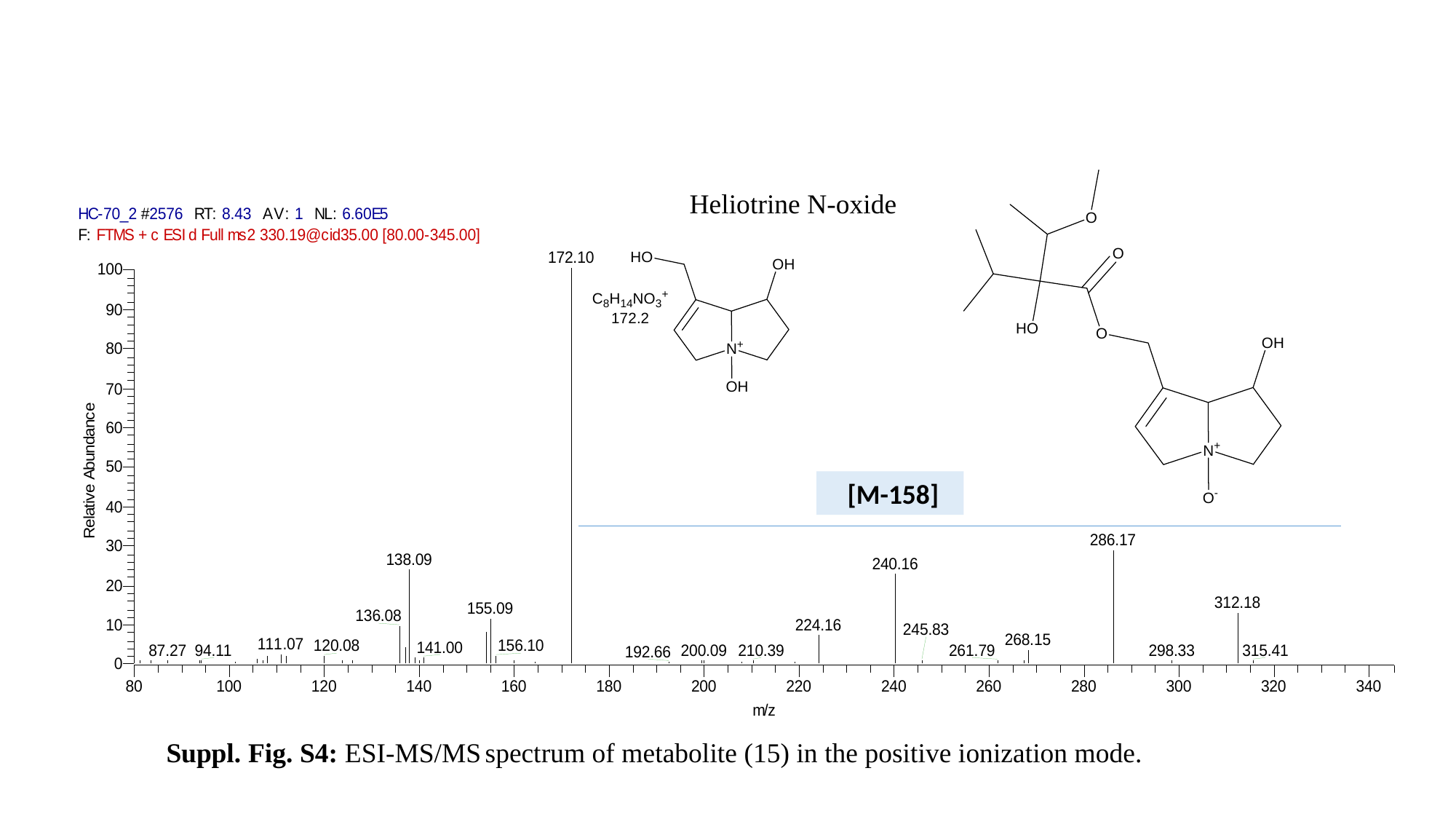

Heliotrine N-oxide
 [M-158]
Suppl. Fig. S4: ESI-MS/MS spectrum of metabolite (15) in the positive ionization mode.

## Slide 6
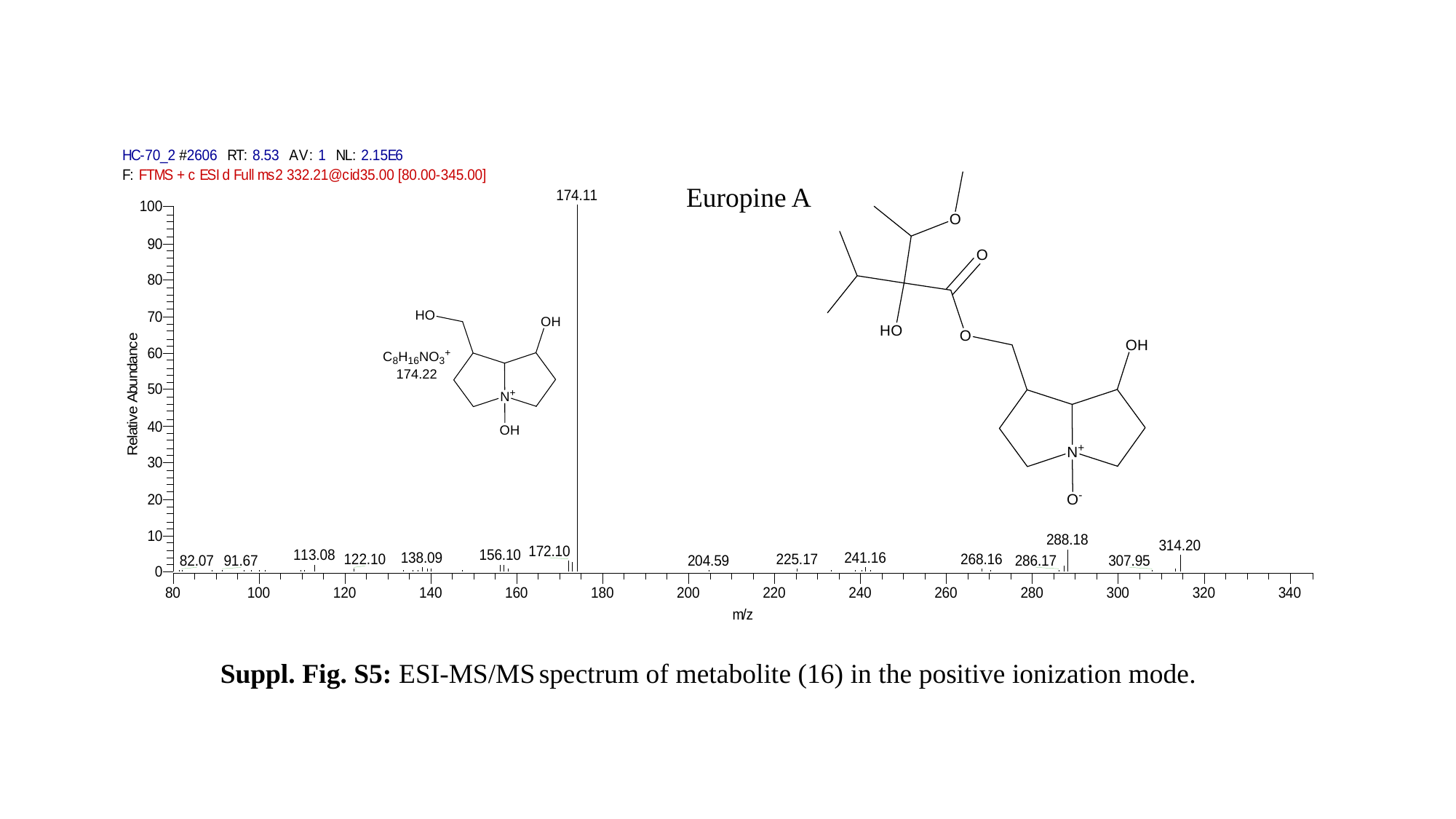

Europine A
Suppl. Fig. S5: ESI-MS/MS spectrum of metabolite (16) in the positive ionization mode.

## Slide 7
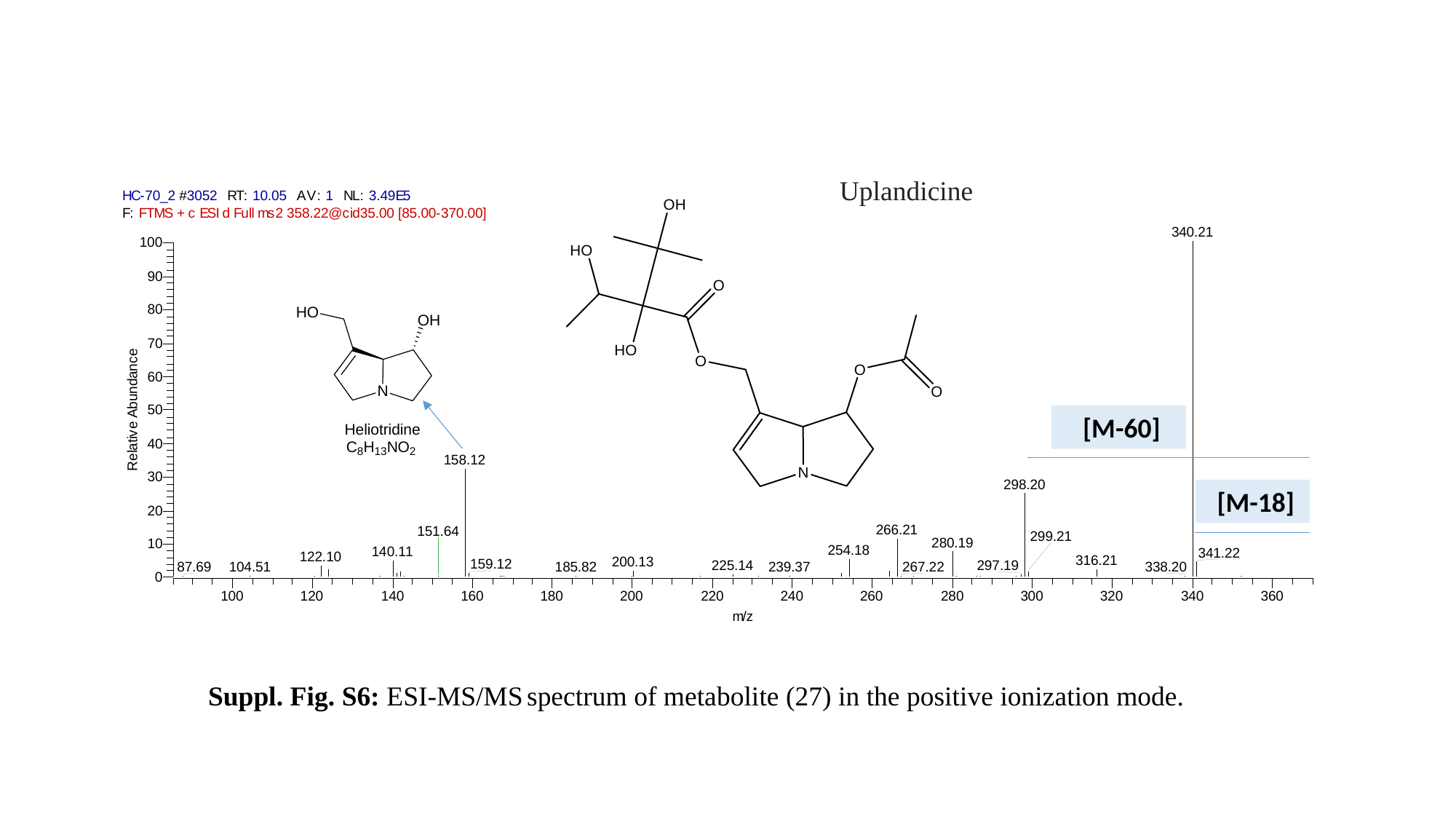

Uplandicine
 [M-60]
 [M-18]
Suppl. Fig. S6: ESI-MS/MS spectrum of metabolite (27) in the positive ionization mode.

## Slide 8
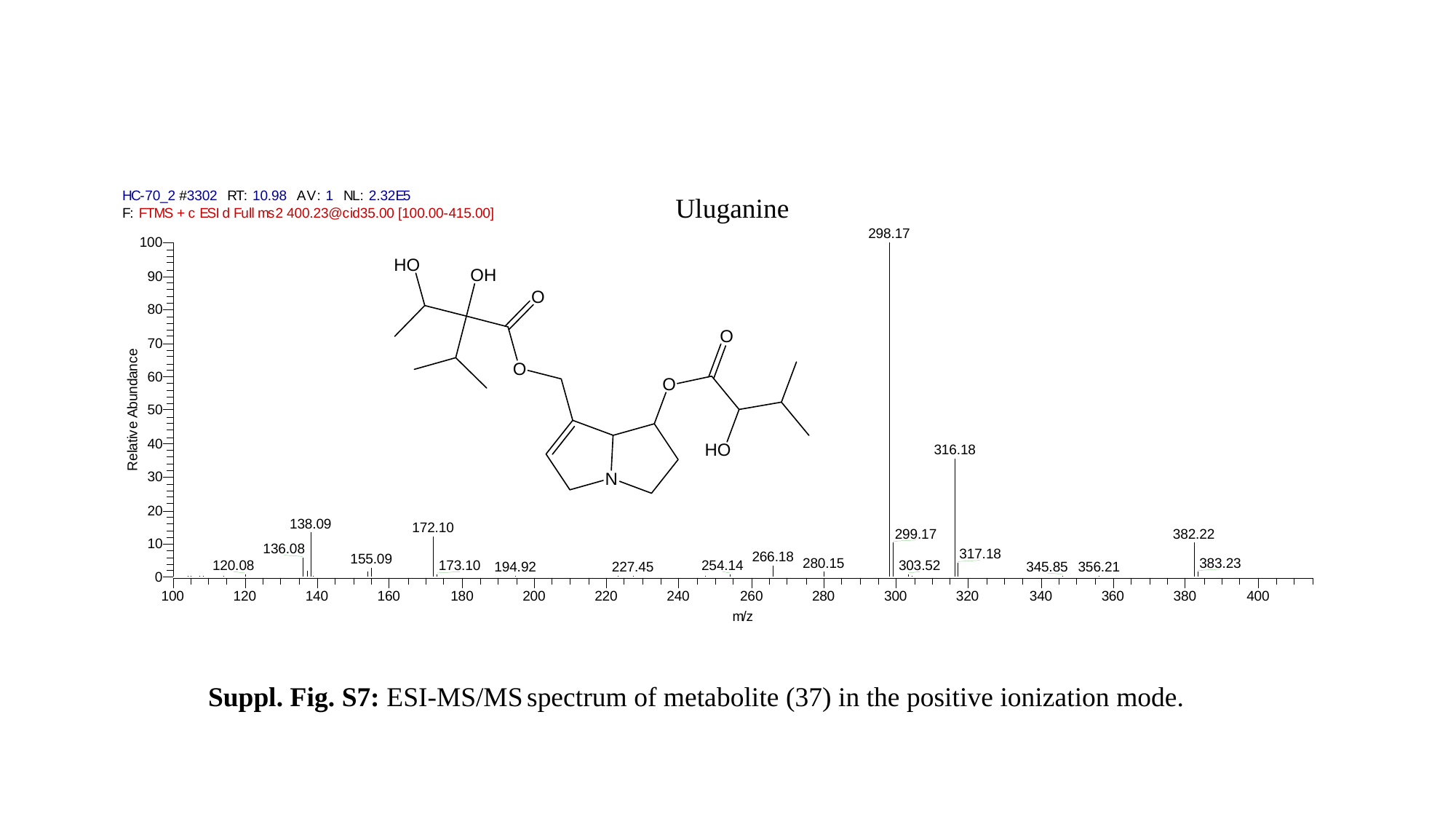

Uluganine
Suppl. Fig. S7: ESI-MS/MS spectrum of metabolite (37) in the positive ionization mode.

## Slide 9
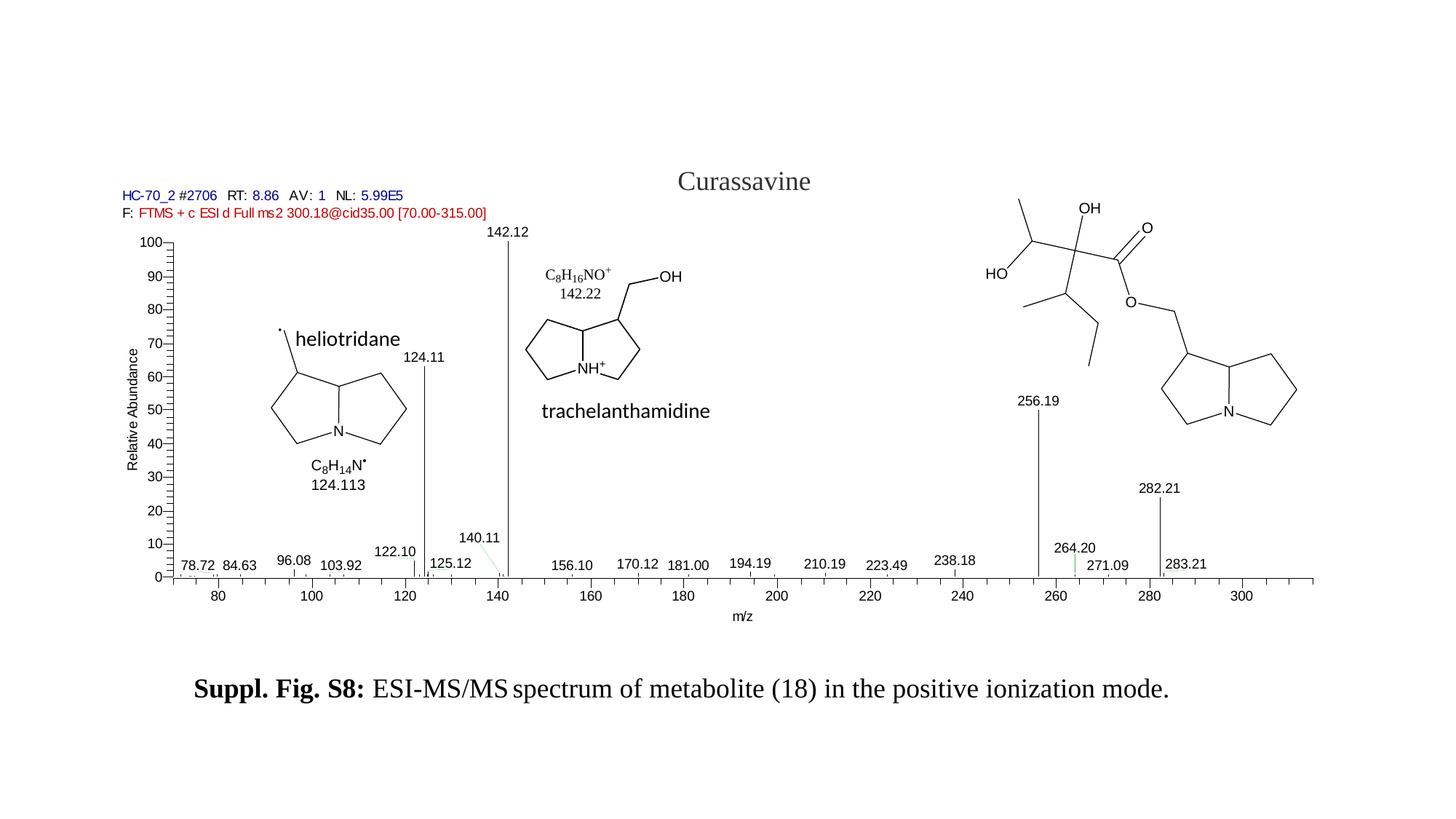

Curassavine
heliotridane
trachelanthamidine
Suppl. Fig. S8: ESI-MS/MS spectrum of metabolite (18) in the positive ionization mode.

## Slide 10
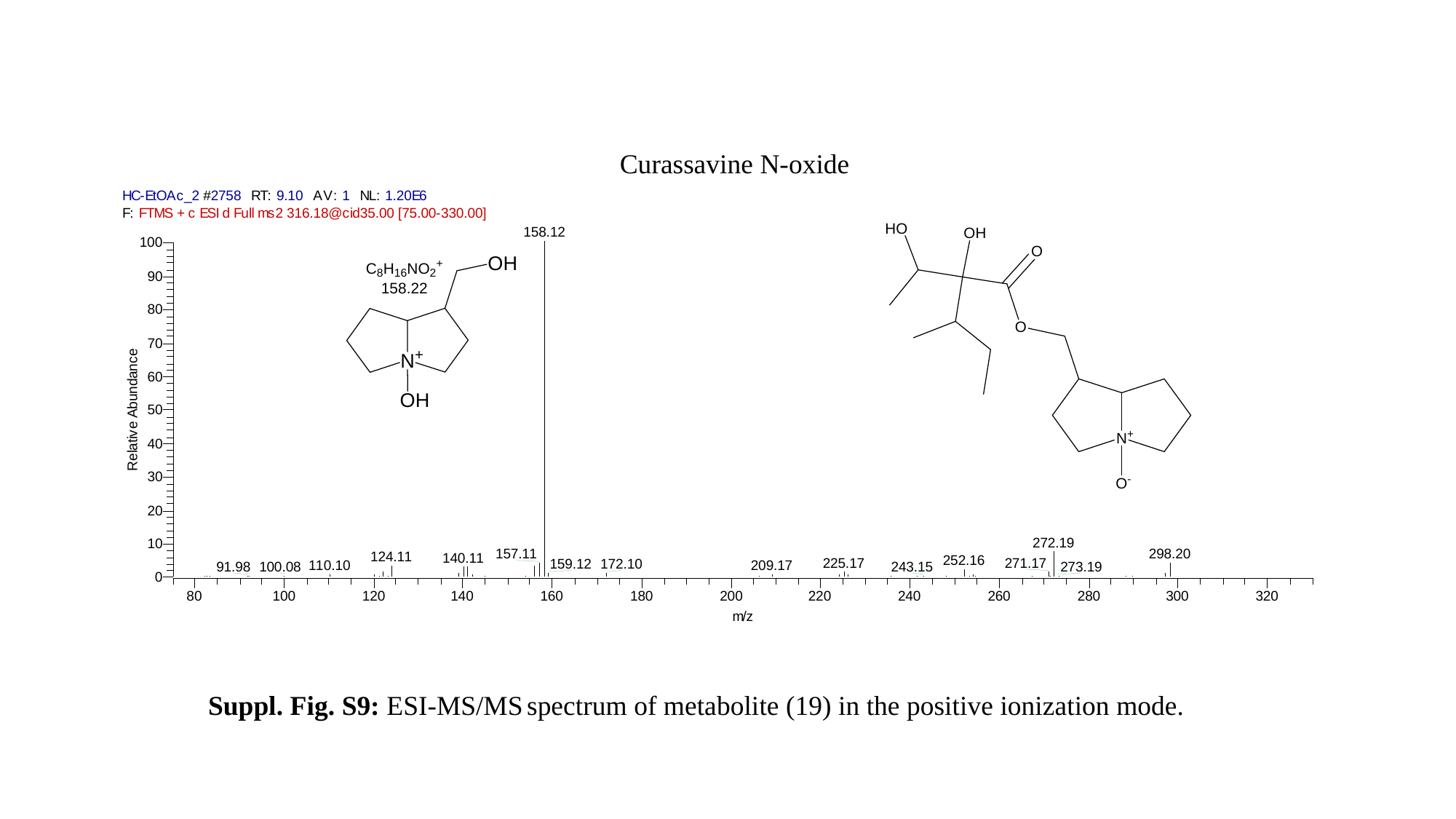

Curassavine N-oxide
Suppl. Fig. S9: ESI-MS/MS spectrum of metabolite (19) in the positive ionization mode.

## Slide 11
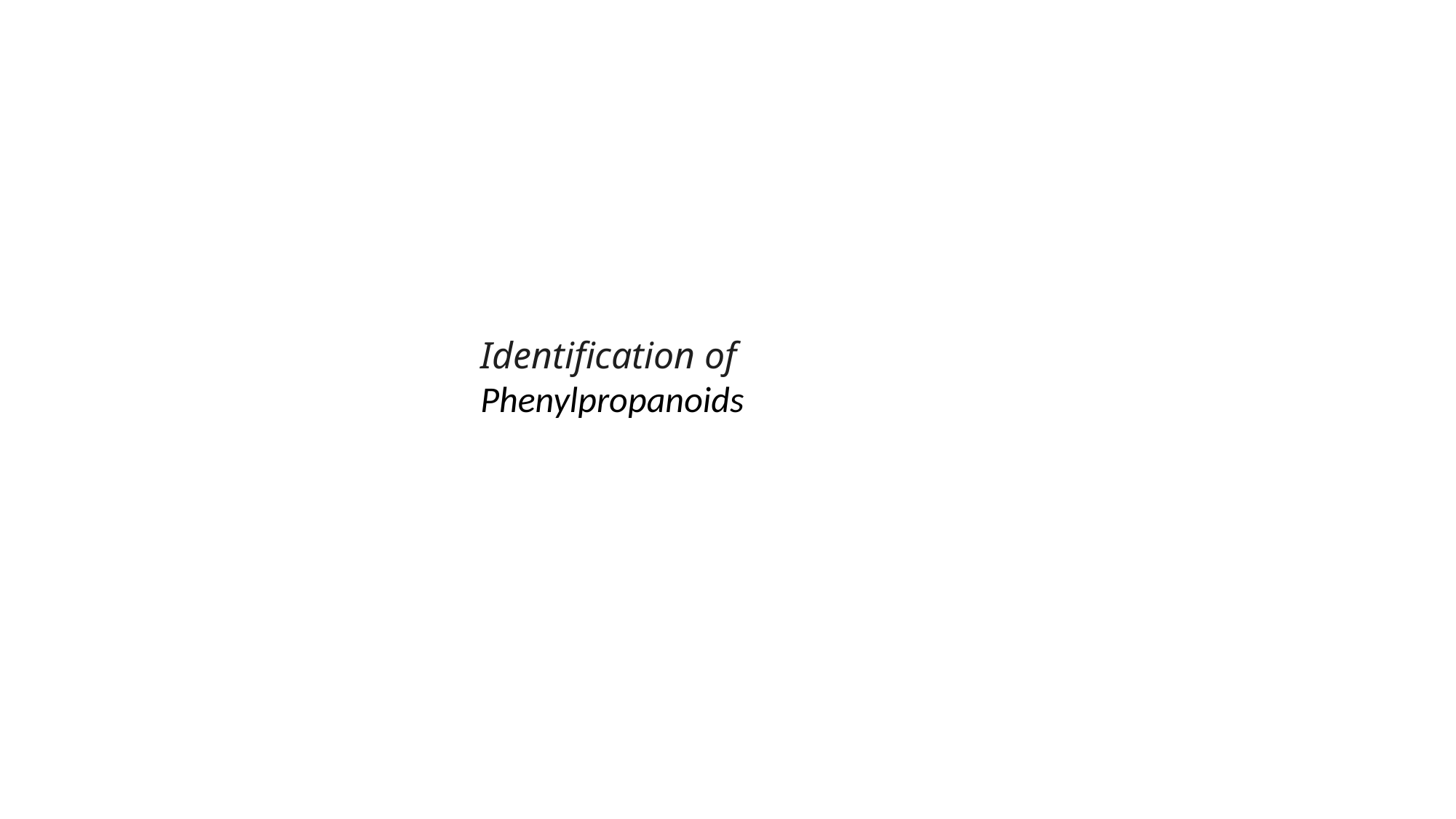

Identification of Phenylpropanoids

## Slide 12
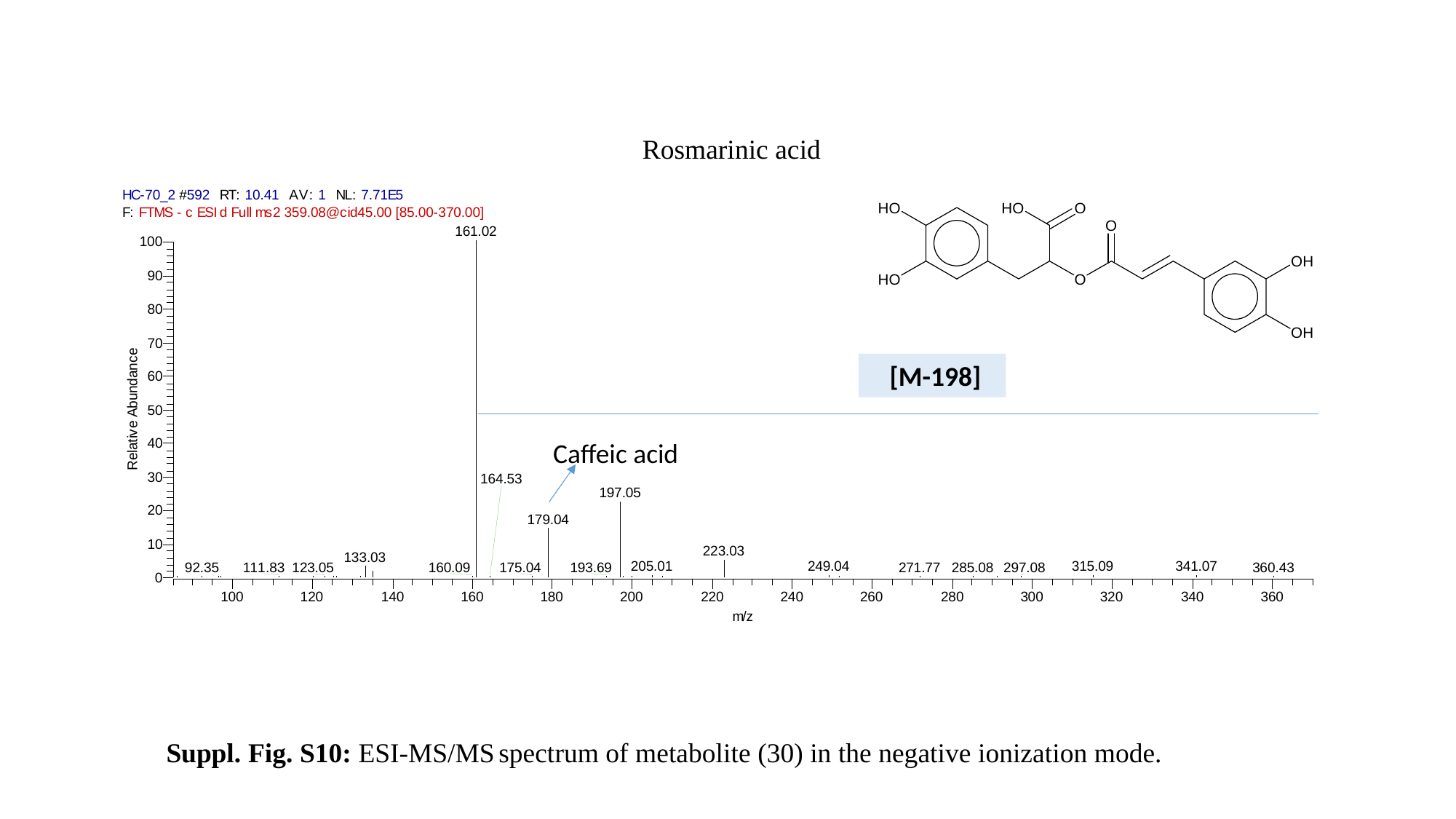

Rosmarinic acid
 [M-198]
Caffeic acid
Suppl. Fig. S10: ESI-MS/MS spectrum of metabolite (30) in the negative ionization mode.

## Slide 13
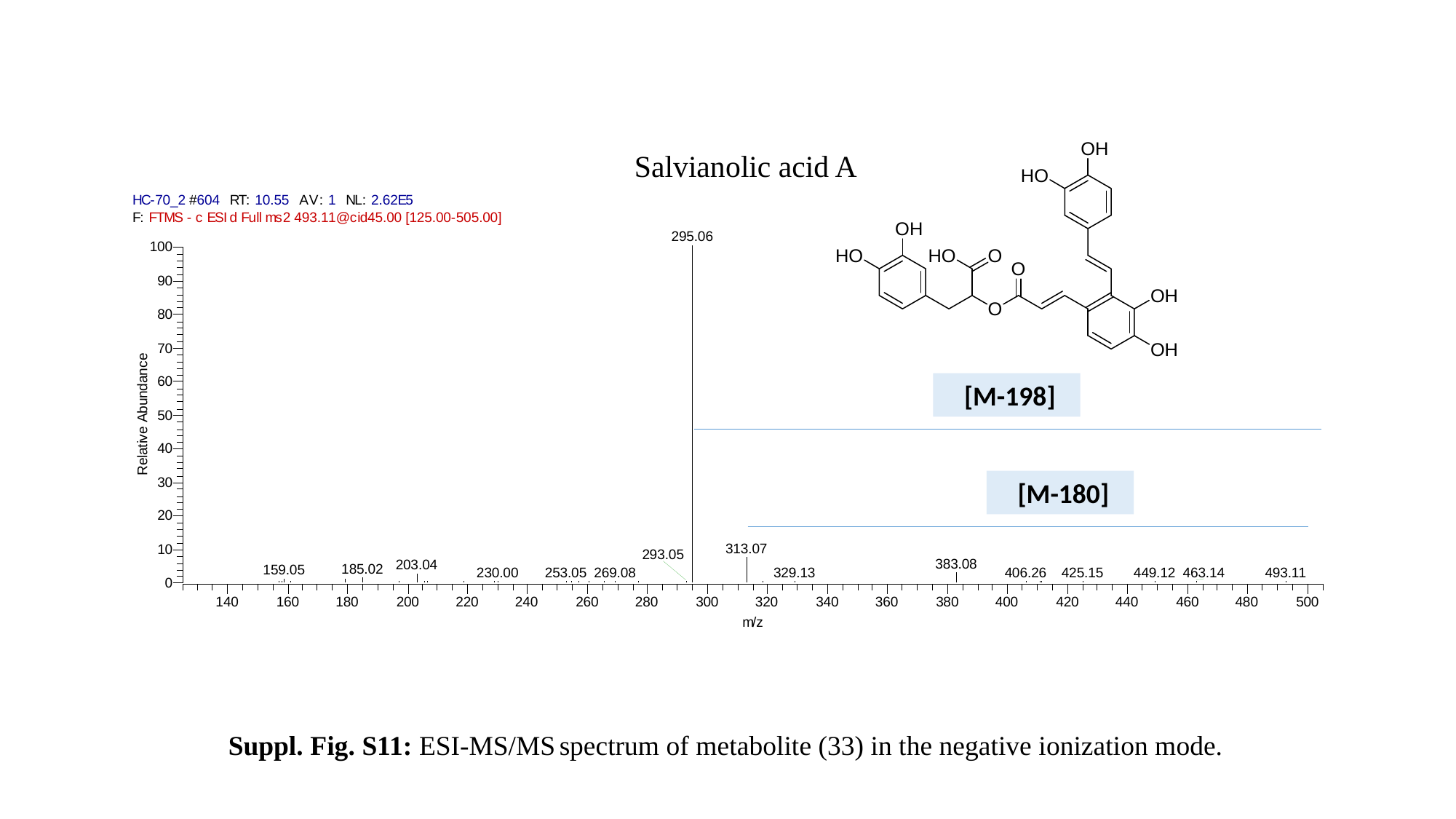

Salvianolic acid A
 [M-198]
 [M-180]
Suppl. Fig. S11: ESI-MS/MS spectrum of metabolite (33) in the negative ionization mode.

## Slide 14
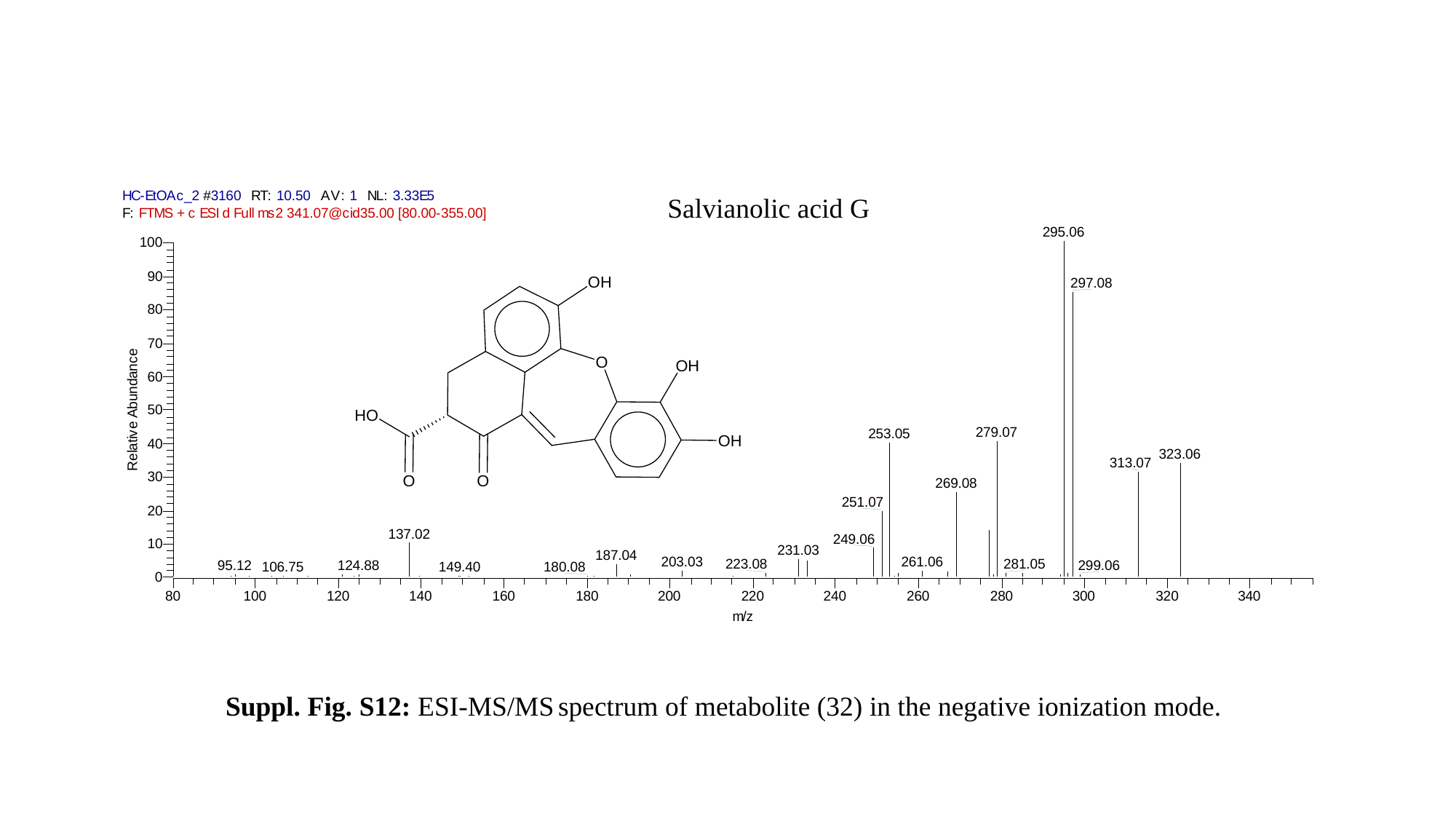

Salvianolic acid G
Suppl. Fig. S12: ESI-MS/MS spectrum of metabolite (32) in the negative ionization mode.

## Slide 15
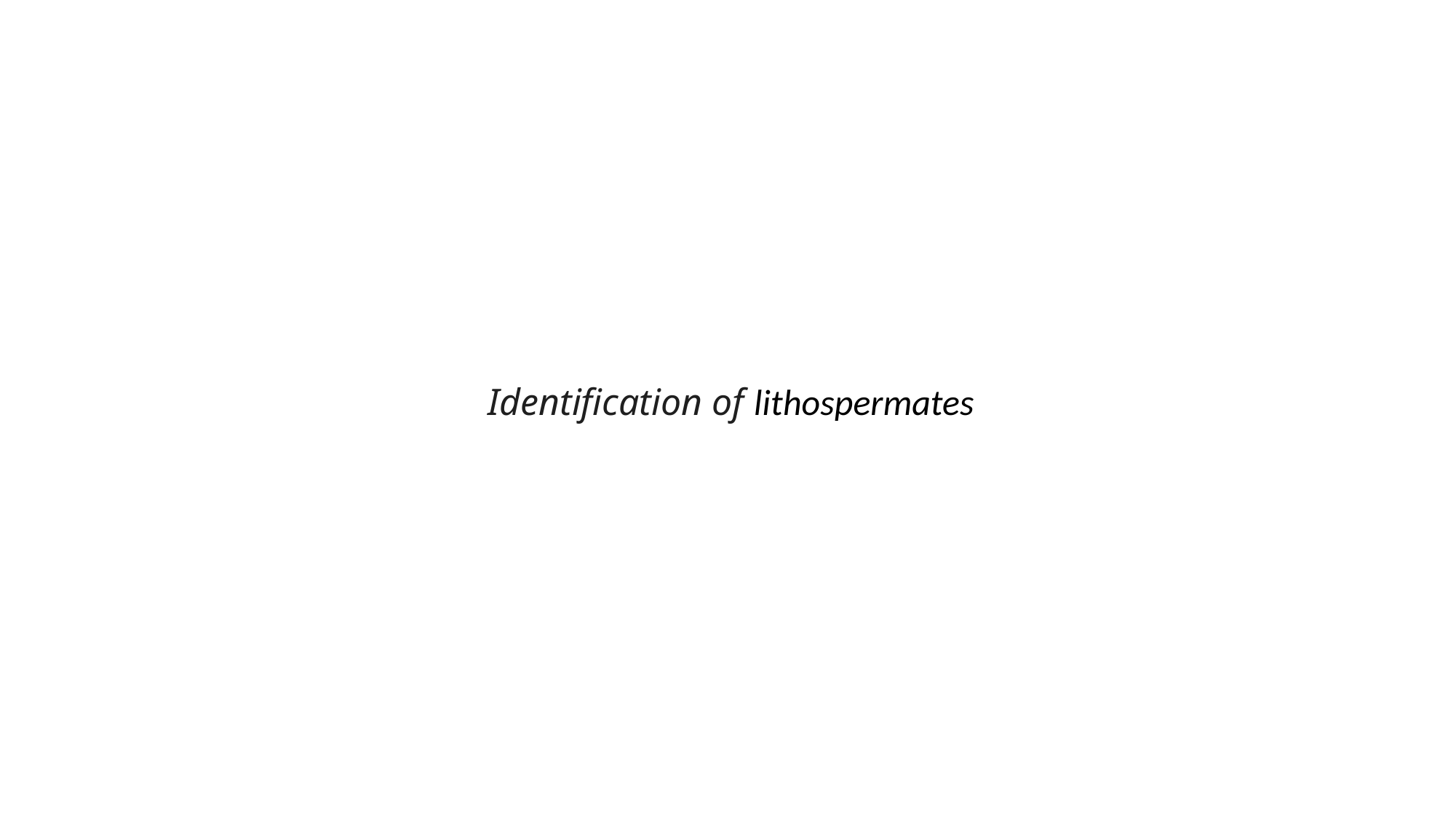

Identification of lithospermates

## Slide 16
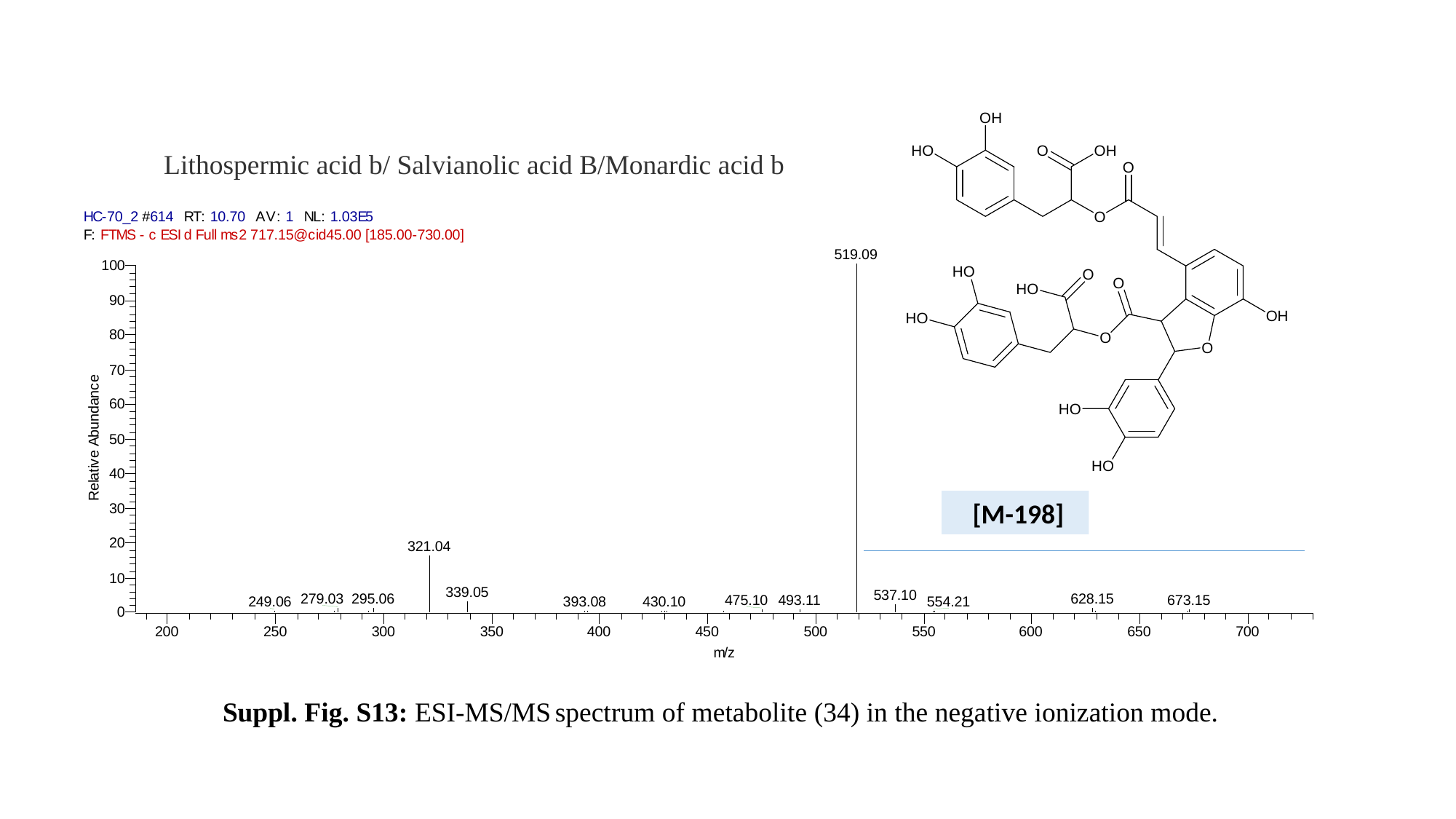

Lithospermic acid b/ Salvianolic acid B/Monardic acid b
 [M-198]
Suppl. Fig. S13: ESI-MS/MS spectrum of metabolite (34) in the negative ionization mode.

## Slide 17
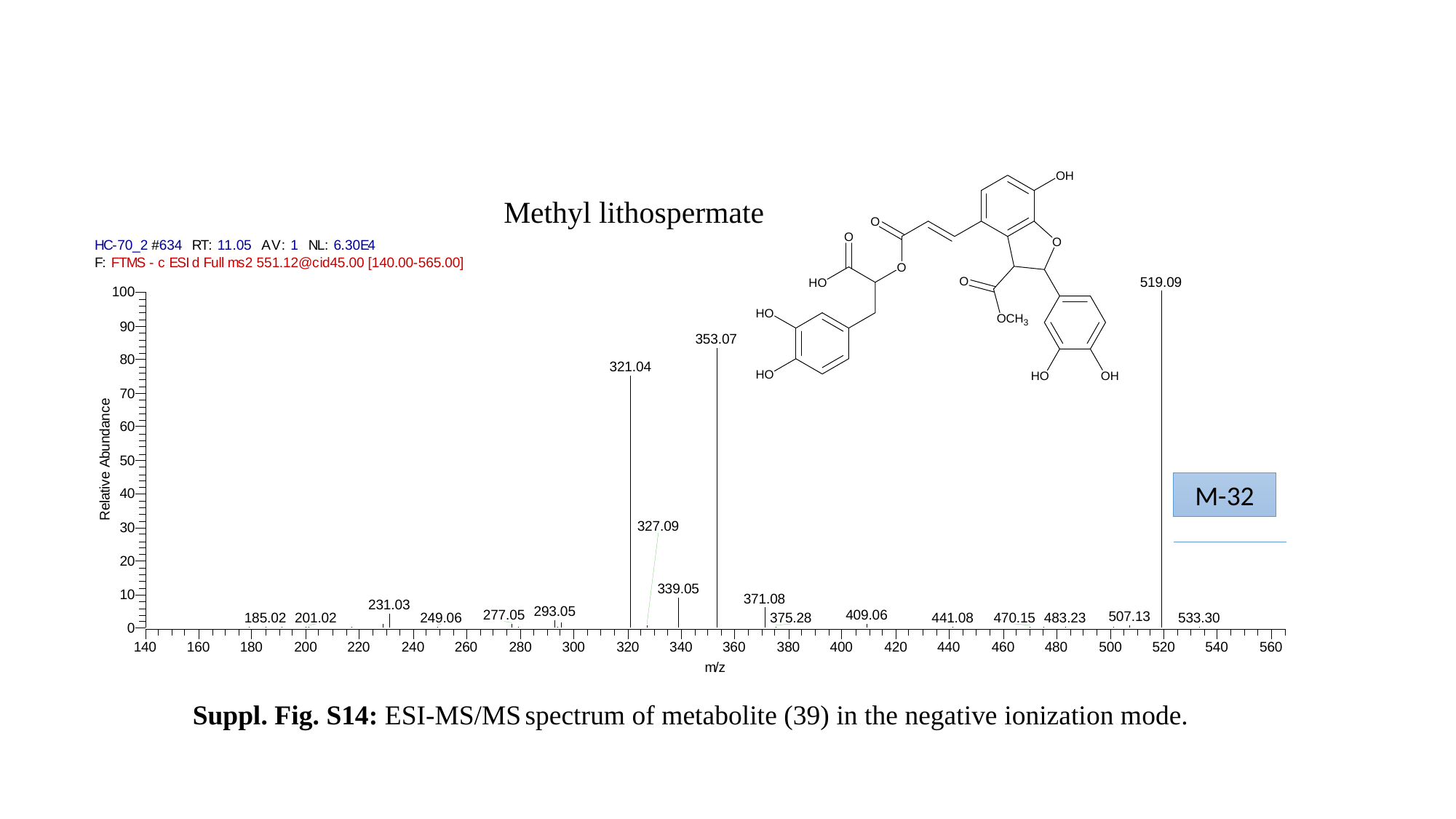

Methyl lithospermate
M-32
Suppl. Fig. S14: ESI-MS/MS spectrum of metabolite (39) in the negative ionization mode.

## Slide 18
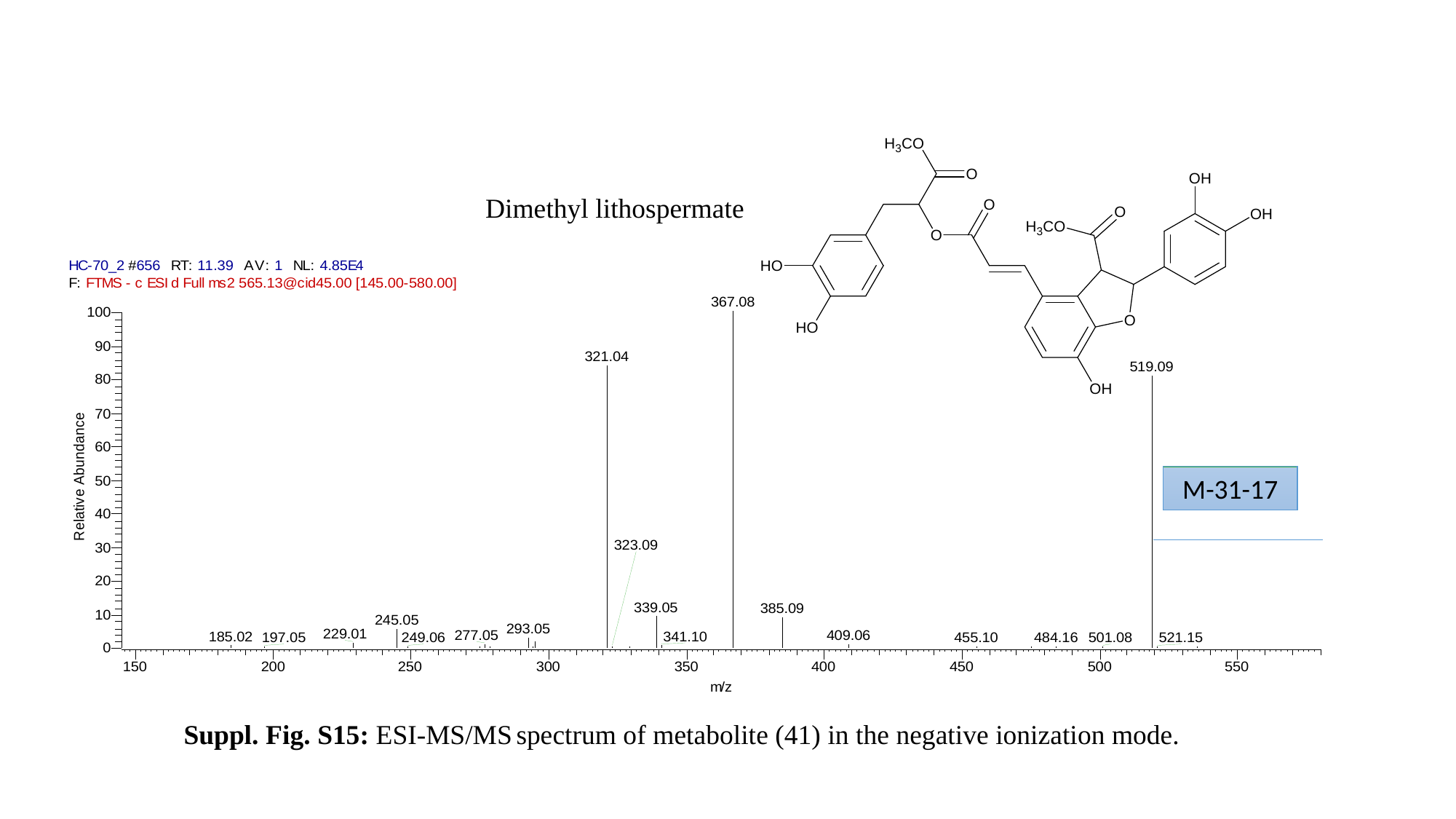

Dimethyl lithospermate
M-31-17
Suppl. Fig. S15: ESI-MS/MS spectrum of metabolite (41) in the negative ionization mode.

## Slide 19
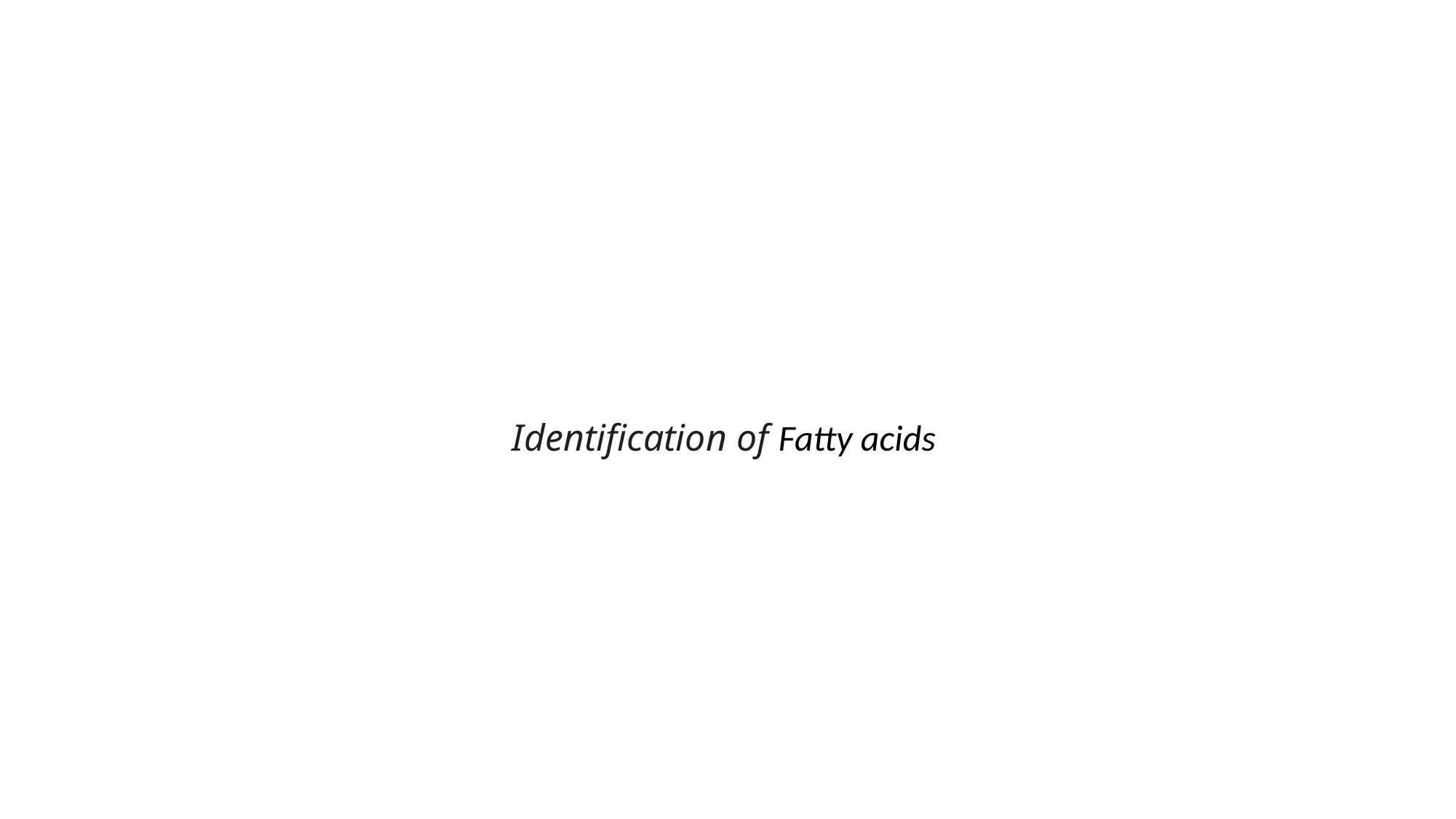

Identification of Fatty acids

## Slide 20
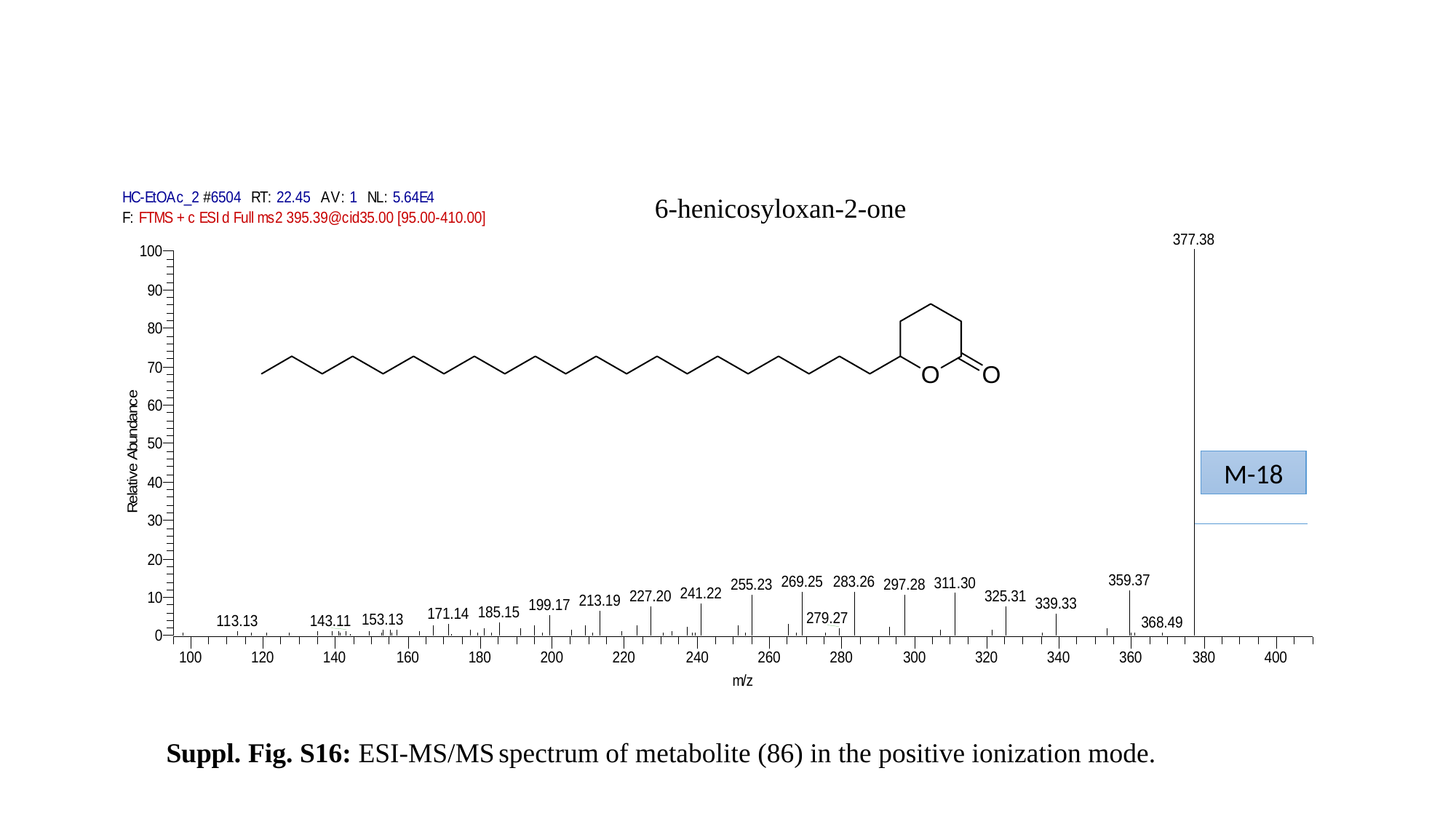

6-henicosyloxan-2-one
M-18
Suppl. Fig. S16: ESI-MS/MS spectrum of metabolite (86) in the positive ionization mode.

## Slide 21
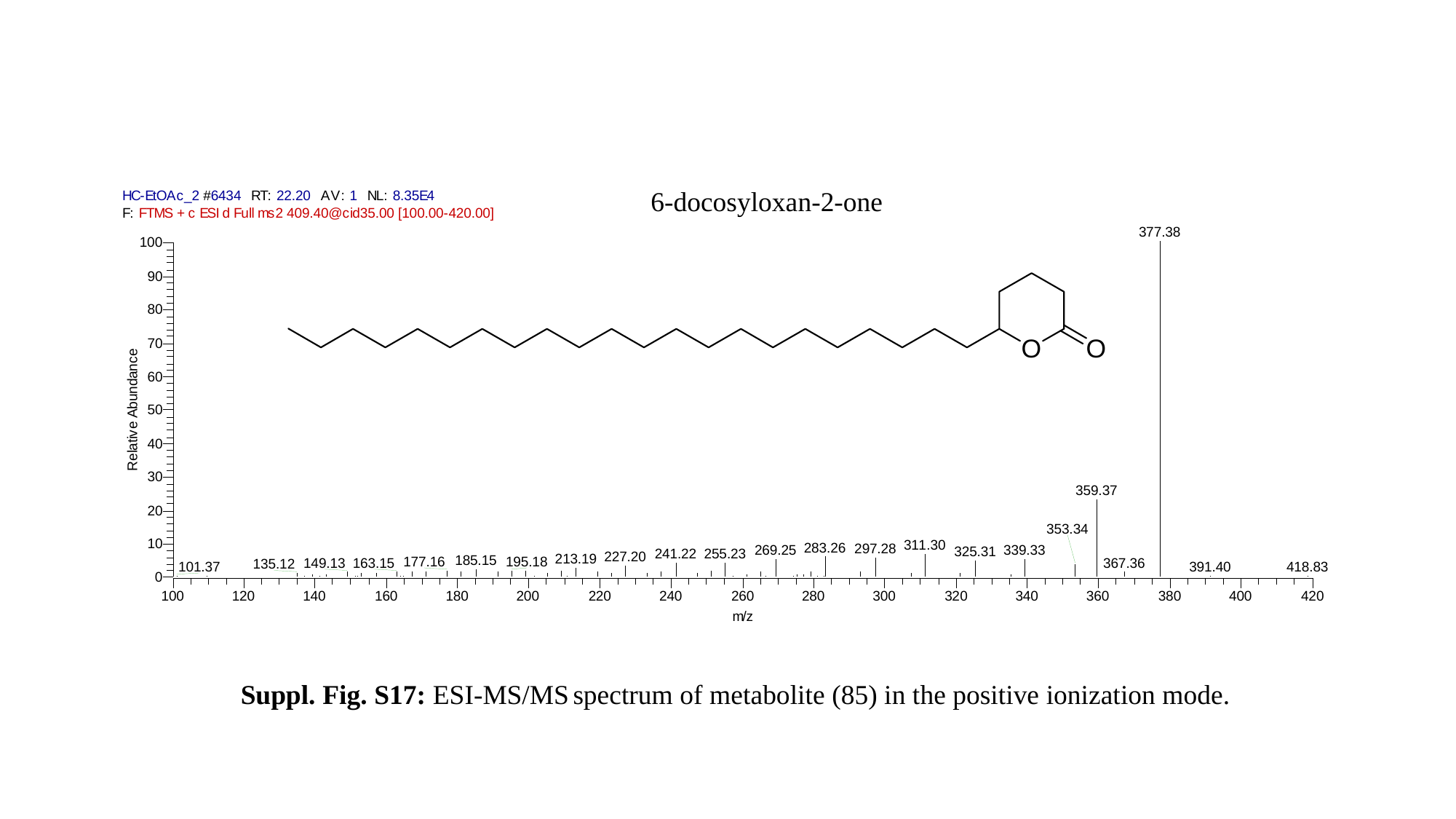

6-docosyloxan-2-one
Suppl. Fig. S17: ESI-MS/MS spectrum of metabolite (85) in the positive ionization mode.

## Slide 22
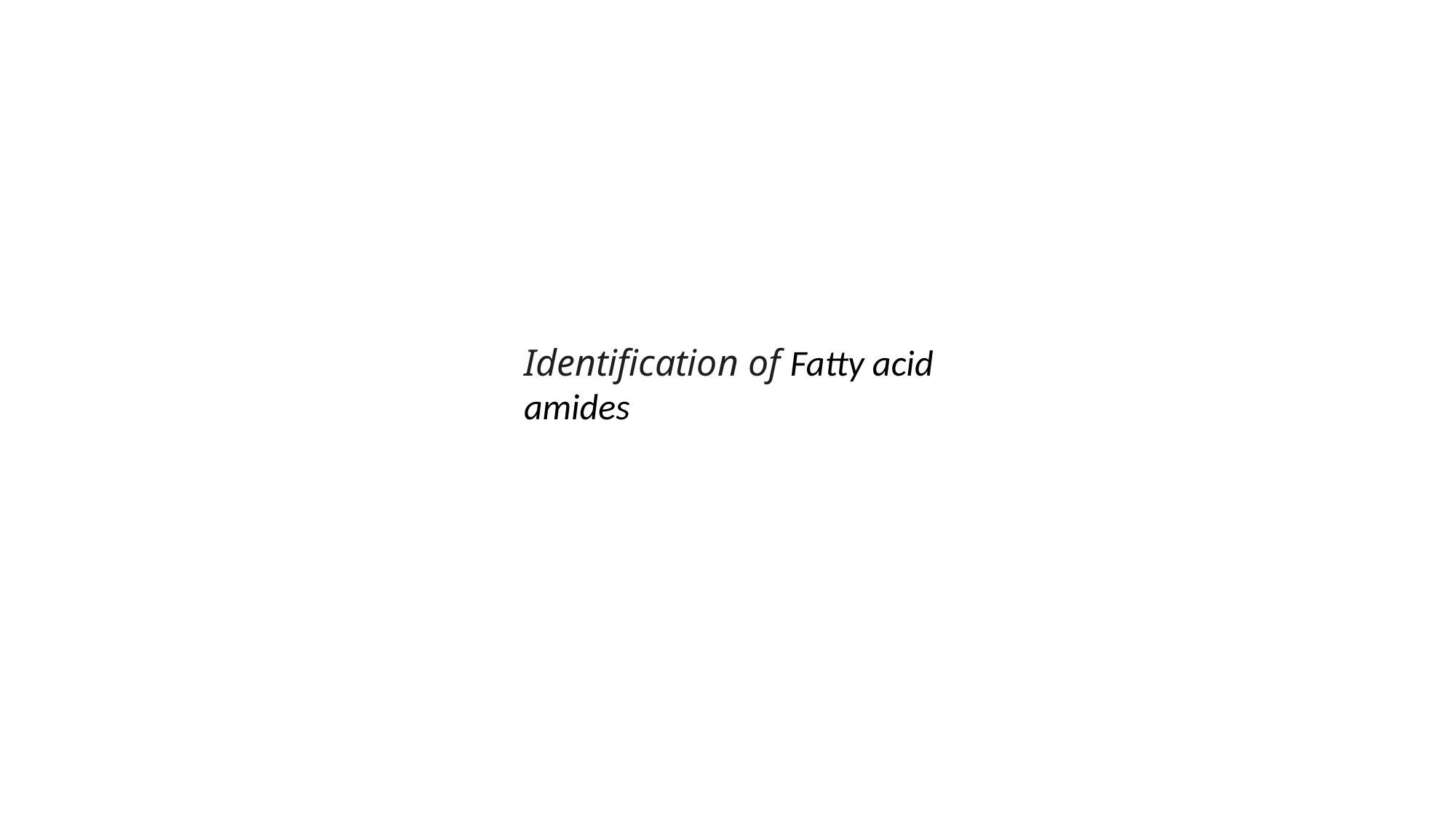

Identification of Fatty acid amides

## Slide 23
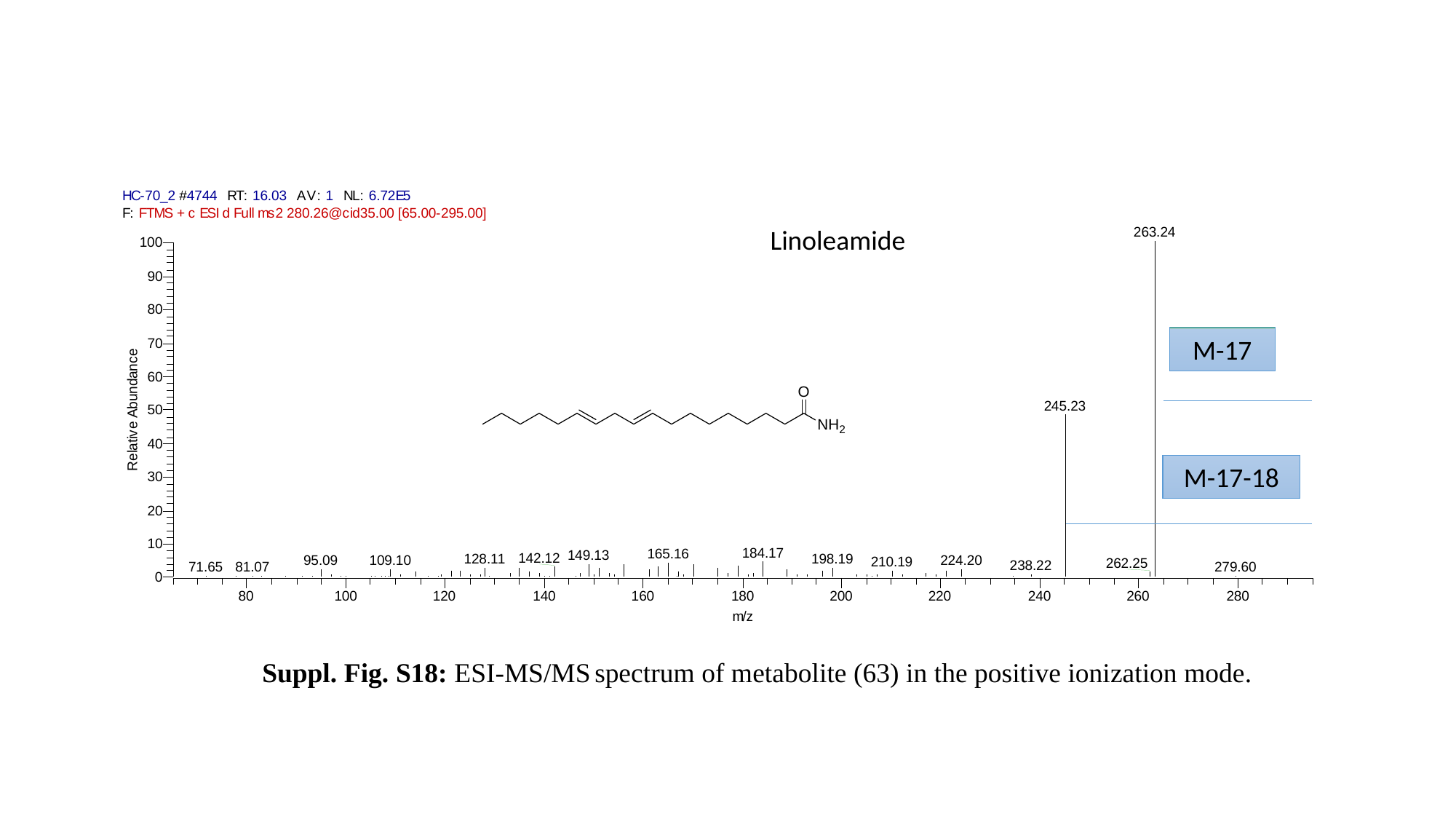

Linoleamide
M-17
M-17-18
Suppl. Fig. S18: ESI-MS/MS spectrum of metabolite (63) in the positive ionization mode.

## Slide 24
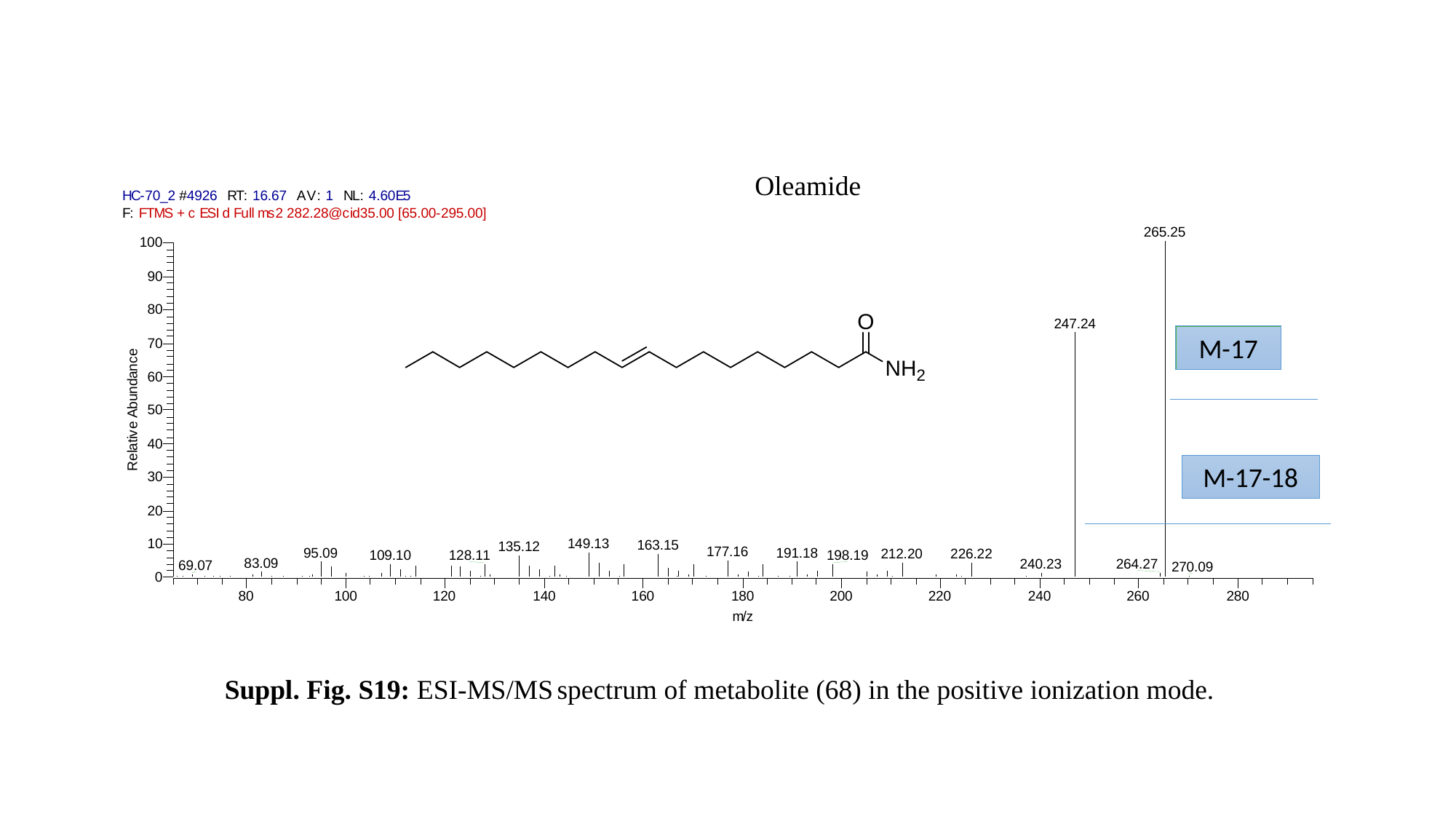

Oleamide
M-17
M-17-18
Suppl. Fig. S19: ESI-MS/MS spectrum of metabolite (68) in the positive ionization mode.

## Slide 25
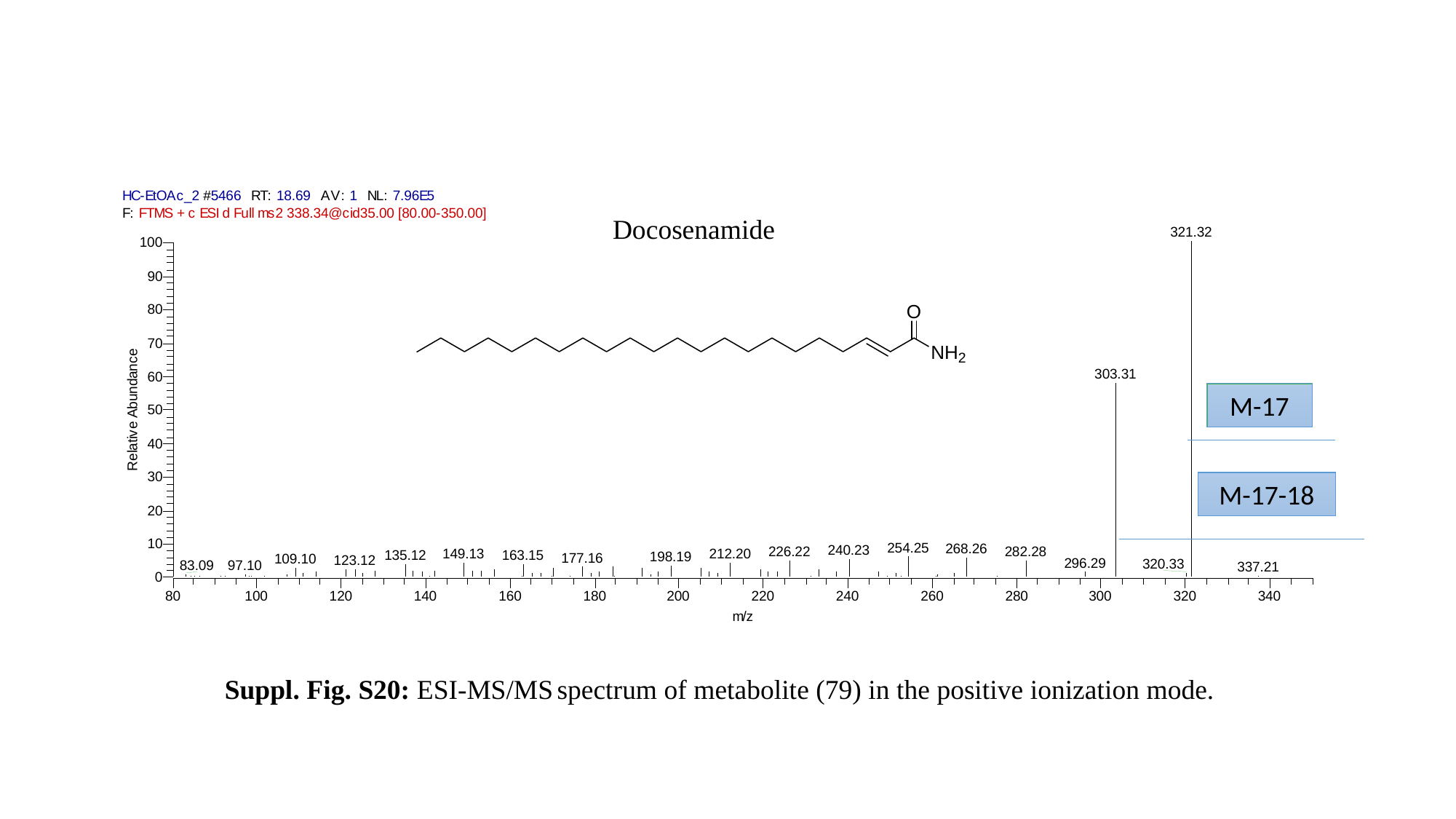

Docosenamide
M-17
M-17-18
Suppl. Fig. S20: ESI-MS/MS spectrum of metabolite (79) in the positive ionization mode.

## Slide 26
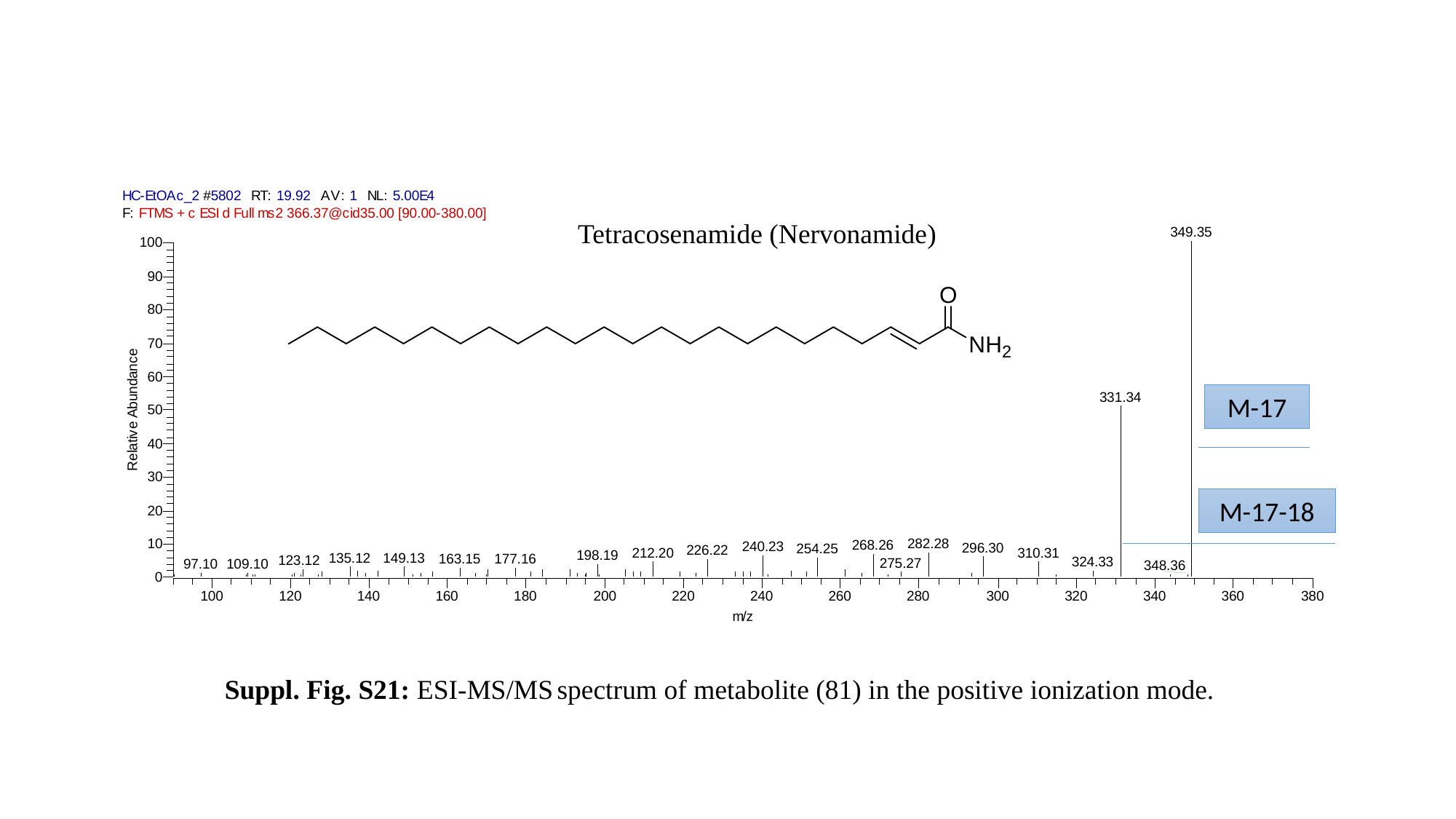

Tetracosenamide (Nervonamide)
M-17
M-17-18
Suppl. Fig. S21: ESI-MS/MS spectrum of metabolite (81) in the positive ionization mode.
